# Lower Number of Teeth Is Related to Higher Risks for ACVD and Death—Systematic Review and Meta-Analyses of Survival Data

**DOI:** 10.3389/fcvm.2021.621626

**Published:** 2021-05-07

**Authors:** Nicky G. F. M. Beukers, Naichuan Su, Bruno G. Loos, Geert J. M. G. van der Heijden

**Affiliations:** ^1^Academic Centre for Dentistry Amsterdam, Department of Periodontology, University of Amsterdam and Vrije Universiteit Amsterdam, Amsterdam, Netherlands; ^2^Academic Centre for Dentistry Amsterdam, Department of Social Dentistry, University of Amsterdam and Vrije Universiteit Amsterdam, Amsterdam, Netherlands; ^3^Amsterdam Public Health Research Institute, University of Amsterdam, Vrije Universiteit Amsterdam, Amsterdam, Netherlands

**Keywords:** systematic review, meta-analysis, number of teeth, tooth loss, atherosclerosis, cardiovascular disease, mortality, risk

## Abstract

Tooth loss reflects the endpoint of two major dental diseases: dental caries and periodontitis. These comprise 2% of the global burden of human diseases. A lower number of teeth has been associated with various systemic diseases, in particular, atherosclerotic cardiovascular diseases (ACVD). The aim was to summarize the evidence of tooth loss related to the risk for ACVD or death. Cohort studies with prospective follow-up data were retrieved from Medline-PubMed and EMBASE. Following the PRISMA guidelines, two reviewers independently selected articles, assessed the risk of bias, and extracted data on the number of teeth (tooth loss; exposure) and ACVD-related events and all-cause mortality (ACM) (outcome). A total of 75 articles were included of which 44 were qualified for meta-analysis. A lower number of teeth was related to a higher outcome risk; the pooled risk ratio (RR) for the cumulative incidence of ACVD ranged from 1.69 to 2.93, and for the cumulative incidence of ACM, the RR ranged from 1.76 to 2.27. The pooled multiple adjusted hazard ratio (HR) for the incidence density of ACVD ranged from 1.02 to 1.21, and for the incidence density of ACM, the HR ranged from 1.02 to 1.30. This systematic review and meta-analyses of survival data show that a lower number of teeth is a risk factor for both ACVD and death. Health care professionals should use this information to inform their patients and increase awareness on the importance of good dental health and increase efforts to prevent tooth loss.

## Introduction

In general, a lower number than 32 natural teeth in adults reflects the endpoint of dental caries and periodontitis. Tooth loss at a younger age is mainly due to caries, and in older ages, it is due to periodontitis. The cumulative incidence for caries peaks before the age of 30, while for periodontitis, it peaks between 20 and 40 years of age. The worldwide prevalence of severe tooth loss ( ≤9 remaining teeth) is 2.4% (1, 2). Tooth loss leads to reduced masticatory function, poorer nutritional status and unhealthy dietary changes, low self-esteem and quality of life, and negative general health (3–5). The burden of disease of severe caries, severe periodontitis, and the consequent tooth loss comprises 2% of the global burden of human diseases (6).

Apart from genetic and biological determinants, caries and periodontitis (and subsequent tooth loss) share several risk factors. In particular, hyposalivation, smoking, dysbiotic oral biofilms, and dietary fermentable carbohydrates contribute to their occurrence, while diabetes, obesity, and rheumatoid arthritis have been shown to be associated with both oral diseases (3, 4).

While material, behavioral, cultural, and psychosocial factors have been shown to contribute to the risk of both oral diseases and atherosclerotic cardiovascular diseases (ACVD) (7, 8), a biomedical connection between tooth loss and ACVD has also been investigated (9, 10). Shared pathways for oral diseases and ACVD have been proposed and studied; notably, short-lived bacteremias in periodontitis give rise to low-grade systemic inflammation and pro-inflammatory action and may contribute to the onset of atherosclerosis (11, 12). The atherosclerotic process may eventually lead to the occurrence of ACVD events (morbidity or mortality), like coronary heart diseases (CHD), cerebrovascular accidents, and peripheral arterial disease (13, 14). ACVD is responsible for up to 54% of deaths in the United States and 45% of deaths in Europe (15, 16).

Although the relationship between tooth loss and ACVD-related events and all-cause mortality (ACM) has been studied often, conflicting results have been reported, and solid evidence is lacking (17–20). Previously published systematic reviews did not allow for solid conclusions about the relationship between the number of teeth and ACVD, as they include a small number of studies (20) or restricted the outcome to ACM only (19). This systematic review and meta-analysis reports up-to-date evidence about the relationship between tooth loss, ACVD-related events, and ACM.

## Methods

This systematic review and meta-analyses were conducted in accordance with the guidelines of Preferred Reporting Items of Systematic Reviews and Meta-analyses (PRISMA Statement) and the guidelines for Meta-analysis Of Observational Studies in Epidemiology (MOOSE Guidelines) (Supplementary Files 1, 2) (21, 22).

### Research Question

Is tooth loss, particularly a lower number of present teeth, more related to an increased risk for ACVD-related events (morbidity or mortality) and ACM?

### Study Retrieval

Any study that evaluated the relationship between the number of teeth and ACVD-related morbidity, ACVD-related mortality, or ACM was retrieved from PubMed-Medline (National Library of Medicine, Washington, D.C.) and EMBASE (Excerpta Medical Database by Elsevier). We reported on studies included in these databases before June 17, 2020. The search was conducted by NB (for detail on the used search terms, see Supplementary File 3). There were no additional records identified through other sources, all studies were available, and, therefore, it was decided that there was no need to contact the authors of identified publications.

### Study Selection

We included cohort studies reported in the English language that evaluated a prospective relationship between the number of teeth and ACVD events or ACM at any follow-up time. Study participants, regardless of age, with a known (categorical or linear scaled) number of teeth at study inclusion were followed longitudinally to assess for the occurrence of ACVD events or ACM. Cumulative incidences and incidence densities were calculated. Events were based on information from hospital admissions (ICD codes, medical records), death certificates, self-report, or questionnaires. Cross-sectional studies, case-control studies, review articles, letters, personal opinions, book chapters, conference abstracts, patents, and articles written in a language other than English were excluded.

Two reviewers (NB and NS) independently screened titles of the retrieved studies based on the eligibility criteria mentioned above. The studies were categorized as definitely not eligible (notably, to be excluded), definitely eligible, or to be decided. Next, the reviewers screened abstracts for records, which were judged as to be decided. Subsequently, the same approach was followed for screening based on abstract. Thereafter, full texts were obtained for studies that met the inclusion criteria during screening based on the abstract or which then remained to be decided. Eligibility for final inclusion of publications was based on full text reading. Reviewers resolved initial disagreements by consensus discussion. For the reference list of the included studies, see Supplementary File 4.

### Risk of Bias Assessment

Two reviewers (NB and NS) used the ROBINS-E Tool (Risk Of Bias In Non-Randomized Studies—of Exposures) to assess the methodological quality and potential risk of bias of the included studies. The ROBINS-E tool comprises seven domains of bias: confounding, selection of participants into the study, classification of exposures, departures from intended exposures, missing data, measurement of outcomes, and selection of the reported result. Each domain was categorized into a low, moderate, or serious risk of bias. Judgments within each domain were summarized into an overall risk of bias assessment for each study (23). If one domain was judged as a serious risk of bias, the overall risk of bias of that study was assessed as a serious risk of bias. If all the domains were judged as low risk of bias, the overall risk of bias of that study was assessed as low risk of bias. Otherwise, the study was judged as a moderate risk of bias.

### Data Extraction

The following study data reported in the articles that met the selection criteria were extracted: author names, country, study design (inception or non-inception cohorts), the total number of participants and their demographic characteristics (age and sex), number of remaining teeth, number of participants with incident ACVD-related events or ACM, and follow-up time. Based on this information, it was possible to calculate the crude risk ratio (RR) with a 95% confidence interval (95% CI) for the cumulative incidence of ACVD-related events and ACM. Next to this, the crude and adjusted hazard ratio (HR) with 95% CI for the incidence density of ACVD-related events and ACM in different categories of the remaining number of teeth, if reported, were also obtained from the articles. The crude RR and HR were defined as the RR or HR of the number of teeth for ACVD-related events and ACM, not adjusted for other variables available in the included studies. The adjusted HR was defined as the HR adjusted for age and sex only or by multiple variables, such as education level, socioeconomic status, lifestyle habits or general health, etc., available in the included studies (see Supplementary Tables 1, 2).

The indicators for the potential risk of bias of the included studies contained the approaches to count the number of remaining teeth, patients' ACVD status at baseline, and follow-up rate. In the review, the approaches to counting the remaining number of teeth were classified into two categories: (1) based on clinical examination and (2) based on patients' self-reporting. Patients' ACVD status at baseline was classified into two categories: (1) ACVD status was presented and adjusted/excluded at baseline, and (2) ACVD status was presented without adjustment/exclusion at baseline or no information. The follow-up rate was classified into two categories: (1) low follow-up rate (<80%) and (2) high follow-up rate (≥80%). All the data were independently extracted by NB and NS, crossed-checked, and discussed to resolve possible disagreements.

### Data Analyses

Cumulative incidence is the measure of the occurrence of new ACVD-related events or ACM during the follow-up period. To assess the cumulative incidence of ACVD-related events and ACM between different groups of the number of remaining teeth, network meta-analyses with random effect models were carried out using the total number of study participants and the number of ACVD-related events or ACM.

Most of the included studies compared exposures (notably, the number of remaining teeth) using four common exposure categories:

- 0 teeth vs. 1–32 teeth,
- 0–19 teeth vs. 20–32 teeth,
- 0 teeth vs. 1–19 teeth vs. 20–32 teeth,
- 0–10 teeth vs. 11–16 teeth vs. 17–24 teeth vs. 25–32 teeth.

Some studies, using other than these four common categories, allowed transformation into the above, whereas the studies in which this was not possible were excluded for meta-analysis.

Two of the four common exposure categories included more than two groups. Therefore, network meta-analyses with random effect models were carried out (24). Network meta-analysis is defined as a meta-analysis comprising direct exposure comparisons within trials and indirect exposure comparisons across trials for a common comparator against at least two exposure groups (24). Because all the included studies in the present review reported data for all the exposure groups, the comparisons between any two groups in the network meta-analyses can be made directly. Therefore, the evidence from the indirect comparisons can be ignorable, and the assessment of ranking probabilities of each group and of the inconsistency between direct and indirect evidence was not necessary. The other two common exposure categories included two groups only, and, therefore, pair-wise meta-analyses were performed. Cumulative meta-analyses were used to assess the change in the aggregate estimate by adding study data in the order of the time of their publication (25). The results for studies reporting cumulative incidence data for ACVD-related events or ACM by common exposure category were statistically pooled and expressed as risk ratio (RR) and 95% confidence intervals (95% CI).

Incidence density (or hazard) is the measure for the rate of occurrence of new ACVD-related events or ACM per unit of time, the person-time of follow-up. It allows for pooling studies with different durations of follow-up. To compare the incidence density of ACVD-related events or ACM between different groups of the number of remaining teeth, generic inverse variance meta-analyses with random effect models were carried out to pool the hazard ratio (HR) of ACVD-related events or ACM of the individual studies. The HR was pooled separately based on two out of the four common categories in which the number of remaining teeth was classified only into two groups:

- 0 teeth vs. 1–32 teeth,
- 0–19 teeth vs. 20–32 teeth.

The other two common categories, in which the number of teeth was classified into more than two groups, were not included in the assessment of incidence density because the generic inverse variance meta-analysis only allows for the HR between two groups. Besides, the HR was pooled per number of lost teeth when the number of remaining teeth was regarded as a continuous variable. The HR was also pooled separately for different types of HR (crude HR, HR adjusted by age/sex, and HR adjusted by multiple variables). Cumulative meta-analyses were used to assess the change in the aggregate estimate by adding study data in the order of the time of their publication (25). The HR and standard errors (SE) of individual studies were log-transformed based on the formulas provided by Woods et al. (26). The pooled results were expressed as HR and 95% CI. The statistical heterogeneity of each meta-analysis was explored by the *I*^2^ test. The heterogeneity was considered substantial to considerable if *I*^2^ > 50% (27).

Funnel plots were used to assess the publication bias for the meta-analyses, which included at least 10 studies. A funnel plot for a meta-analysis including fewer than 10 studies is not recommended because of its insufficient statistical power (28).

The Grading of Recommendations Assessment, Development, and Evaluation (GRADE) system was used to appraise the strength of the evidence emerging from this review. Two reviewers (NB and NS) rated the strength of the evidence according to the factors that can reduce the quality of evidence (risk of bias, inconsistency of results, indirectness of evidence, imprecision, and publication bias) and the factors that can increase the quality of evidence (large magnitude of the effect, dose-response gradient, and effect of plausible residual confounding) (29). Any disagreement between the two reviewers was resolved by discussion.

All analyses were performed with the R software 3.3 (R Development Core Team, Vienna, Austria) and Review Manager 5.4 (The Nordic Cochrane Centre, The Cochrane Collaboration, Copenhagen, Denmark).

## Results

### Study Selection and Characteristics

**Figure 1:**
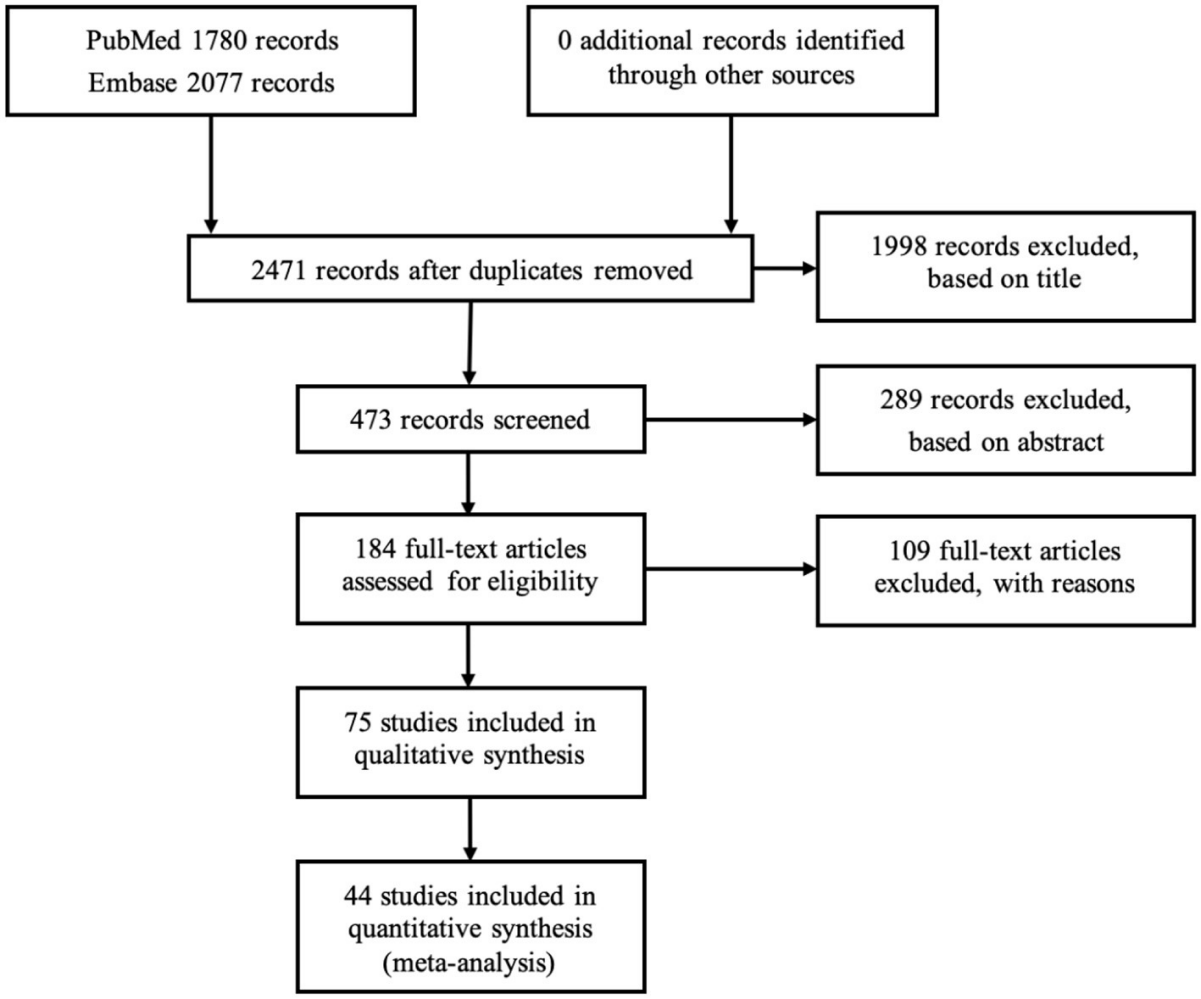
Flow diagram of the study selection process.

Figure 1 shows a flowchart of the study selection. The database search revealed 3,857 potential articles, of which 1,386 articles were removed for being duplicates. In the first screening based on title, 1,998 articles were excluded, leaving 473 articles for screening on the abstract; based on this, 289 articles were excluded, leaving 184 articles for full-text reading. Based on the full-text reading, another 109 articles were excluded, leaving 75 articles included for further analysis (for the reference list, see Supplementary File 3). The agreements between the two reviewers (NB and NS) based on the screening of titles, abstracts, and full-text reading were 92, 78, and 90%, respectively. Because the categories of the number of teeth in these 75 studies were too heterogeneous to allow for all to undergo transformation into any of the four common categories of the number of teeth, 44 articles were qualified for meta-analysis.

Table 1 shows the main characteristics of the 75 included articles for the qualitative synthesis. The articles were derived from all continents with the number of participants ranging from 173 to 4,440,970 [median: 5,688 and interquartile range (IQR): 718–41,000]. The majority of the studies included both males and females, whereas eight studies only included men, three studies only women, and, for one study, the male/female ratio was unknown. Only in 25 out of the 75 studies was the age of the study participants ≥60 years of age, whereas the other studies included participants from a wider age range. The maximum follow-up time ranged from 1 to 57 years. Most studies used clinical examination to assess the number of teeth (51 studies). For reporting of the outcome, 26 studies reported both ACVD-related events and ACM, 24 studies only reported ACM, and 25 studies only reported ACVD-related events. This information was mostly retrieved from medical records, death certificates, and death registers. All studies were prospective observational cohort studies, 67 studies reported findings from Cox proportional hazard analysis, five studies used logistic regression models, two studies used Poisson regression models, and one study applied a linear regression model. Exclusion of participants with prevalent ACVD at baseline was performed in 30 studies, and therefore, these studies could be defined as inception cohorts. In 21 studies, adjustment for prevalent ACVD at baseline was performed, whereas in 24 studies, no adjustment was performed, or no information for ACVD at baseline was available. The follow-up rate was high (≥80%) in 68 studies, low (<80%) in six studies, and unknown in one study (Table 1).

**Table 1:** Main characteristics of the included studies (*N* = 75).

**Author/year**	**Country**	**Study participants** ** (*N*)**	**Male/Female** ** (%)**	**Age (years)^a^**	**Follow-up time (years)^b^**	**Number of teeth**	**ACM and/or ACVD**			**ACVD at baseline**	**Follow-up rate^c^**
						**Source**	**Mortality**	**Morbidity**	**Source**		
Abnet et al. (30)	China	28,790	45/55	40–69	15	Clinical exam	ACM + ACVD		Clinical exam by village doctors	Unknown	High
Adolph et al. (31)	France	76,188	64/36	16–89	Mean: 3.4 (SD = 2.4)	Clinical exam	ACM		Death certificates	Present, excluded	High
Aida et al. (32)	Japan	4,425	49/51	≥65	5	Self-reported	ACVD		Death register	Present, adjusted	High
Ajwani et al. (33)	Finland	364	28/72	76, 81, or 86	10	Clinical exam	ACM + ACVD		Death register	Present, adjusted	Low
Ajwani et al. (34)	Finland	175	31/69	76, 81, or 86	5	Clinical exam	ACM + ACVD		Death register	Present, adjusted	High
Ando et al. (35)	Japan	7,779	Only men	40–79	7	Self-reported	ACM + ACVD		Register sheets at local government offices and death certificates	Present, excluded	High
Ansai et al. (36)	Japan	697	40/60	80	6	Clinical exam	ACM		Registers at the Public Health Centers	Present, no adjustment or exclusion	High
Batty et et al. (37)	Korea	975,685	64/36	Mean: Men: 44.0 (±10.8) Women: 48.6 (±11.5)	21	Clinical exam	CHD	CHD	Death certificates and health insurance claims	Present, excluded	High
Brown et al. (38)	USA	41,000	53/47	≥18	16	Self-reported	ACM + ACVD		Death register	Present, adjusted	High
Cabrera et al. (39)	Sweden	1,462	Only women	38, 46, 50, 54, 60	24	Clinical exam	ACM + ACVD	ACVD	Death certificates and self-reported history and medical exam	Present, no adjustment or exclusion	High
Caplan et al. (40)	USA	535	30/70	60–103	7	Clinical exam	ACM		Death certificates	Unknown	Low
Chang et al. (41)	Korea	161,286	61/39	40–79	Median: 10.5	Clinical exam		Heart failure	Medical records	Present, excluded	High
Chang et al. (42)	Korea	206,602	59/61	40–79	Median: 10.4	Clinical exam		Stroke	Medical records	Present, excluded	High
Choe et al. (43)	Korea	867,256	78/22	≥30	14	Clinical exam	Stroke	Stroke	Health insurance claims and death certificates	Present, excluded	High
Darnaud et al. (44)	France	85,830	61/39	16–94	14	Clinical exam	ACM +ACVD		Death register and death certificates	Present, excluded	High
Del Brutto et al. (45)	Ecuador	718	44/56	≥40	4	Clinical exam	Stroke	Stroke	Death certificates and questionnaire and confirmation diagnosis by certified neurologists	Present, excluded	High
Dewake et al. (46)	Japan	173	14/86	62–102	1	Clinical exam	ACM		Medical records	Unknown	High
Dietrich et al. (47)	USA	1,203	Only men	21–84	35	Clinical exam	ACVD	ACVD	Medical records and medical exam	Present, excluded	High
Fukai et al. (48)	Japan	5,688	40/60	>40	15	Clinical exam	ACM + ACVD		Public health center register	Present, excluded	High
Furuta et al. (49)	Japan	281	25/75	≥65	3	Clinical exam	ACM		Home-based care support centers	Unknown	High
Garcia et al. (50)	USA	804	Only men	25–85	28	Clinical exam	ACM		Death certificates	Present, excluded	High
Goto et al. (51)	Japan	11,273	44/66	45–79	11	Self-reported	ACM + ACVD		Death certificates	Present, excluded	High
Hamalainen et al. (52)	Finland	226	29/71	80	10	Clinical exam	ACM		Death register	Present, adjusted	High
Hayasaka et al. (53)	Japan	21,730	42/58	≥65	4	Self-reported	ACM		Death register	Present, adjusted	High
Heitmann et al. (54)	Denmark	2,932	50/50	Unknown	12	Clinical exam	ACVD	ACVD	Death register, national patient registry of hospital discharges and central person register	Present, excluded	High
Hiratsuka et al. (55)	Japan	891	46/54	≥70	13	Clinical exam	ACM		Death register	Unknown	High
Hirotomi et al. (56)	Japan	569	51/49	70	5	Clinical exam	ACM		Unknown	Present, adjusted	High
Hoke et al. (57)	Austria	411	66/34	Median: 69 (IQR: 62–76)	Median: 6.2 (IQR: 5.8 – 6.6)	Clinical exam of edentulism	ACM + ACVD		Death register	Present, excluded and adjusted^¥^	High
Holm-Pedersen et al. (58)	Denmark	573	48/52	70	20	Clinical exam	ACM		Death register	Present, adjusted	High
Holmlund et al. (59)	Sweden	7,674	43/57	20–89	Median: 12 (IQR 0.2–29)	Clinical exam	ACM + ACVD		Death register	Present, adjusted^Ω^	High
Holmlund et al. (60)	Sweden	8,999	43/57	20–85	33	Clinical exam	ACM		Death register	Present, excluded	High
Hu et al. (61)	Taiwan	55,651	47/53	≥65	5	Clinical exam	ACM		Death register	Unknown	High
Hung et al. (62)	USA	HPFS: 41,407 NHS: 58,974	41/59	HPFS: 40–75 NHS: 30–55	12	Self-reported	CHD	CHD	Medical records, hospital records, autopsy reports and death certificates	Present, excluded	High
Hung et al. (63)	USA	45,094	Only men	40–75	12	Self-reported		PAD	Questionnaires and confirmed by medical records if possible	Present, excluded	High
Iwasaki et al. (64)	Japan	273	50/50	80	3	Clinical exam		Stroke	Stroke-related costs and hospitalization obtained by health insurance claims	Present, excluded and adjusted	Low
Janket et al. (65)	Finland	473	63/37	Unknown	Median: 15.8	Clinical exam	ACM + ACVD		Death register	Present, no adjustment or exclusion^♢^	High
Janket et al. (66)	Finland	461	63/37	Unknown	Median: 15.8	Clinical exam	ACVD		Death register	Present, no adjustment or exclusion^♢^	High
Joshipura et al. (67)	USA	41,380	Only men	40–75	12	Self-reported		Stroke	Medical records	Present, excluded	High
Joshipura et al. (68)	USA	44,119	Only men	40–75	6	Self-reported	CHD	CHD	Medical records	Present, excluded	High
Joshy et al. (69)	Australia	167,697	43/57	45–75	5	Self-reported	ACM	ACVD	Death register and medical records	Present, excluded	High
Kebede et al. (70)	Germany	3,327	Only men	20–81	14	Clinical exam	ACM + ACVD		Death register	Unknown	High
Kim et al. (71)	USA	588	49/51	≥40	7	Clinical exam	ACM		Death register	Present, adjusted	High
LaMonte et al. (72)	USA	57,001	Only women	50–79	12	Self-reported	ACM + ACVD	ACVD, CHD, Stroke	Annual mailed follow-up questionnaires and medical record review	Present, excluded	High
Lee et al. (73)	Korea	4,440,970	62/38	Mean 41.5	9	Clinical exam	ACM	MI, Stroke, Heart Failure	Death register and health check-ups	Present, excluded	High
Li et al. (74)	USA	10,958	58/42	55–88	5	Clinical exam	ACM + ACVD	ACVD	Certification, autopsy report, clinical notes and medical exam	Present, adjusted	High
Liljestrand et al. (75)	Finland	7,629	49/51	25–74	13	Clinical exam	ACM	ACVD	Death register and drug reimbursement records and hospital discharge register	Present, adjusted	High
Matsuyama et al. (76)	Japan	77,397	47/53	>65	1,374 days	Self-reported	ACM		Death register	Unknown	High
Morita et al. (77)	Japan	59 patients ≥20 teeth, 59 matched patients <20 teeth	41/59	≥80	10	Self-reported	ACM		Death register	Present, no adjustment or exclusion	High
Morrison et al. (78)	Canada	9,331 (CHD), 10,120 (ACVD)	46/54	35–84	23	Clinical exam	CHD + ACVD		Death register	Present, excluded	Unknown
Mucci et al. (79)	USA	15,273 twins	Unknown	35	37	Self-reported	ACVD	ACVD	Death register and medical records	Present, excluded	High
Munoz-Torres et al. (80)	USA	79,663	Only women	Mean age per category number of teeth	16	Self-reported		PAD	Self-reported and confirmed by medical records	Present, excluded	High
Noguchi et al. (81)	Japan	3,081	Only men	36–59	5	Self-reported		MI	Self-reported	Present, excluded	Low
Nomura et al. (82)	Japan	608	38/62	80	20	Clinical exam	ACM		Death register	Unknown	High
Oluwagbemigun et al. (83)	Germany	24,313	38/62	35–64	13	Self-reported		MI + stroke	Self-reported and validated by medical records	Present, adjusted	High
Österberg et al. (84)	Denmark, Finland, Sweden	1,004	43/57	75	7	Self-reported	ACM		Death register	Present, adjusted	High
Österberg et al. (85)	Sweden	1,803	47/53	70	18	Clinical exam	ACM		Death register	Present, adjusted	Low
Padilha et al. (86)	USA	500	82/18	Mean: 58 (±17)	26	Clinical exam	ACM		Telephone follow-up, correspondence from relatives, and annual searches of death register	Present, adjusted	High
Paganini-Hill et al. (87)	USA	5,611	31/69	52–105	17	Self-reported	ACM		Death register and death certificates	Present, adjusted	High
Park et al. (88)	Korea	247,696	58/42	46–60	10	Clinical exam	ACVD		Death register and death certificates	Present, excluded	High
Qi et al. (89)	China	1,385	48/52	>75	4	Clinical exam	ACM + ACVD		Death register	Unknown	High
Ragnarsson et al. (90)	Iceland	2,613	47/53	25–79	ACM: 15 CHD: 8	Clinical exam	ACM + CHD		Death register	Unknown	High
Reichert et al. (91)	Germany	942	74/26	≥18	1	Clinical exam	Combined endpoint^∧^	Combined endpoint^∧^	Questionnaire, telephone interview, civil registration offices, medical records	Present, no adjustment or exclusion^ß^	High
Reichert et al. (92)	Germany	953	74/26	≥18	3	Clinical exam	Combined endpoint^∧^	Combined endpoint^∧^	Civil registration offices, medical records, physicians and relatives	Present, no adjustment or exclusion^ß^	High
Saito et al. (93)	Japan	4,700	45/55	75 and 80	2	Clinical exam		ACVD	Health insurance claims	Present, no adjustment or exclusion	High
Schwahn et al. (94)	Germany	1,803	50/50	Median: 64 (IQR: 17)	12	Clinical exam	ACM + ACVD		Death certificates	Unknown	High
Shimazaki et al. (95)	Japan	1,762	28/72^¶^	59–107	6	Clinical exam	ACM		Medical records or interviews with study participants' relatives	Present, adjusted	Low
Soikkonen et al. (96)	Finland	292	29/71	76, 81, and 86	4	Clinical exam	ACM		Unknown	Unknown	High
Tu et al. (97)	UK	10,592	78/22^¶^	≤30	57	Clinical exam	ACM + ACVD + CHD + Stroke		Death register	Unknown	High
Tuominen et al. (98)	Finland	6,527	47/53	30–69	Mean: 12	Clinical exam	ACM + CHD		Death register	Unknown	High
Vedin et al. (99)	39 Countries on 5 Continents	15,456	81/19	≥60	Median: 3.7	Self-reported	Primary outcome^#^ Secondary outcome^§^	Primary outcome^#^ Secondary outcome^§^	Medical records and medical exam	Present, adjusted^∑^	High
Vedin et al. (100)	39 Countries on 5 Continents	15,456	81/19	≥60	Median: 3.7	Self-reported	Primary outcome^#^ + ACVD death	Primary outcome^#^ + Stroke	Medical records and medical exam	Present, adjusted^∑^	High
Vogtmann et al. (101)	Iran	50,023	42/58	40–75	10	Self-reported	ACM + ACVD		Interview *via* family-members, death certificates and other medical documents	Present, excluded	High
Watt et al. (102)	UK	12,871	44/56	≥35	12	Self-reported	ACM + ACVD + CHD + Stroke		Clinical exam and death certificate	Unknown	High
Wu et al. (103)	USA	9,962	48/52	25–74	21	Clinical exam	CVA	CVA	Death certificates and medical records	Present, excluded	High
Yuan et al. (104)	China	36,153	41/59	Median: 90 (IQR: 81–99)	Median: 3 (IQR: 1.6–5.7)	Self-reported	ACM		Interview with close family-member	Present, adjusted	High

*ACM, all-cause mortality; ACVD, atherosclerotic cardiovascular disease; CHD, coronary heart disease; MI, myocardial infarction; CVA, cerebrovascular accident; PAD, peripheral arterial disease; HPFS, Health Professionals Follow-up Study; NHS, Nurses' Health Study*.

*All studies (n = 75) use as statistical analysis Cox proportional hazard regression model, except for the following studies: Ansai et al. (36), Dewake et al. (46), Kim et al. (71), Noguchi et al. (81), Shimazaki et al. (95) (Logistic regression model), Del Brutto et al. (45), Morrison et al. (78) (Poisson regression model), Iwasaki et al. (64) (Linear regression model)*.

a *The reported age range applies to the baseline examination*.

b *The reported follow-up time is the maximum time in years if reported*.

c *The follow-up rate was classified into two categories: (1) low follow-up rate (<80%) and (2) high follow-up rate (≥80%)*.

∧ *Combined endpoint: MI and stroke/TIA, cardiac-related and stroke-related mortality*.

# *Primary outcome: Major Adverse Cardiovascular Events (MACEs) including a composite of the first occurrence of ACVD death, non-fatal MI, or non-fatal stroke*.

§ *Secondary outcome: Non-fatal or fatal MI; Non-fatal or fatal stroke; ACVD death; ACM*.

¶ *The percentage male/female applies to the included number of study participants*.

*The percentage male/female is based on the outcome for CHD*.

¥ *The cohort consists of study participants with prevalent atherosclerotic carotid artery disease, as defined by the presence of non-stenotic plaque or carotid stenosis of any degree. Patients with an ACVD-event (MI/stroke/coronary revascularization/peripheral vascular surgery) during the preceding 6 months were excluded. For history of MI, PAD, history of stroke, baseline degree of carotid stenosis, adjustment in the analyses were performed*.

Ω *In a subgroup analysis performed in 4,164 study participants in whom a history of previous MI and hypertension (drug-treated) was collected, the relationship between the number of teeth and future ACVD death was essentially unaltered compared to the analysis in the total sample when previous MI and hypertension were added as confounders in the analysis*.

♢ *Baseline data consisted of 256 coronary artery disease patients and 250 age and sex-matched controls and created a prospective follow-up study*.

ß *Known CHD is in the inclusion criteria*.

∑ *Patients were eligible to participate if they had CHD, but the target patients of this paper are the patients with stable CHD. In the statistical analyses, correction for ACVD was done*.

Supplementary Tables 1, 2 show the descriptive information and summary results of the studies regarding tooth loss and ACVD-related events (Supplementary Table 1) and regarding tooth loss and ACM (Supplementary Table 2).

### Risk of Bias Assessment

To estimate the potential risk of bias, the methodological qualities of the included studies were assessed. Overall, out of the 44 studies, the potential risk of bias was estimated to be “low” for 28 studies, “moderate” for three studies, and “serious” for 13 studies. This overall risk of bias assessment was estimated based on the risk of bias assessment within the seven domains. Within the domain confounding, there were 11 studies estimated as “serious,” and two studies were estimated as “moderate” risk of bias. Within the domain classification of exposures, there was only one study that was estimated as “moderate” risk of bias; within the domain measurement of the outcomes, there was only one study that was estimated as “serious” risk of bias; and within the domain missing data, two studies were estimated as “serious” risk of bias and two studies as “moderate” risk of bias. All the other studies within these domains were assessed as “low” risk of bias. Within the domain selection of participants into the study, departures from intended exposures, and selection of the reported result, all studies were estimated as “low” risk of bias (Supplementary File 5).

### Meta-Analyses for Tooth Loss and Atherosclerotic Cardiovascular Disease-Related Events

#### Cumulative Incidence of ACVD-Related Events, Categorical Data

**Figure 2:**
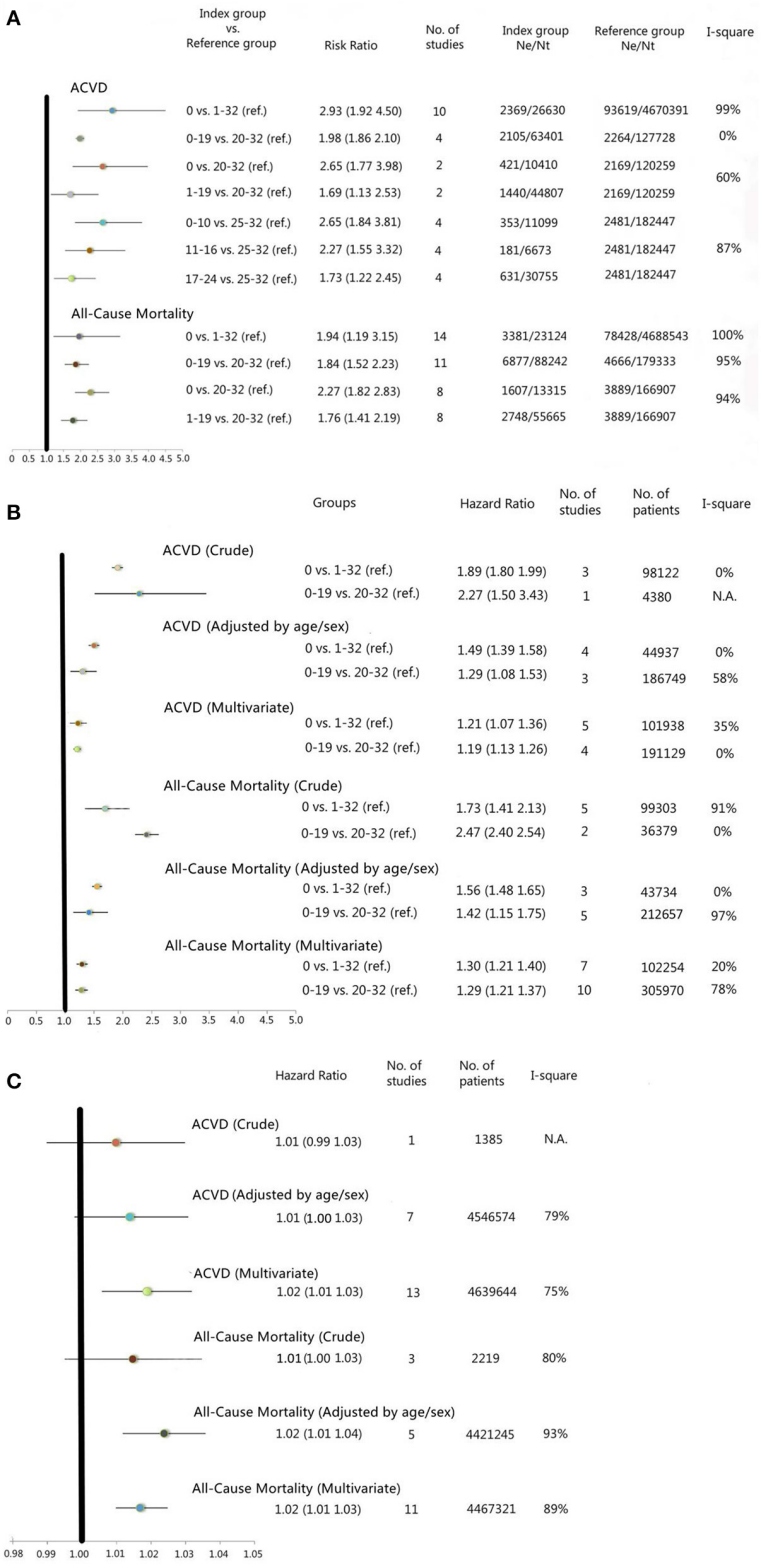
**(A)** Forest plots for meta-analysis of cumulative incidence for categorical data of the number of teeth, ACVD, and all-cause mortality. **(B)** Forest plots for meta-analysis of incidence density for categorical data of the number of teeth, atherosclerotic cardiovascular disease (ACVD) and all-cause mortality. **(C)** Forest plots for meta-analysis of incidence density for continuous data of the number of teeth, ACVD, and all-cause mortality. ACVD, Atherosclerotic Cardiovascular Disease; vs., versus; ref., reference group; Ne, number of events; Nt, number of total included study participants; N.A., Not Applicable. The Hazard Ratio (HR) in the Forest Plot which was based only on one study, was obtained directly from the included study.

Using pairwise meta-analysis, 10 studies were pooled, including the number of remaining teeth classified into 0 and 1–32 teeth, and four studies with the number of remaining teeth were classified into 0–19 and 20–32 teeth. Using network meta-analysis, two studies with the number of remaining teeth were classified into 0, 1–19, and 20–32 teeth, and four studies with the number of teeth were classified into 0–10, 11–16, 17–24, and 25–32 teeth. The results showed that the categories with a lower number of remaining teeth always had a significantly higher risk of ACVD-related events than the categories with a higher number of remaining teeth (Figure 2A and Supplementary Table 3). The highest RR was found in the comparison of 0 teeth vs. 1–32 remaining teeth [RR: 2.93 (1.92–4.50)], 0 teeth vs. 20–32 remaining teeth [RR: 2.65 (1.77–3.98)], and 0–10 remaining teeth vs. 25–32 remaining teeth [RR: 2.65 (1.84–3.81)] (Figure 2A and Supplementary Table 3). The heterogeneity of those meta-analyses ranged from 0 to 99%.

#### Incidence Density of ACVD-Related Events, Categorical Data

Using generic inverse variance meta-analysis for calculation of HR, five studies were included with the number of remaining teeth classified into 0 and 1–32 teeth, and four studies were included with the number of remaining teeth classified into 0–19 and 20–32 teeth. The results showed that the categories with a lower number of remaining teeth had significantly higher HR of ACVD-related events than the categories with a higher number of remaining teeth in both the crude and adjusted (for age/sex and for multiple variables) models (Figure 2B and Supplementary Table 4). The categories edentulous vs. 1–32 teeth and 0–19 teeth vs. 20–32, showed an increased hazard for ACVD in the lowest category of teeth [multiple adjusted model; 0 vs. 1–32, HR: 1.21 (1.07–1.36), 0–19 vs. 20–32, HR: 1.19 (1.13–1.26)] (Figure 2B and Supplementary Table 4). The heterogeneity of those meta-analyses ranged from 0 to 58%.

#### Incidence Density of ACVD-Related Events, Continuous Data

Using generic inverse variance meta-analysis for calculation of HR, 13 studies were included with the number of remaining teeth regarded as a continuous variable. The results showed an increased hazard for ACVD with an increased number of lost teeth in the multiple adjusted model [HR: 1.02 (1.01–1.03)] (Figure 2C and Supplementary Table 4). The heterogeneity of those meta-analyses ranged from 75 to 79%.

Most of the subgroup analyses, based on the approaches to count the remaining number of teeth, patients' ACVD status at baseline, and follow-up rate of the included studies, showed no statistically significant changes in the outcome (Supplementary Tables 3, 4). The heterogeneity was not reduced significantly in the subgroup analyses. This indicated that the three indicators may not significantly influence the outcomes and may not be the important reason to cause heterogeneity of the pooled results.

### Meta-Analyses for Tooth Loss and All-Cause Mortality

#### Cumulative Incidence of ACM, Categorical Data

Using pairwise meta-analysis, 14 studies were included with the number of remaining teeth classified into 0 and 1–32 teeth, and 11 studies with the number of remaining teeth classified into 0–19 and 20–32 teeth. Using network meta-analysis, eight studies were included with the number of remaining teeth classified into 0, 1–19, and 20–32 teeth. None classified the number of remaining teeth into four categories. The results showed that the categories with a lower number of remaining teeth had a significantly higher risk of ACM than the categories with a higher number of remaining teeth (Figure 2A and Supplementary Table 3). The highest RR was found in the comparison of 0 teeth vs. 20–32 remaining teeth [RR: 2.27 (1.82–2.83)] (Figure 2A and Supplementary Table 3). The heterogeneity of those meta-analyses ranged from 94 to 100%.

#### Incidence Density of ACM, Categorical Data

Using generic inverse variance meta-analysis, seven studies were included with the number of remaining teeth classified into 0 and 1–32 teeth, and 10 studies were included with the number of remaining teeth classified into 0–19 and 20–32 teeth. The results showed that the categories with a lower number of remaining teeth had significantly higher HR of ACM than the categories with a higher number of remaining teeth in both the crude and adjusted (for age/sex and for multiple variables) models (Figure 2B and Supplementary Table 4). The categories edentulous vs. 1–32 teeth and 0–19 teeth vs. 20–32 showed an increased hazard for ACM in the lowest category of teeth [multiple adjusted model; 0 vs. 1–32, HR: 1.30 (1.21–1.40), 0–19 vs. 20–32, HR: 1.29 (1.21–1.37)] (Figure 2B and Supplementary Table 4). The heterogeneity of those meta-analyses ranged from 0 to 97%.

#### Incidence Density of ACM, Continuous Data

Using generic inverse variance meta-analysis, 11 studies were included with the number of remaining teeth regarded as a continuous variable. The results showed an increased hazard for ACM with an increased number of lost teeth in the multiple adjusted model [HR: 1.02 (1.01–1.03)] (Figure 2C and Supplementary Table 4). The heterogeneity of those meta-analyses ranged from 80 to 93%.

Most of the subgroup analyses, based on the approaches to count the remaining number of teeth, patients' ACVD status at baseline, and follow-up rate of the included studies, showed no statistically significant changes in the outcome (Supplementary Tables 3, 4). The heterogeneity was not reduced significantly in all subgroup analyses, except in the subgroup analysis for crude HR of 0 vs. 1–32 remaining teeth. This indicated that the three indicators may not significantly influence the outcomes and may not be the important reason to cause heterogeneity of the pooled results.

The cumulative meta-analyses showed a rather limited overtime drift for the pooled RR and pooled HR and their confidence intervals (Supplementary Tables 5, 6). With some exemptions, the pooled RR and HR ranged between 1 and 2. Hence, there is a fairly stable overtime relationship between the number of teeth and ACVD or ACM-related outcomes, but the width of the confidence intervals did not narrow in a constant manner.

To estimate the level of publication bias, funnel plots were created for six meta-analyses with at least 10 studies. The results showed that there was no to minor publication bias for these meta-analyses (Supplementary File 6).

Supplementary File 7 presents a summary of the various factors used to rate the strength of the evidence according to GRADE. The rating was assessed for all the separate meta-analyses. The cumulative incidence for categorical data of the number of teeth and ACVD showed two meta-analyses having moderate strength of evidence and two meta-analyses having high strength of evidence. The cumulative incidence for categorical data of the number of teeth and ACM showed two meta-analyses having low strength of evidence and one meta-analysis having high strength of evidence. The multivariable incidence density for categorical and continuous data of the number of teeth and ACVD showed one meta-analysis having low strength of evidence and two meta-analyses having moderate strength of evidence. The multivariable incidence density for categorical and continuous data of the number of teeth and ACM showed two meta-analyses having low strength of evidence and one meta-analysis having moderate strength of evidence (Supplementary File 7).

## Discussion

To our knowledge, this systematic review and meta-analyses containing 75 prospective cohort studies from all over the world, including a diversity of populations of both men and women of all ages, is the largest to date providing evidence that tooth loss is related to an increased risk for ACVD-related events and ACM. The crude analyses showed that tooth loss is related to ACVD morbidity, ACVD mortality, and ACM: the lower the number of remaining teeth, the higher the risk of an ACVD event or death. These effects remained after adjusting for methodological weaknesses of the analyzed studies.

To interpret and explain the risk of tooth loss for ACVD, several plausible (biological) mechanisms need to be taken into consideration.

- Tooth loss and ACVD share risk factors, such as age, sex, SEP, obesity, and smoking (105). As a consequence, their subsequent occurrence during the life course reveals to be associated. While earlier research has shown a dose-response relationship (20), the direction, size, and precision of association estimates we reported here are consistent throughout all analyses. Hence, it is unlikely that this association is coincidental.
- Tooth loss is the ultimate event representing two major dental pathologies. (i) Dental caries is a lifelong disease and traditionally considered an important cause of tooth loss. Dental caries has a multifactorial etiology, but the consumption of dietary carbohydrates is the main factor (1, 2). This corresponds to a risk factor for ACVD, namely, overconsumption of carbohydrates leads to overweight and obesity, metabolic syndrome, and diabetes; these are obvious risk factors for ACVD (3–6). (ii) At older ages, periodontitis is the main cause of tooth loss. Periodontitis is a chronic multi-causal inflammatory disease of the supportive tissues of the teeth with progressive loss of attachment and alveolar bone, finally leading to tooth loss (1, 2). Ample research has been performed to identify pathophysiological mechanisms to explain the association between periodontitis and ACVD (11, 12). Thus, since periodontitis is associated with ACVD, the ultimate endpoint is also associated. A low-grade systemic inflammation in periodontitis and daily short-lived bacteremias in relation to atherosclerosis have been investigated (106–108). Low-grade chronic systemic inflammatory stress in periodontitis is considered to contribute to increased inflammation around atherosclerotic plaques at predilection places and vulnerable arteries; circulating bacteremia contribute to a pro-inflammatory and pro-thrombotic state, may induce autoimmunity, as well as dyslipidemia (11, 12). Increased adjusted RR for the relationship between periodontitis and ACVD from 1.50 up to 3.20 has been reported (14, 109).
- Masticatory dysfunction and related dietary changes have been proposed (5, 110, 111). An impaired masticatory function may lead to inadequate food choices and, therefore, reduced amounts of nutrients. This may include increases in intake of industrially processed rather than natural foods, avoiding hard-to-chew food, and home processing their foods into softer foods. In turn, this leads to increased intake of fermentable carbohydrates, saturated fatty acids, and reduction of the sources of dietary fiber and essential vitamins and minerals, consequently contributing to systemic diseases (3, 5, 110–113). A study reporting about the link between tooth loss, nutritional status, and stroke outcomes showed in the multivariable analysis that tooth loss and a worse nutritional status were independently associated with poor stroke outcomes (OR: 1.33) (114).
- Socioeconomic position (SEP) is an explanatory variable for a lower number of teeth for which strong and consistent evidence is available. SEP is defined mainly based on income, education, and employment status. Several studies show that low SEP is associated with significant tooth loss (7, 8). SEP influences lifestyle habits like smoking and performing good oral hygiene. Also, access to healthcare centers and periodic dental examination is more limited for people with a low SEP (7, 115, 116). SEP also plays a role in the development of ACVD. In people with low SEP, biological, behavioral, material, and psychosocial risk factors (like health insurance and financial difficulties, obesity, smoking, physical inactivity, life events, educational inequalities), lower health literacy, and inequalities in access to care and medical treatment, accentuate the link between SEP, ACVD, and mortality (8, 117). For low adulthood SEP, multiple adjusted increased risks for ACVD up to 1.84 were reported (8).

Despite a large number of studies and the state-of-art methodologies applied, within the findings of this systematic review and meta-analyses, the following aspects observed in the study methods need attention.

- The risk of bias assessment in the individual studies, by using the ROBINS-E tool, showed a low risk of bias for 28 studies, a moderate risk of bias for 3 studies, and a serious risk of bias for 13 studies. For the studies with moderate and serious risk of bias, the main issue was the bias due to confounding, where important covariables for the relation between the number of teeth and ACVD or ACM were not available in the multivariable analyses. It is difficult to predict the direction of bias for the outcome, but we can assume that a suboptimal multivariable analysis causes an overestimation of the effect. Funnel plots were made to assess the publication bias for the larger meta-analyses and showed none to a minor level of publication bias.
- Some studies defined the determinant (number of remaining or missing teeth) based on patients' self-reported information. This may be less accurate than the number of teeth based on clinical examination and may bias the results of the review. In addition, most included studies use categories containing up to 32 remaining teeth. Whether third molars should be incorporated in defining a full dentition remains controversial. This aspect could introduce bias, but considering the fact that the used categories contain groups of 1–32, 20–32, or 25–32 teeth, the introduced bias is negligible considering a full dentition containing only 28 teeth (excluding third molars) is also incorporated in those categories. Also, a large number of the currently included studies used two groups of present teeth in their statistical analysis: groups 0–19 teeth vs. 20–32 teeth. Most likely, this is based on the fact that in dentistry, 20 teeth present is considered as the minimal number of teeth needed for sufficient chewing ability (118).
- Only 15 studies included in the meta-analyses are inception cohort studies, which only included participants without ACVD at baseline. In another 14 studies, a part of the participants had ACVD at baseline, but this was adjusted for in the analysis. However, in the remaining 15 studies, some participants had ACVD at baseline without adjustment in the analysis or the information on participants' ACVD status at baseline, and the adjustment was not reported. For those 15 studies, the association between the number of teeth and the outcomes may be overestimated if the prevalent ACVD at baseline was included without adjustment. This may bias the pooled results of the meta-analyses.
- The follow-up rate differed between studies and was classified into <80% and ≥80% follow-up rates. Loss to follow-up may severely compromise the validity of a study and bias the results if the patients who drop out are different from those who do not drop out (119). Based on the rule of thumb, a dropout rate >20% may cause serious bias (120). In the present study, we assumed that the patients who experienced the events at follow-up may be more likely to drop out than those without events, and this may lead to the underestimation of the results. However, the subgroup analyses using the three indicators for risk of bias, i.e., the approaches to count the number of remaining teeth, patients' ACVD status at baseline, and follow-up rate, showed a very minor effect on the heterogeneity of the meta-analyses. This indicated that the level of risk of bias across the included studies was not the main reason for the heterogeneity of the meta-analyses (Supplementary Tables 3, 4).
- The harmonization of the number of teeth varied across studies. Some studies reported the number of remaining or missing teeth as a continuous variable, while other studies used different categories for the number of remaining or missing teeth, and these categories were not always comparable and transferrable across included studies. Also, probably in most of these studies, the categories were *post-hoc* defined. Therefore, 44 studies using the four predefined common categories of exposure comparisons qualified for meta-analyses. Due to the exclusion of 31 studies, an overestimation of effect cannot completely be ruled out. However, by using this predefined approach to meta-analysis, we have found a consistent effect across the reported separate meta-analyses. Moreover, none of the overall estimates of the cumulative meta-analyses showed meaningful changes over time (Supplementary Tables 5, 6).
- The heterogeneity in the meta-analyses was high. The *I*^2^ for some of the meta-analyses was larger than 75% (Figures 2A–C and Supplementary Tables 3, 4), which indicated considerable heterogeneity. So perhaps, conventionally, one would refrain from pooling, as a random-effects model will not allow remediation of this situation (27). Moreover, the sensitivity analyses did not provide clues for sources of heterogeneity. Therefore, the remaining uncertainty is whether this unexplained heterogeneity due to the residual variation results in either an overestimation or an underestimation of the estimates of effect (so bias in the estimates). The high heterogeneity may be caused by differences in follow-up periods and multivariate adjustments across included studies, but also by their variated study populations, notably patients' age and countries of origin. For example, there were quite some studies only including participants at older ages (≥60 years of age) who are known, on the one hand, to carry a higher risk for ACVD events or ACM (121), while the peak incidence of severe tooth loss is at 65 years of age (4). The follow-up time of the included studies was quite diverse. With a longer follow-up period, more study participants may experience an ACVD event or die. Also, the exposure, tooth loss, is a time-dependent variable, while in most studies, it is reported as the number of teeth at baseline, which is seen as time independent. This may have resulted in an underestimation of the effect. In addition, the covariates in the multivariate models are diverse across studies. The multivariate HR values adjusted for various covariates were directly pooled in the meta-analysis. Besides, the rationale for selection of the covariates in most studies was not reported. It is not known whether the covariates were determined *a priori* or simply based on the availability of potential confounding data at hand. If the latter is the case, this may have biased the findings of the meta-analyses.
- Across studies, the evidence emerging from the separate meta-analysis, assessed by the use of GRADE, varies. For the meta-analyses with ACVD as the outcome, the strength of the evidence was mainly moderate. For the meta-analyses with ACM as the outcome, the strength of the evidence was mainly low. Therefore, our conclusion based on the outcome ACVD is more valid than our conclusion based on the outcome ACM.

In conclusion, this large systematic review and meta-analysis of survival data shows that a lower number of teeth increases the risk of ACVD-related morbidity or mortality, and ACM. Dental professionals should use this knowledge to inform their patients and the public at large to be aware of their general health and visit their general physician to discuss this aspect; and, vice versa, medical specialists should motivate their patients to visit the dentist regularly and to encourage them to maintain their own teeth as much as possible.

## Data Availability Statement

The original contributions presented in the study are included in the article/Supplementary Material, further inquiries can be directed to the corresponding author/s.

## Author Contributions

NB and NS contributed to the conception, design, data acquisition, analysis, interpretation, and drafted and critically revised the manuscript. BL and GH contributed to the conception, design, analysis, interpretation, and critically revised the manuscript. All authors gave final approval and agree to be accountable for all aspects of the work ensuring integrity and accuracy.

## Conflict of Interest

The authors declare that the research was conducted in the absence of any commercial or financial relationships that could be construed as a potential conflict of interest.
