# Supplementary material for: Lower Number of Teeth Is Related to Higher Risks for ACVD and Death—Systematic Review and Meta-Analyses of Survival Data
Source: Front Cardiovasc Med. 2021 May 7;8:621626. doi: 10.3389/fcvm.2021.621626 (PMC8138430; doi:10.3389/fcvm.2021.621626)
Supplement: Supplementary file 3 [file Data_Sheet_3.doc]

Supplementary File 3: Search Strategy for PubMed-MEDLINE and Embase

**PubMed MEDLINE**

**#1 Tooth loss**

"Tooth Loss"[Mesh] OR "Mouth, Edentulous"[Mesh] OR Tooth Loss*[tiab] OR edentul*[tiab] OR toothless[tiab] OR dental loss*[tiab] OR missing teeth[tiab] OR "number of teeth"[tiab] OR "number teeth"[tiab] OR "losing teeth"[tiab] OR "losing a tooth"[tiab]

**#2 Cardiovascular diseases**

"Cardiovascular Diseases"[Mesh] OR "Hyperlipidemias"[Mesh] OR "Cholesterol"[Mesh] OR "Stroke"[Mesh] OR "Chronic Disease"[Mesh] OR "Death"[Mesh:NoExp] OR "Mortality"[Mesh] OR cardiovascular*[tiab] OR cvd[tiab] OR coronar*[tiab] OR heart*[tiab] OR atherosclero*[tiab] OR arteriosclero*[tiab] OR hypertension[tiab] OR blood pressure[tiab] OR hyperlipidaemia*[tiab] OR hyperlipidemia*[tiab] OR cholesterol*[tiab] OR stroke*[tiab] OR peripheral vascular[tiab] OR peripheral arterial[tiab] OR hypercholesterol*[tiab] OR hyperlipid*[tiab] OR ischemi*[tiab] OR ischaemi*[tiab] OR angina*[tiab] OR coronar*[tiab] OR myocard*[tiab] OR infarcti*[tiab] OR cerebrovascul*[tiab] OR cva[tiab] OR cvas[tiab] OR stroke[tiab] OR cerebrovascular[tiab] OR apoplex*[tiab] OR "brain vascular accident"[tiab] OR "brain vascular accidents"[tiab] OR systemic disease*[tiab] OR chronic disease*[tiab] OR death*[tiab] OR dying[tiab] OR mortalit*[tiab]

**EMBASE**

**#1 Tooth loss**

'edentulousness'/exp OR 'Tooth Loss*':ti,ab OR edentul*:ti,ab OR toothless:ti,ab OR 'dental loss*':ti,ab OR 'missing teeth':ti,ab OR 'number of teeth':ti,ab OR 'number teeth':ti,ab OR 'losing teeth':ti,ab OR 'losing a tooth':ti,ab

**#2 Cardiovascular diseases**

'cardiovascular disease'/exp OR 'hyperlipidemia'/exp OR 'cholesterol'/exp OR 'cerebrovascular accident'/exp OR 'chronic disease'/exp OR 'death'/exp OR 'mortality'/exp OR cardiovascular*:ti,ab OR cvd:ti,ab OR coronar*:ti,ab OR heart*:ti,ab OR atherosclero*:ti,ab OR arteriosclero*:ti,ab OR hypertension:ti,ab OR 'blood pressure':ti,ab OR hyperlipidaemia*:ti,ab OR hyperlipidemia*:ti,ab OR cholesterol*:ti,ab OR stroke*:ti,ab OR 'peripheral vascular':ti,ab OR 'peripheral arterial':ti,ab OR hypercholesterol*:ti,ab OR hyperlipid*:ti,ab OR ischemi*:ti,ab OR ischaemi*:ti,ab OR angina*:ti,ab OR coronar*:ti,ab OR myocard*:ti,ab OR infarcti*:ti,ab OR cerebrovascul*:ti,ab OR cva:ti,ab OR cvas:ti,ab OR stroke:ti,ab OR cerebrovascular:ti,ab OR apoplex*:ti,ab OR 'brain vascular accident':ti,ab OR 'brain vascular accidents':ti,ab OR 'systemic disease*':ti,ab OR 'chronic disease*':ti,ab OR death*:ti,ab OR dying:ti,ab OR mortalit*:ti,ab
