# Supplementary material for: Lower Number of Teeth Is Related to Higher Risks for ACVD and Death—Systematic Review and Meta-Analyses of Survival Data
Source: Front Cardiovasc Med. 2021 May 7;8:621626. doi: 10.3389/fcvm.2021.621626 (PMC8138430; doi:10.3389/fcvm.2021.621626)
Supplement: Supplementary file 4 [file Data_Sheet_4.docx]

**Reference list systematic review:**

1. Abnet CC, Qiao YL, Dawsey SM, Dong ZW, Taylor PR, Mark SD. Tooth loss is associated with increased risk of total death and death from upper gastrointestinal cancer, heart disease, and stroke in a Chinese population-based cohort. Int J Epidemiol. 2005;34(2):467-74.

2. Adolph M, Darnaud C, Thomas F, Pannier B, Danchin N, Batty GD, et al. Oral health in relation to all-cause mortality: the IPC cohort study. Sci Rep. 2017;7:44604.

3. Aida J, Kondo K, Yamamoto T, Hirai H, Nakade M, Osaka K, et al. Oral health and cancer, cardiovascular, and respiratory mortality of Japanese. Journal of dental research. 2011;90(9):1129-35.

4. Ajwani S, Mattila KJ, Narhi TO, Tilvis RS, Ainamo A. Oral health status, C-reactive protein and mortality--a 10 year follow-up study. Gerodontology. 2003;20(1):32-40.

5. Ajwani S, Mattila KJ, Tilvis RS, Ainamo A. Periodontal disease and mortality in an aged population. Spec Care Dentist. 2003;23(4):125-30.

6. Ando A, Tanno K, Ohsawa M, Onoda T, Sakata K, Tanaka F, et al. Associations of number of teeth with risks for all-cause mortality and cause-specific mortality in middle-aged and elderly men in the northern part of Japan: the Iwate-KENCO study. Community Dent Oral Epidemiol. 2014;42(4):358-65.

7. Ansai T, Takata Y, Soh I, Awano S, Yoshida A, Sonoki K, et al. Relationship between tooth loss and mortality in 80-year-old Japanese community-dwelling subjects. BMC Public Health. 2010;10:386.

8. Batty GD, Jung KJ, Mok Y, Lee SJ, Back JH, Lee S, et al. Oral health and later coronary heart disease: Cohort study of one million people. Eur J Prev Cardiol. 2018;25(6):598-605.

9. Brown DW. Complete edentulism prior to the age of 65 years is associated with all-cause mortality. Journal of public health dentistry. 2009;69(4):260-6.

10. Cabrera C, Hakeberg M, Ahlqwist M, Wedel H, Bjorkelund C, Bengtsson C, et al. Can the relation between tooth loss and chronic disease be explained by socio-economic status? A 24-year follow-up from the population study of women in Gothenburg, Sweden. Eur J Epidemiol. 2005;20(3):229-36.

11. Caplan DJ, Ghazal TS, Cowen HJ, Oliveira DC. Dental status as a predictor of mortality among nursing facility residents in eastern Iowa. Gerodontology. 2017;34(2):257-63.

12. Chang Y, Woo HG, Lee JS, Song TJ. Better oral hygiene is associated with lower risk of stroke. Journal of periodontology. 2020.

13. Chang Y, Woo HG, Park J, Lee JS, Song TJ. Improved oral hygiene care is associated with decreased risk of occurrence for atrial fibrillation and heart failure: A nationwide population-based cohort study. Eur J Prev Cardiol. 2019:2047487319886018.

14. Choe H, Kim YH, Park JW, Kim SY, Lee SY, Jee SH. Tooth loss, hypertension and risk for stroke in a Korean population. Atherosclerosis. 2009;203(2):550-6.

15. Darnaud C, Thomas F, Danchin N, Boutouyrie P, Bouchard P. Masticatory Capacity and Mortality: The Preventive and Clinical Investigation Center (IPC) Cohort Study. Journal of dental research. 2020;99(2):152-8.

16. Del Brutto OH, Mera RM, Zambrano M, Del Brutto VJ. Severe edentulism is a major risk factor influencing stroke incidence in rural Ecuador (The Atahualpa Project). Int J Stroke. 2017;12(2):201-4.

17. Dewake N, Hashimoto H, Nonoyama T, Nonoyama K, Shimazaki Y. Posterior occluding pairs of teeth or dentures and 1-year mortality in nursing home residents in Japan. J Oral Rehabil. 2020;47(2):204-11.

18. Dietrich T, Jimenez M, Krall Kaye EA, Vokonas PS, Garcia RI. Age-dependent associations between chronic periodontitis/edentulism and risk of coronary heart disease. Circulation. 2008;117(13):1668-74.

19. Fukai K, Takiguchi T, Ando Y, Aoyama H, Miyakawa Y, Ito G, et al. Mortality rates of community-residing adults with and without dentures. Geriatr Gerontol Int. 2008;8(3):152-9.

20. Furuta M, Takeuchi K, Adachi M, Kinoshita T, Eshima N, Akifusa S, et al. Tooth loss, swallowing dysfunction and mortality in Japanese older adults receiving home care services. Geriatr Gerontol Int. 2018;18(6):873-80.

21. Garcia RI, Krall EA, Vokonas PS. Periodontal disease and mortality from all causes in the VA Dental Longitudinal Study. Annals of periodontology / the American Academy of Periodontology. 1998;3(1):339-49.

22. Goto Y, Wada K, Uji T, Koda S, Mizuta F, Yamakawa M, et al. Number of Teeth and All-Cause and Cancer Mortality in a Japanese Community: The Takayama Study. J Epidemiol. 2020;30(5):213-8.

23. Hamalainen P, Meurman JH, Keskinen M, Heikkinen E. Relationship between dental health and 10-year mortality in a cohort of community-dwelling elderly people. Eur J Oral Sci. 2003;111(4):291-6.

24. Hayasaka K, Tomata Y, Aida J, Watanabe T, Kakizaki M, Tsuji I. Tooth loss and mortality in elderly Japanese adults: effect of oral care. J Am Geriatr Soc. 2013;61(5):815-20.

25. Heitmann BL, Gamborg M. Remaining teeth, cardiovascular morbidity and death among adult Danes. Prev Med. 2008;47(2):156-60.

26. Hiratsuka T, Komiyama T, Ohi T, Tanji F, Tomata Y, Tsuji I, et al. Contribution of systemic inflammation and nutritional status to the relationship between tooth loss and mortality in a community-dwelling older Japanese population: a mediation analysis of data from the Tsurugaya project. Clin Oral Investig. 2020;24(6):2071-7.

27. Hirotomi T, Yoshihara A, Ogawa H, Miyazaki H. Number of teeth and 5-year mortality in an elderly population. Community Dent Oral Epidemiol. 2015;43(3):226-31.

28. Hoke M, Schillinger T, Mlekusch W, Wagner O, Minar E, Schillinger M. The impact of dental disease on mortality in patients with asymptomatic carotid atherosclerosis. Swiss Med Wkly. 2011;141:w13236.

29. Holmlund A, Holm G, Lind L. Number of teeth as a predictor of cardiovascular mortality in a cohort of 7,674 subjects followed for 12 years. Journal of periodontology. 2010;81(6):870-6.

30. Holmlund A, Lampa E, Lind L. Oral health and cardiovascular disease risk in a cohort of periodontitis patients. Atherosclerosis. 2017;262:101-6.

31. Holm-Pedersen P, Schultz-Larsen K, Christiansen N, Avlund K. Tooth loss and subsequent disability and mortality in old age. J Am Geriatr Soc. 2008;56(3):429-35.

32. Hu HY, Lee YL, Lin SY, Chou YC, Chung D, Huang N, et al. Association Between Tooth Loss, Body Mass Index, and All-Cause Mortality Among Elderly Patients in Taiwan. Medicine (Baltimore). 2015;94(39):e1543.

33. Hung HC, Joshipura KJ, Colditz G, Manson JE, Rimm EB, Speizer FE, et al. The association between tooth loss and coronary heart disease in men and women. Journal of public health dentistry. 2004;64(4):209-15.

34. Hung HC, Willett W, Merchant A, Rosner BA, Ascherio A, Joshipura KJ. Oral health and peripheral arterial disease. Circulation. 2003;107(8):1152-7.

35. Iwasaki M, Sato M, Yoshihara A, Ansai T, Miyazaki H. Association between tooth loss and medical costs related to stroke in healthy older adults aged over 75 years in Japan. Geriatr Gerontol Int. 2017;17(2):202-10.

36. Janket SJ, Baird AE, Jones JA, Jackson EA, Surakka M, Tao W, et al. Number of teeth, C-reactive protein, fibrinogen and cardiovascular mortality: a 15-year follow-up study in a Finnish cohort. Journal of clinical periodontology. 2014;41(2):131-40.

37. Janket SJ, Surakka M, Jones JA, Lam A, Schnell RA, Rose LM, et al. Removable dental prostheses and cardiovascular survival: a 15-year follow-up study. J Dent. 2013;41(8):740-6.

38. Joshipura KJ, Hung HC, Rimm EB, Willett WC, Ascherio A. Periodontal disease, tooth loss, and incidence of ischemic stroke. Stroke; a journal of cerebral circulation. 2003;34(1):47-52.

39. Joshipura KJ, Rimm EB, Douglass CW, Trichopoulos D, Ascherio A, Willett WC. Poor oral health and coronary heart disease. Journal of dental research. 1996;75(9):1631-6.

40. Joshy G, Arora M, Korda RJ, Chalmers J, Banks E. Is poor oral health a risk marker for incident cardiovascular disease hospitalisation and all-cause mortality? Findings from 172 630 participants from the prospective 45 and Up Study. BMJ Open. 2016;6(8):e012386.

41. Kebede TG, Holtfreter B, Kocher T, Meisel P, Dietrich T, Biffar R, et al. Association of Periodontal Destruction and Diabetes with Mortality. Journal of dental research. 2017;96(1):56-63.

42. Kim JK, Baker LA, Davarian S, Crimmins E. Oral health problems and mortality. J Dent Sci. 2013;8(2).

43. LaMonte MJ, Genco RJ, Hovey KM, Wallace RB, Freudenheim JL, Michaud DS, et al. History of Periodontitis Diagnosis and Edentulism as Predictors of Cardiovascular Disease, Stroke, and Mortality in Postmenopausal Women. J Am Heart Assoc. 2017;6(4).

44. Lee HJ, Choi EK, Park JB, Han KD, Oh S. Tooth Loss Predicts Myocardial Infarction, Heart Failure, Stroke, and Death. Journal of dental research. 2019;98(2):164-70.

45. Li Q, Chalmers J, Czernichow S, Neal B, Taylor BA, Zoungas S, et al. Oral disease and s ubsequent cardiovascular disease in people with type 2 diabetes: a prospective cohort study based on the Action in Diabetes and Vascular Disease: Preterax and Diamicron Modified-Release Controlled Evaluation (ADVANCE) trial. Diabetologia. 2010;53(11):2320-7.

46. Liljestrand JM, Havulinna AS, Paju S, Mannisto S, Salomaa V, Pussinen PJ. Missing Teeth Predict Incident Cardiovascular Events, Diabetes, and Death. Journal of dental research. 2015;94(8):1055-62.

47. Matsuyama Y, Aida J, Watt RG, Tsuboya T, Koyama S, Sato Y, et al. Dental Status and Compression of Life Expectancy with Disability. Journal of dental research. 2017;96(9):1006-13.

48. Morita I, Nakagaki H, Kato K, Murakami T, Tsuboi S, Hayashizaki J, et al. Relationship between survival rates and numbers of natural teeth in an elderly Japanese population. Gerodontology. 2006;23(4):214-8.

49. Morrison HI, Ellison LF, Taylor GW. Periodontal disease and risk of fatal coronary heart and cerebrovascular diseases. J Cardiovasc Risk. 1999;6(1):7-11.

50. Mucci LA, Hsieh CC, Williams PL, Arora M, Adami HO, de Faire U, et al. Do genetic factors explain the association between poor oral health and cardiovascular disease? A prospective study among Swedish twins. American journal of epidemiology. 2009;170(5):615-21.

51. Munoz-Torres FJ, Mukamal KJ, Pai JK, Willett W, Joshipura KJ. Relationship between tooth loss and peripheral arterial disease among women. Journal of clinical periodontology. 2017;44(10):989-95.

52. Noguchi S, Toyokawa S, Miyoshi Y, Suyama Y, Inoue K, Kobayashi Y. Five-year follow-up study of the association between periodontal disease and myocardial infarction among Japanese male workers: MY Health Up Study. J Public Health (Oxf). 2015;37(4):605-11.

53. Nomura Y, Kakuta E, Okada A, Otsuka R, Shimada M, Tomizawa Y, et al. Effects of self-assessed chewing ability, tooth loss and serum albumin on mortality in 80-year-old individuals: a 20-year follow-up study. BMC Oral Health. 2020;20(1):122.

54. Oluwagbemigun K, Dietrich T, Pischon N, Bergmann M, Boeing H. Association between Number of Teeth and Chronic Systemic Diseases: A Cohort Study Followed for 13 Years. PLoS One. 2015;10(5):e0123879.

55. Osterberg T, Carlsson GE, Sundh V, Mellstrom D. Number of teeth--a predictor of mortality in 70-year-old subjects. Community Dent Oral Epidemiol. 2008;36(3):258-68.

56. Osterberg T, Carlsson GE, Sundh V, Steen B. Number of teeth--a predictor of mortality in the elderly? A population study in three Nordic localities. Acta odontologica Scandinavica. 2007;65(6):335-40.

57. Padilha DM, Hilgert JB, Hugo FN, Bos AJ, Ferrucci L. Number of teeth and mortality risk in the Baltimore Longitudinal Study of Aging. J Gerontol A Biol Sci Med Sci. 2008;63(7):739-44.

58. Paganini-Hill A, White SC, Atchison KA. Dental health behaviors, dentition, and mortality in the elderly: the leisure world cohort study. J Aging Res. 2011;2011:156061.

59. Park SY, Kim SH, Kang SH, Yoon CH, Lee HJ, Yun PY, et al. Improved oral hygiene care attenuates the cardiovascular risk of oral health disease: a population-based study from Korea. Eur Heart J. 2019;40(14):1138-45.

60. Qi L, Qian Y, Zhu F, Cao N, Lu H, Zhang L. Association between periodontal disease and tooth loss and mortality in an elderly Chinese population. Aging Clin Exp Res. 2020.

61. Ragnarsson E, Eliasson ST, Gudnason V. Loss of teeth and coronary heart disease. Int J Prosthodont. 2004;17(4):441-6.

62. Reichert S, Schlitt A, Beschow V, Lutze A, Lischewski S, Seifert T, et al. Use of floss/interdental brushes is associated with lower risk for new cardiovascular events among patients with coronary heart disease. J Periodontal Res. 2015;50(2):180-8.

63. Reichert S, Schulz S, Benten AC, Lutze A, Seifert T, Schlitt M, et al. Periodontal conditions and incidence of new cardiovascular events among patients with coronary vascular disease. Journal of clinical periodontology. 2016;43(11):918-25.

64. Saito M, Shimazaki Y, Nonoyama T, Tadokoro Y. Associations of number of teeth with medical costs and hospitalization duration in an older Japanese population. Geriatr Gerontol Int. 2019;19(4):335-41.

65. Schwahn C, Polzer I, Haring R, Dorr M, Wallaschofski H, Kocher T, et al. Missing, unreplaced teeth and risk of all-cause and cardiovascular mortality. Int J Cardiol. 2013;167(4):1430-7.

66. Shimazaki Y, Soh I, Saito T, Yamashita Y, Koga T, Miyazaki H, et al. Influence of dentition status on physical disability, mental impairment, and mortality in institutionalized elderly people. Journal of dental research. 2001;80(1):340-5.

67. Soikkonen K, Wolf J, Salo T, Tilvis R. Radiographic periodontal attachment loss as an indicator of death risk in the elderly. Journal of clinical periodontology. 2000;27(2):87-92.

68. Tu YK, Galobardes B, Smith GD, McCarron P, Jeffreys M, Gilthorpe MS. Associations between tooth loss and mortality patterns in the Glasgow Alumni Cohort. Heart. 2007;93(9):1098-103.

69. Tuominen R, Reunanen A, Paunio M, Paunio I, Aromaa A. Oral health indicators poorly predict coronary heart disease deaths. Journal of dental research. 2003;82(9):713-8.

70. Vedin O, Hagstrom E, Ostlund O, Avezum A, Budaj A, Flather MD, et al. Associations between tooth loss and prognostic biomarkers and the risk for cardiovascular events in patients with stable coronary heart disease. Int J Cardiol. 2017;245:271-6.

71. Vedin O, Hagstrom E, Budaj A, Denchev S, Harrington RA, Koenig W, et al. Tooth loss is independently associated with poor outcomes in stable coronary heart disease. Eur J Prev Cardiol. 2016;23(8):839-46.

72. Vogtmann E, Etemadi A, Kamangar F, Islami F, Roshandel G, Poustchi H, et al. Oral health and mortality in the Golestan Cohort Study. Int J Epidemiol. 2017;46(6):2028-35.

73. Watt RG, Tsakos G, de Oliveira C, Hamer M. Tooth loss and cardiovascular disease mortality risk--results from the Scottish Health Survey. PLoS One. 2012;7(2):e30797.

74. Wu T, Trevisan M, Genco RJ, Dorn JP, Falkner KL, Sempos CT. Periodontal disease and risk of cerebrovascular disease: the first national health and nutrition examination survey and its follow-up study. Arch Intern Med. 2000;160(18):2749-55.

75. Yuan JQ, Lv YB, Kraus VB, Gao X, Yin ZX, Chen HS, et al. Number of natural teeth, denture use and mortality in Chinese elderly: a population-based prospective cohort study. BMC Oral Health. 2020;20(1):100.
