# Supplementary material for: Lower Number of Teeth Is Related to Higher Risks for ACVD and Death—Systematic Review and Meta-Analyses of Survival Data
Source: Front Cardiovasc Med. 2021 May 7;8:621626. doi: 10.3389/fcvm.2021.621626 (PMC8138430; doi:10.3389/fcvm.2021.621626)
Supplement: Supplementary file 5 [file Data_Sheet_5.docx]

Supplementary File 5: Summary of risk of bias assessment of included studies based on ROBINS-E

| **Study** | **Domains of Risk of Bias** | | | | | | | **Overall Risk of Bias** |
| --- | --- | --- | --- | --- | --- | --- | --- | --- |
|  | **Confounding** | **Selection of participants** | **Classification of exposures** | **Departures from intended exposures** | **Missing data** | **Measurement of outcomes** | **Selection of the reported result** |  |
| Abnet ea. 2005 | ⚫ | - | - | - | - | - | - | ⚫ |
| Aida ea. 2011 | - | - | - | - | - | - | - | - |
| Ajwani ea. 2003 | - | - | - | - | ⚫ | - | - | ⚫ |
| Ando ea. 2014 | - | - | - | - | - | - | - | - |
| Brown ea. 2009 | - | - | - | - | - | - | - | - |
| Caplan ea. 2017 | ⭘ | - | - | - | ⭘ | - | - | ⭘ |
| Dewake ea. 2020 | - | - | - | - | - | - | - | - |
| Dietrich ea. 2008 | - | - | - | - | - | - | - | - |
| Garcia ea. 1998 | - | - | - | - | - | - | - | - |
| Goto ea. 2020 | - | - | - | - | - | - | - | - |
| Hamalainen ea. 2003 | - | - | - | - | - | - | - | - |
| Hayasaka ea. 2013 | - | - | - | - | - | - | - | - |
| Heitmann ea. 2008 | ⚫ | - | - | - | - | - | - | ⚫ |
| Hiratsuka ea. 2020 | - | - | - | - | - | - | - | - |
| Hoke ea. 2011 | - | - | - | - | - | - | - | - |
| Holm-Pedersen ea. 2008 | ⚫ | - | - | - | - | - | - | ⚫ |
| Hu ea. 2015 | - | - | - | - | - | - | - | - |
| Hung ea. 2003 | - | - | - | - | - | - | - | - |
| Hung ea. 2004 | - | - | - | - | - | - | - | - |
| Janket ea. 2014 | ⚫ | - | - | - | - | - | - | ⚫ |
| Joshipura ea. 1996 | - | - | - | - | - | - | - | - |
| Joshipura ea. 2003 | - | - | - | - | - | - | - | - |
| Joshy ea. 2016 | - | - | - | - | - | - | - | - |
| Kebede ea. 2017 | ⚫ | - | - | - | - | - | - | ⚫ |
| LaMonte ea. 2017 | - | - | - | - | - | - | - | - |
| Lee ea. 2019 | - | - | - | - | - | - | - | - |
| Morita ea. 2006 | ⚫ | - | - | - | - | - | - | ⚫ |
| Morrison ea. 1999 | - | - | - | - | NI | - | - | - |
| Nomura ea. 2020 | - | - | - | - | - | - | - | - |
| Oluwagbemigun ea. 2015 | - | - | - | - | - | - | - | - |
| Osterberg ea. 2007 | - | - | - | - | - | - | - | - |
| Osterberg ea. 2008 | - | - | - | - | ⭘ | - | - | ⭘ |
| Padilha ea. 2008 | - | - | ⭘ | - | - | - | - | ⭘ |
| Paganini-Hill ea. 2011 | - | - | - | - | - | - | - | - |
| Qi ea. 2020 | ⚫ | - | - | - | - | ⚫ | - | ⚫ |
| Ragnarsson ea. 2004 | ⚫ | - | - | - | - | - | - | ⚫ |
| Reichert ea. 2015 | ⚫ | - | - | - | - | - | - | ⚫ |
| Shimazaki ea. 2001 | ⭘ | - | - | - | ⚫ | - | - | ⚫ |
| Soikkonen ea. 2000 | - | - | - | - | - | - | - | - |
| Tu ea. 2007 | ⚫ | - | - | - | - | - | - | ⚫ |
| Vedin ea. 2017 | - | - | - | - | - | - | - | - |
| Watt ea. 2012 | ⚫ | - | - | - | - | - | - | ⚫ |
| Wu ea. 2000 | - | - | - | - | - | - | - | - |
| Yuan ea. 2020 | - | - | - | - | - | - | - | - |

Risk of Bias

- = Low

⭘ = Moderate

⚫ = Serious

NI = No Information
