# Supplementary material for: Lower Number of Teeth Is Related to Higher Risks for ACVD and Death—Systematic Review and Meta-Analyses of Survival Data
Source: Front Cardiovasc Med. 2021 May 7;8:621626. doi: 10.3389/fcvm.2021.621626 (PMC8138430; doi:10.3389/fcvm.2021.621626)
Supplement: Supplementary file 6 [file Data_Sheet_6.docx]

Supplementary File 6: Funnel plots of the meta-analysis

Funnel plot for meta-analysis with Risk Ratio between 0 teeth and 1-32 teeth for ACVD (n = 10)

Funnel plot for meta-analysis with Risk Ratio between 0 teeth and 1-32 teeth for All-Cause Mortality (n =14)

Funnel plot for meta-analysis with Risk Ratio between 0-19 teeth and 20-32 teeth for All-Cause Mortality (n = 11)

Funnel plot for meta-analysis with Multivariate Hazard Ratio between 0-19 teeth and 20-32 teeth for All-Cause Mortality (n = 10)

Funnel plot for meta-analysis with Multivariate Hazard Ratio for number of lost teeth for ACVD (n = 13)

Funnel plot for meta-analysis with Multivariate Hazard Ratio for number of lost teeth for All-Cause Mortality (n = 11)
