# Supplementary material for: Lower Number of Teeth Is Related to Higher Risks for ACVD and Death—Systematic Review and Meta-Analyses of Survival Data
Source: Front Cardiovasc Med. 2021 May 7;8:621626. doi: 10.3389/fcvm.2021.621626 (PMC8138430; doi:10.3389/fcvm.2021.621626)
Supplement: Supplementary file 7 [file Data_Sheet_7.docx]

Supplementary File 7: GRADE assessment

Cumulative incidence for categorical data of number of teeth and ACVD

|  | **Study design** | **Number of studies** | **Study limitations** | **Inconsistency** | **Indirectness** | **Imprecision** | **Publication Bias** | **Magnitude of the effect** | **Dose-response gradient** | **Effect of plausible confounding** | **Strength of the evidence** |
| --- | --- | --- | --- | --- | --- | --- | --- | --- | --- | --- | --- |
| 0 vs.  1-32 teeth (ref.) | Observational | 10 | Low | Serious inconsistency  I^2^ = 99% | None | None | Minor | Large | N.A. | None | Moderate  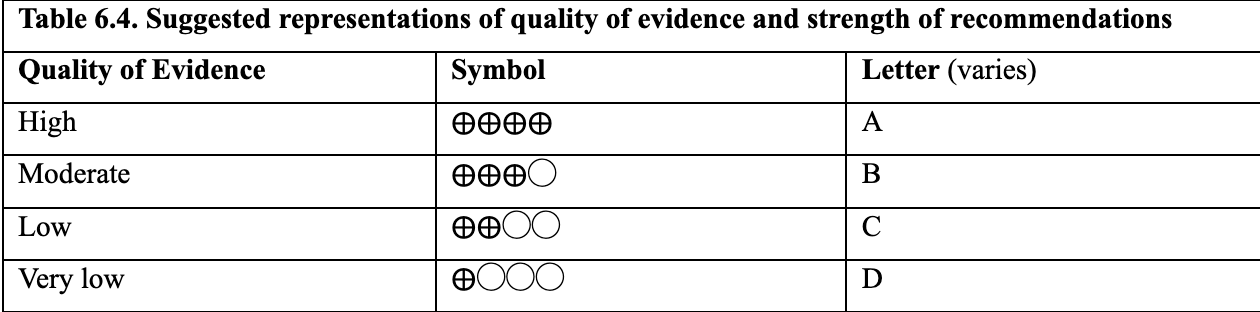 |
| 0-19 vs.  20-32 teeth (ref.) | Observational | 4 | Low | Low inconsistency  I^2^ = 0% | None | None | N.A. | Moderate | N.A. | None | Moderate  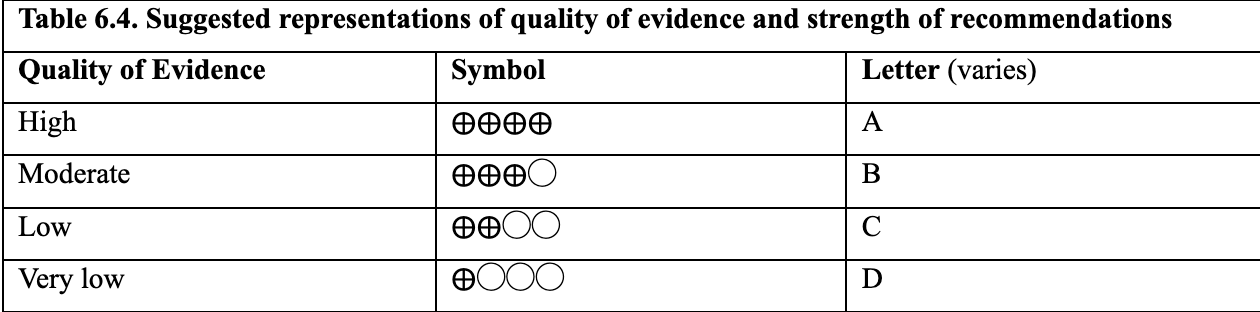 |
| 0 vs.  1-19 vs.  20-32 teeth (ref.) | Observational | 2 | Low | Moderate  inconsistency  I^2^ = 60% | None | None | N.A. | Large | Present | None | High  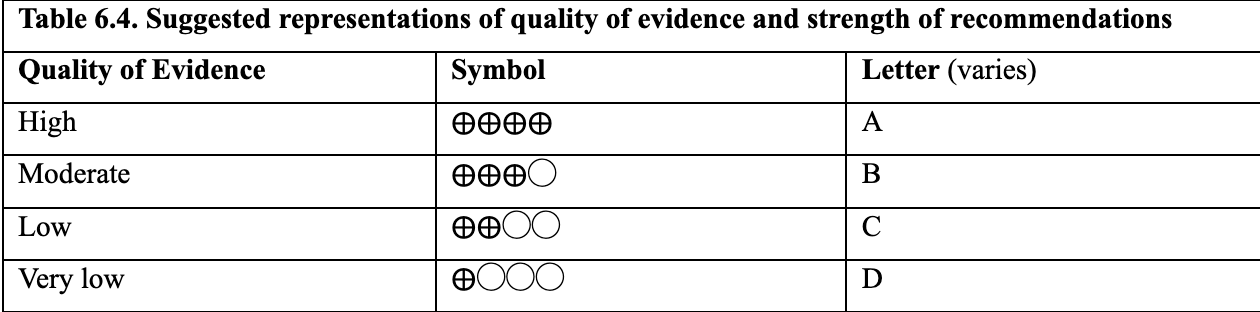 |
| 0-10 vs.  11-16 vs.  17-24 vs.  25-32 teeth (ref.) | Observational | 4 | Low | Serious inconsistency  I^2^ = 87% | None | None | N.A. | Large | Present | None | High  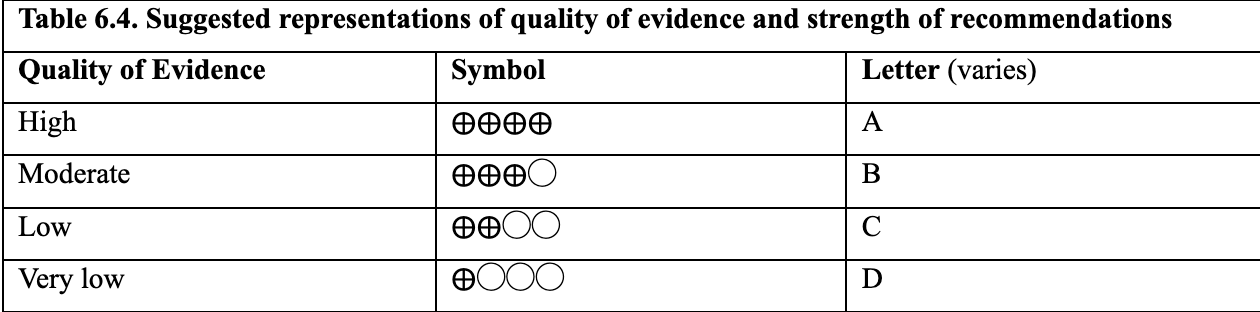 |

Abbreviations: ACVD, Atherosclerotic Cardiovascular Disease; vs., versus; ref., reference group; I^2^, I-square; N.A., Not Applicable

Cumulative incidence for categorical data of number of teeth and All-Cause Mortality

|  | **Study design** | **Number of studies** | **Study limitations** | **Inconsistency** | **Indirectness** | **Imprecision** | **Publication Bias** | **Magnitude of the effect** | **Dose-response gradient** | **Effect of plausible confounding** | **Strength of the evidence** |
| --- | --- | --- | --- | --- | --- | --- | --- | --- | --- | --- | --- |
| 0 vs.  1-32 teeth (ref.) | Observational | 14 | Low | Serious inconsistency  I^2^ = 100% | None | None | Minor | Moderate | N.A. | None | Low  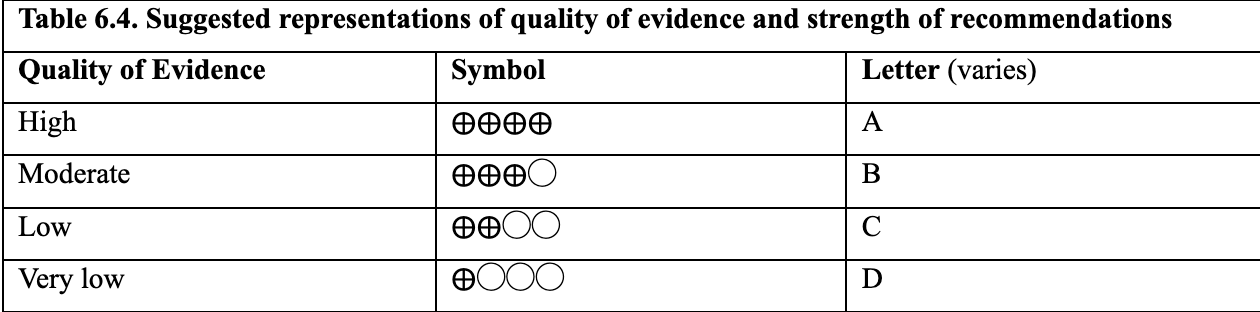 |
| 0-19 vs.  20-32 teeth (ref.) | Observational | 11 | Low | Serious inconsistency  I^2^ = 95% | None | None | Minor | Moderate | N.A. | None | Low  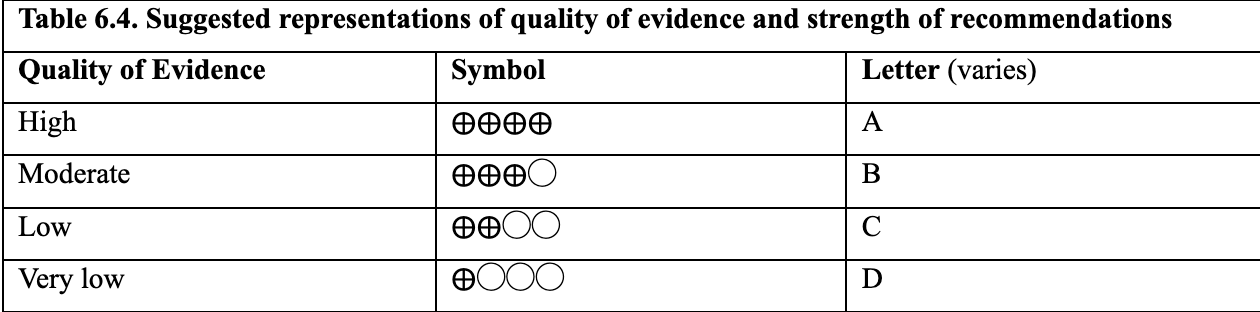 |
| 0 vs.  1-19 vs.  20-32 teeth (ref.) | Observational | 8 | Low | Serious inconsistency  I^2^ = 94% | None | None | N.A. | Large | Present | None | High  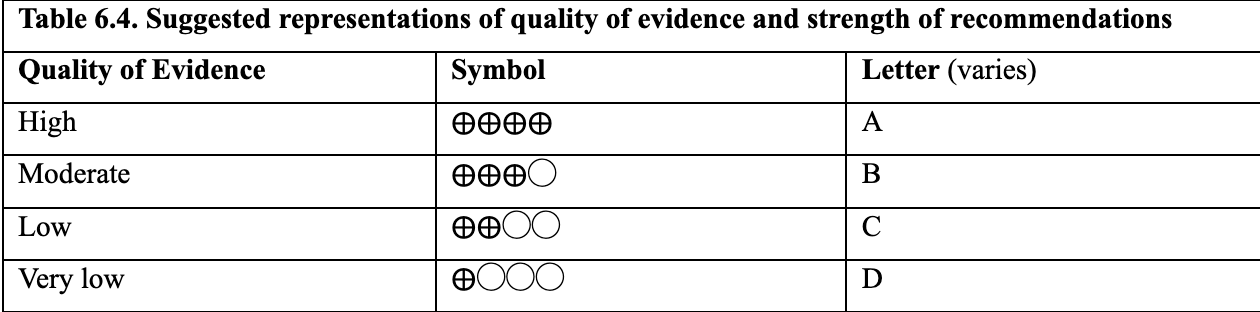 |

Abbreviations: vs., versus; ref., reference group; I^2^, I-square; N.A., Not Applicable

Incidence density for categorical and continuous data of number of teeth and ACVD (multivariable)

|  | **Study design** | **Number of studies** | **Study limitations** | **Inconsistency** | **Indirectness** | **Imprecision** | **Publication Bias** | **Magnitude of the effect** | **Dose-response gradient** | **Effect of plausible confounding** | **Strength of the evidence** |
| --- | --- | --- | --- | --- | --- | --- | --- | --- | --- | --- | --- |
| 0 vs.  1-32 teeth (ref.) | Observational | 5 | Low | Low  inconsistency  I^2^ = 35% | None | None | N.A. | Moderate | N.A. | None | Moderate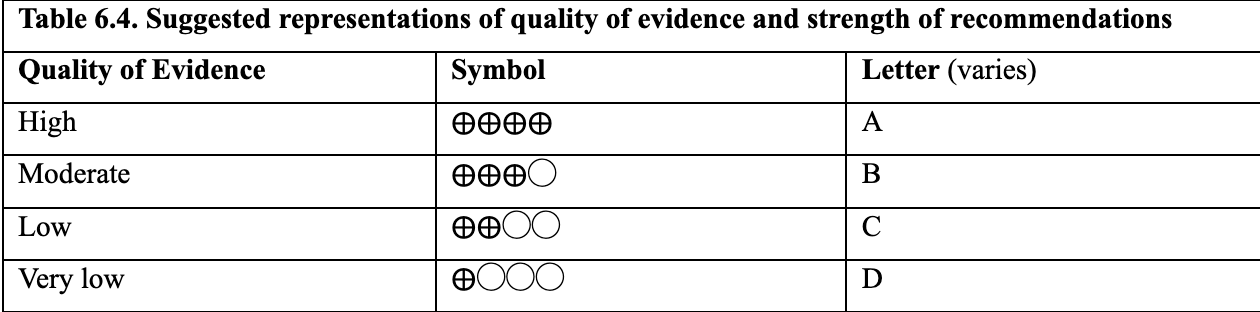 |
| 0-19 vs.  20-32 teeth (ref.) | Observational | 4 | Low | Low  inconsistency  I^2^ = 0% | None | None | N.A. | Moderate | N.A. | None | Moderate  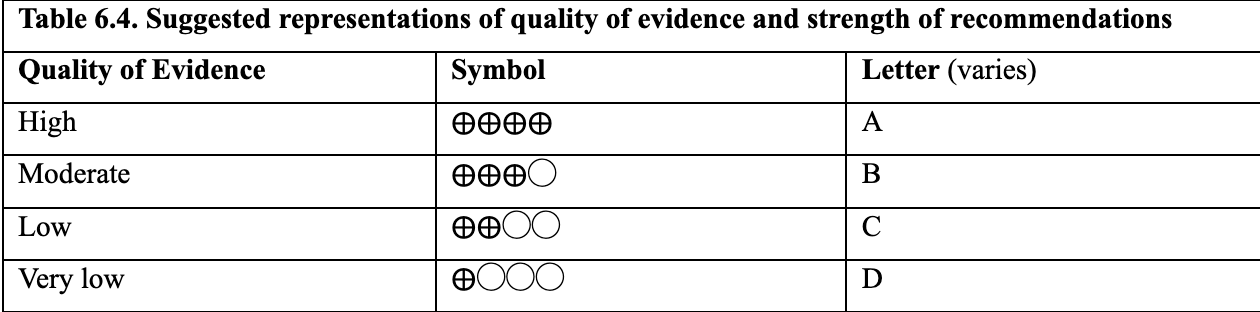 |
| Continuous no. of lost teeth | Observational | 13 | Moderate | Serious  inconsistency  I^2^ = 75% | None | None | Minor | Small | N.A. | None | Low  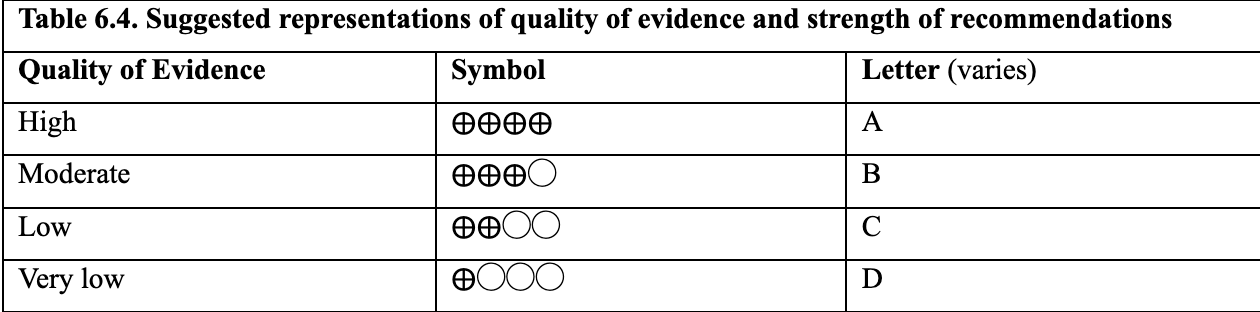 |

Abbreviations: ACVD, Atherosclerotic Cardiovascular Disease; vs., versus; ref., reference group; no., number; I^2^, I-square; N.A., Not Applicable

Incidence density for categorical data and continuous data of number of teeth and All-Cause Mortality (multivariable)

|  | **Study design** | **Number of studies** | **Study limitations** | **Inconsistency** | **Indirectness** | **Imprecision** | **Publication Bias** | **Magnitude of the effect** | **Dose-response gradient** | **Effect of plausible confounding** | **Strength of the evidence** |
| --- | --- | --- | --- | --- | --- | --- | --- | --- | --- | --- | --- |
| 0 vs.  1-32 teeth (ref.) | Observational | 7 | Moderate | Low  inconsistency  I^2^ = 20% | None | None | N.A. | Moderate | N.A. | None | Moderate  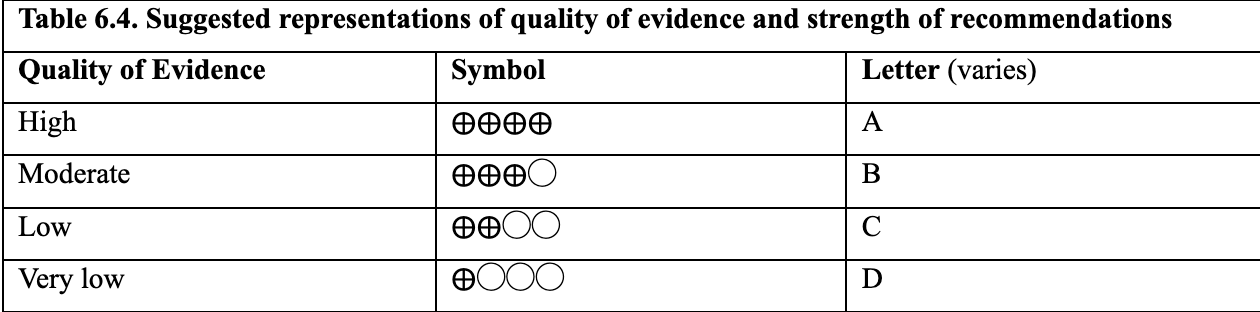 |
| 0-19 vs.  20-32 teeth (ref.) | Observational | 10 | Low | Serious  inconsistency  I^2^ = 78% | None | None | Minor | Moderate | N.A. | None | Low  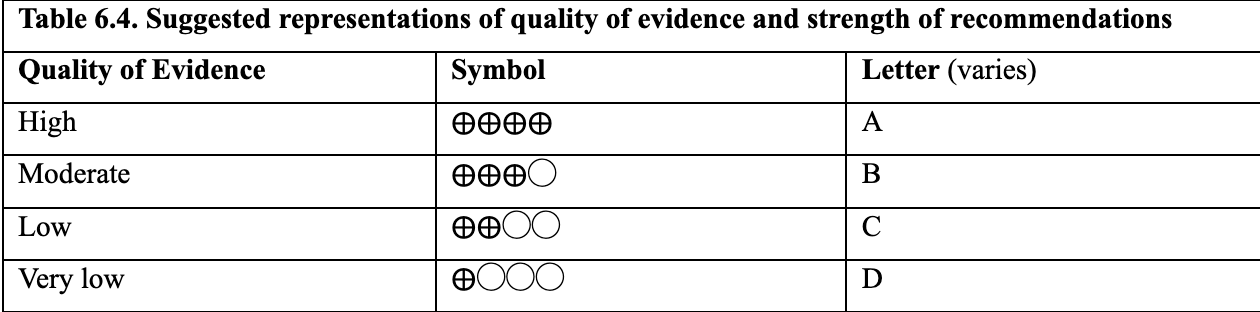 |
| Continuous no. of lost teeth | Observational | 11 | Moderate | Serious  inconsistency  I^2^ = 89% | None | None | Minor | Small | N.A. | None | Low  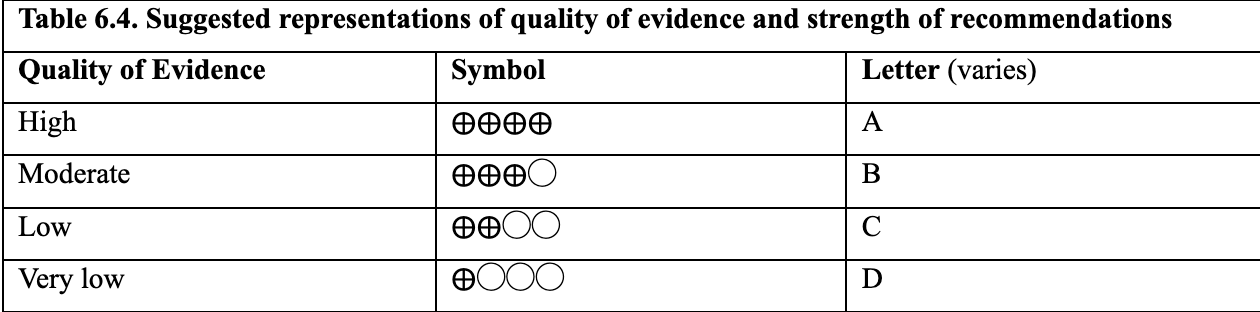 |

Abbreviations: vs., versus; ref., reference group; no., number; I^2^, I-square; N.A., Not Applicable
