# Supplementary material for: Lower Number of Teeth Is Related to Higher Risks for ACVD and Death—Systematic Review and Meta-Analyses of Survival Data
Source: Front Cardiovasc Med. 2021 May 7;8:621626. doi: 10.3389/fcvm.2021.621626 (PMC8138430; doi:10.3389/fcvm.2021.621626)
Supplement: Supplementary file 8 [file Table_1.docx]

Supplementary Table 1: Descriptive Information and Summary Results of the Studies Regarding Tooth loss and ACVD-related events (morbidity or mortality)

| **Author/ Year** | **Determinant** | **Main results (adjusted for multiple variables)**  **RR/HR (95%-CI)** | **Covariables** |
| --- | --- | --- | --- |
| Abnet ea. 2005 | Number of missing teeth continuous | **HD:** 1.28 (1.17 – 1.40)  **Stroke:** 1.11 (1.01 – 1.23) | 1, 2, 5, 8, 9, 28 |
| Aida ea. 2011 | ≥20 teeth, ≤19 teeth and can eat everything, ≤19 teeth and eating difficulty | **ACVD:**  ≤19 and eating difficulty: 1.83 (1.12 – 2.98)  ≤19 and eat everything: 1.16 (0.63 – 2.13)  ≥20 teeth: 1.00 (reference) | 1, 2, 4, 5, 9, 10, 11, 32, 35 |
| Ajwani ea. 2003 | Dentate/ edentulous | **ACVD:**  Edentulous: 1.40 (0.76 – 2.59)  Dentate: 1.00 (reference) | 1, 2, 4, 5, 7, 8, 9, 19 |
| Ajwani ea. 2003 | Dentate/ edentulous | **ACVD:** Edentulous: 1.46 (0.69 – 3.10)  Dentate: 1.00 (reference) | 1, 2, 4, 5, 7, 8, 9, 19 |
| Ando ea. 2014 | 0, 1-9, 10-19, ≥20 teeth | **ACVD:**  0: 1.36 (0.76 – 2.43)  1-9: 1.04 (0.56 – 1.94)  10-19 1.12 (0.59 – 2.12)  ≥20: 1.00 (reference) | 1, 4, 5, 7, 8, 9, 10 |
| Batty ea. 2018 | 0, 1-3, 4-6, ≥7 missing teeth  Continuous per 2 teeth lost | **CHD:**  Men:  ≥7: 1.05 (0.99 – 1.12)  4-6: 1.02 (0.97 – 1.07)  1-3: 1.03 (1.01 – 1.05)  0: 1.00 (reference)  per 2 teeth lost: 1.01 (1.00 – 1.02)  Women:  ≥7: 1.11 (1.01 – 1.21)  4-6: 1.08 (0.99 – 1.19)  1-3: 1.01 (0.96 – 1.07)  0 missing teeth: 1.00 (reference)  per 2 teeth lost: 1.01 (1.00 – 1.02) | 1, 4, 5, 6, 7, 8, 9, 10, 11, 20 |
| Brown ea. 2009 | Dentate/ edentulous | **ACVD:**  Edentulous  18-64 yrs.: 1.4 (1.1 – 1.7)  ≥65 yrs.: 1.2 (1.1 – 1.3)  Dentate: 1.0 (reference) | 1, 2, 3, 4, 6, 9, 19, 32, 33 |
| Cabrera ea. 2005 | ≤9 and >10 missing teeth | **ACVD-mortality:**  >10: 1.34 (1.05 – 1.71)  **Stroke-morbidity:**  >10: 1.30 (1.01 – 1.67)  **MI-morbidity:**  >10: 1.45 (1.14 – 1.83)  ≤9: 1.00 (reference) | 1, 4, 9, 5, 34 |
| Chang ea. 2019 | 0, 1-7, 8-14, 15-21, ≥22 missing teeth | **Heart Failure:**  ≥22: 1.31 (1.11 – 1.55)  15-21: 1.03 (0.83 – 1.27)  8-14: 1.05 (0.92 – 1.20)  1-7: 1.04 (0.99 – 1.09)  0: 1.00 (reference) | 1, 2, 4, 5, 6, 7, 8, 9, 10, 11, 22, 23, 26, 27, 54, 55, 56 |
| Chang ea. 2020 | 0, 1-7, 8-14, ≥15 missing teeth | **Stroke:**  ≥15: 1.28 (1.12 – 1.45)  8-14: 1.17 (1.02 – 1.33)  1-7: 1.05 (0.99 – 1.11)  0: 1.00 (reference) | 1, 2, 4, 5, 6, 7, 8, 9, 10, 11, 22, 23, 26, 27, 28, 29, 31, 47, 56 |
| Choe ea. 2009 | 0, 1-3, 4-6, ≥7 missing teeth | **Stroke:**  Men:  ≥7: 1.3 (1.2 – 1.4)  4-6: 1.3 (1.2 – 1.3)  1-3: 1.1 (1.1 – 1.1)  0: 1.0 (reference)  Women:  ≥7: 1.2 (1.0 – 1.3)  4-6: 1.1 (1.0 – 1.3)  1-3: 1.0 (0.9 – 1.0)  0: 1.0 (reference) | 1, 5, 6, 7, 8, 9, 10, 11 |
| Darnaud ea. 2019 | ≤10 and >10 missing teeth | **ACVD-mortality:**  >10 missing teeth: 1.18 (0.83 – 1.68)  ≤10 missing teeth: 1.00 (reference) | 1, 2, 4, 5, 6, 7, 8, 9 |
| Del Brutto ea. 2017 | <10, >10 teeth | **Stroke:**  <10: IRR = 5.06 (2.28 – 7.84)  >10: IRR = 1.81 (0.54 – 3.08) | 1, 2, 4, 5, 6, 7, 9, 11, 16 |
| Dietrich ea. 2008 | Dentate/edentulous | **Total incident CHD:**  Edentulous <60 yrs.: 1.90 (0.92 – 3.93)  Edentulous ≥60 yrs.: 1.61 (0.95 – 2.73)  Dentate: 1.00 (reference)  **Fatal incident CHD:**  Edentulous ≥60 yrs.: 4.21 (1.57 – 11.3)  Dentate: 1.00 (reference) | 1, 4, 5, 6, 7, 8, 9, 10, 12 |
| Goto ea. 2020 | 0-9, 10-19, ≥20 teeth  Continuous per 1 tooth lost | **ACVD-mortality:**  0-9: 0.92 (0.67 – 1.28)  10-19: 1.18 (0.84 – 1.65)  ≥20: 1.00 (reference)  per 1 tooth lost: 1.00 (0.98 – 1.01) | 1, 2, 4, 5, 6, 8, 9, 10, 11, 12 |
| Heitmann ea. 2008 | 0, 1-10, 11-22, 23-27, 28-32 teeth | **ACVD:**  Men:  0: 1.51 (0.90- 2.52)  1-10:1.41 (0.86 – 2.29)  11-22: 1.66 (1.04 – 2.66)  23-27: 1.21 (0.79 – 1.87)  28-32: 1.00 (reference)  Women:  0: 1.63 (0.91 – 2.93)  1-10: 1.39 (0.79 – 2.43)  11-22: 1.01 (0.59 – 1.71)  23-27: 1.26 (0.75 – 2.11)  28-32: 1.00 (reference)  **CHD:**  Men:  0: 1.75 (0.88 – 3.46)  1-10: 1.86 (0.95 – 3.64)  11-22: 1.21 (0.63 – 2.34)  23-27: 1.24 (0.68 – 2.28)  28-32: 1.00 (reference)  Women:  0: 0.57 (0.19 – 1.70)  1-10: 0.92 (0.32 – 2.71)  11-22: 1.10 (0.40 – 3.01_)  23-27: 0.42 (0.15 – 1.21)  28-32: 1.00 (reference)  **Stroke:**  Men:  0: 2.43 (0.95 – 6.28)  1-10: 1.91 (0.71 – 5.15)  11-22: 1.34 (0.52 – 3.44)  23-27: 1.07 (0.42 – 2.76)  28-32: 1.00 (reference)  Women:  0: 5.32 (1.98 – 14.3)  1-10: 5.39 (1.77 – 16.4)  11-22: 4.44 (1.45 – 13.5)  23-27: 3.03 (1.01 – 9.16)  28-32: 1.00 (reference) | 1, 2, 4, 5, 6, 8, 9, 10, 12 |
| Holmlund ea. 2010 | 0-9, 10-14, 15-19, 20-25, >25 teeth | **ACVD:**  0-9: 4.63 (2.95 – 7.26)  10-14: 3.29 (2.17 – 5.00)  15-19: 2.75 (1.87 – 4.04)  20-25: 1.79 (1.25 – 2.58)  >25: 1.00 (reference)  **CHD:**  0-9: 7.33 (4.11 – 13.07)  10-14: 3.30 (1.82 – 5.99)  15-19: 3.45 (2.04 – 5.84)  20-25: 1.94 (1.17 – 3.21)  >25: 1.00 (reference)  **Stroke:**  0-9: 2.01 (0.78 – 5.16)  10-14: 1.77 (0.79 – 3.99)  15-19: 2.22 (1.11 – 4.43)  20-25: 1.61 (0.85 – 3.06)  >25: 1.00 (reference) | 1, 2, 5 |
| Holmlund ea. 2017 | Quintiles of number of teeth | **ACVD:**  Q5: IRR = 1.61 (1.22 – 2.13)  Q4: IRR = 1.53 (1.16 – 2.03)  Q3: IRR = 1.43 (1.08 – 1.87)  Q2: IRR = 1.44 (1.07 – 1.95)  Q1: IRR = 1.00 (reference)  N of teeth: IRR = 0.88 (0.83 – 0.95)  **MI:**  Q5: IRR = 1.64 (1.12 – 2.42)  Q4: IRR = 1.58 (1.07 – 2.31)  Q3: IRR = 1.52 (1.05 – 2.21)  Q2: IRR = 1.57 (1.05 – 1.08)  Q1: IRR = 1.00 (reference)  N of teeth: IRR = 0.90 (0.82 – 0.99)  **Stroke:**  Q5: IRR = 1.32 (0.84 – 2.07)  Q4: IRR = 1.44 (0.92 – 2.24)  Q3: IRR = 1.24 (0.79 – 1.93)  Q2: IRR = 1.36 (0.83 – 2.22)  Q1: IRR = 1.00 (reference)  N of teeth: IRR = 0.93 (0.84 – 1.02)  **Heart Failure:**  Q5: IRR = 1.75 (0.92 – 3.31)  Q4: IRR = 1.28 (0.67 – 2.45)  Q3: IRR = 1.24 (0.65 – 2.36)  Q2: IRR = 1.09 (0.51 – 2.29)  Q1: IRR = 1.00 (reference)  N of teeth: IRR = 0.87 (0.77 – 0.99) | 1, 2, 4, 5 |
| Hung ea. 2004 | Number of missing teeth continuous  0-10, 11-16, 17-24, 25-32 teeth | **CHD**:  Men:  Continuous: 0.94 (0.82 – 1.09)  0-10: 1.36 (1.11 – 1.67)  11-16: 1.35 (1.06 – 1.72)  17-24: 1.10 (0.95 – 1.26)  25-32: 1.00 (reference)  Women:  0-10: 1.64 (1.31 – 2.05)  11-16: 1.34 (0.97 – 1.87)  17-24: 1.14 (0.92 – 1.42)  25-32: 1.00 (reference) | 1, 4, 5, 6, 7, 9, 10, 11, 19, 20, 21, 28, 31, 45 |
| Hung ea. 2003 | Number of missing teeth continuous  0-10, 11-16, 17-24, 25-32 teeth | **PAD:**  Continuous: 1.39 (1.07 – 1.82)  0-10: 1.05 (0.68 – 1.63)  11-16: 1.43 (0.89 – 2.31)  17-24: 1.19 (0.90 – 1.85)  25-32: 1.00 (reference) | 1, 4, 5, 6, 7, 9, 10, 11, 19, 20, 28, 31, 45 |
| Iwasaki ea. 2017 | Number of teeth | **Stroke-related medical costs per month:**  Number of teeth: Coefficient estimated = -227 (-423 to -32) (p = 0.018)  **Stroke-related hospitalization:**  Number of teeth: IRR = 0.95 (0.90 – 0.99) (p = 0.040) | 2, 4, 5, 8, 9, 10, 11, 19 |
| Janket ea. 2014 | 0, 1-10, 11-20, >20 teeth | **ACVD:**  >20: 0.40 (0.18 – 0.90)  11-20: 0.64 (0.29 – 1.44)  1-10: 1.06 (0.59 – 1.92)  0: 1.00 (reference) | 1, 2, 4, 5, 6, 7, 8, 14, 15 |
| Janket ea. 2013 | Natural teeth (NT) on both arches, combination of partial dentures (PD) and NT, combination of PD and full denture (FD), FD’s | **ACVD:**  PD/FD: 2.27 (1.06 – 4.87)  FD/FD: 1.60 (0.78 – 3.29)  NT/PD: 0.62 (0.14 – 2.66)  NT/NT: 1.00 (reference) | 1, 2, 4, 5, 6, 8 |
| Joshipura ea. 2003 | Number of missing teeth continuous  0-10, 11-16, 17-24, 25-32 teeth OR 0-24 vs. ≥25 teeth | **Stroke:**  Continuous: 1.27 (0.97 – 1.67)  0-10: 1.62 (1.22 – 1.96)  11-16: 1.68 (1.04 – 2.70)  17-24: 1.49 (1.14 – 1.96)  25-32: 1.00 (reference)  0-24: 1.55 (1.22 – 1.96)  25-32: 1.00 (reference) | 1, 4, 5, 6, 7, 9, 10, 11, 20, 31, 45 |
| Joshipura ea. 1996 | 0-10, 11-16, 17-24, 25-32 teeth | **CHD:**  0-10: 1.29 (0.96 – 1.73)  11-16: 1.04 (0.71 – 1.54)  17-24: 1.03 (0.83 – 1.27)  25-32: 1.00 (reference) | 1, 5, 9, 10, 11, 16, 20, 45 |
| Joshy ea. 2016 | 0, 1-9, 10-19, ≥20 teeth | **IHD:**  0: 1.10 (0.95 – 1.26)  1-9: 1.20 (1.06 – 1.35)  10-19: 1.05 (0.96 – 1.15)  ≥20: 1.00 (reference)  **Heart failure:**  0: 1.97 (1.27 – 3.07)  1-9: 2.04 (1.35 – 3.09)  10-19: 1.50 (1.04 – 2.18)  ≥20: 1.00 (reference)  **PVD:**  0: 2.53 (1.81 – 3.52)  1-9: 1.73 (1.23 – 2.44)  10-19: 1.67 (1.27 – 2.19)  ≥20: 1.00 (reference)  **Stroke:**  0: 1.20 (0.90 – 1.62)  1-9: 0.90 (0.59 – 1.40)  10-19: 1.11 (0.72 – 1.73)  ≥20: 1.00 (reference) | 1, 2, 4, 5, 9, 11, 33 |
| Kebede ea. 2017 | Number of missing teeth continuous | **ACVD:**  1.02 (0.99 – 1.05) | 1, 2, 4, 5, 9, 11, 26 |
| LaMonte ea. 2017 | Dentate/ edentulous | **ACVD-mortality:**  Edentulous: 1.07 (0.81 – 1.40)  Dentate: 1.00 (reference)  **ACVD-morbidity**  Edentulous: 1.07 (0.93 – 1.23)  Dentate: 1.00 (reference)  **CHD:**  Edentulous: 1.10 (0.90 – 1.34)  Dentate: 1.00 (reference)  **Stroke:**  Edentulous: 0.77 (0.55 – 1.06)  Dentate: 1.00 (reference) | 1, 3, 4, 5, 6, 7, 8, 9, 10, 11, 16, 26 |
| Lee ea. 2019 | 0, 1-4, 5-14, 15-27, 28 missing teeth  Number of missing teeth continuous | **MI:**  28: 1.07 (0.89 – 1.29)  15-27: 1.24 (1.11 – 1.31)  5-14: 1.13 (1.08 – 1.19)  1-4: 1.08 (1.05 – 1.12)  0: 1.00 (reference)  Continuous: 1.010 (1.007 – 1.014)  **Stroke:**  28: 1.30 (1.13 – 1.50)  15-27: 1.28 (1.18 – 1.39)  5-14: 1.26 (1.20 – 1.32)  1-4: 1.12 (1.09 – 1.16)  0: 1.00 (reference)  Continuous: 1.015 (1.012 – 1.018)  **Heart Failure:**  28: 1.52 (1.31 – 1.77)  15-27: 1.39 (1.29 – 1.51)  5-14: 1.17 (1.11 – 1.23)  1-4: 1.04 (0.99 – 1.08)  0: 1.00 (reference)  Continuous: 1.016 (1.013 – 1.019) | 1, 2, 4, 5, 6, 7, 8, 9, 10, 11, 22, 49 |
| Li ea. 2010 | 0, 1-21, ≥22 teeth | **CHD:**  0: 1.48 (1.24 – 1.78)  1-21: 1.24 (0.98 – 1.56)  ≥22: 1.00 (reference)  **CVA:**  0: 1.10 (0.87 – 1.38)  1-21: 1.24 (1.03 – 1.49)  ≥22: 1.00 (reference)  **ACVD:**  0: 1.35 (1.05 – 1.74)  1-21: 1.32 (1.06 – 1.65)  ≥22: 1.00 (reference) | 1, 2, 4, 5, 6, 7, 8, 9, 10, 11, 19, 22, 24, 25, 35, 36 |
| Liljestrand ea. 2015 | 0-1, 2-4, 5-8, 9-31, 32 missing teeth | **ACVD:**  32: 1.40 (1.01 – 1.95)  9-31: 1.51 (1.13 – 2.02)  5-8: 1.18 (0.85 – 1.63)  2-4: 1.10 (0.80 – 1.51)  0-1: 1.00 (reference)  **CHD:**  32: 1.65 (1.09 – 2.50)  9-31: 1.99 (1.37 – 2.89)  5-8: 1.62 (1.08 – 2.43)  2-4: 1.22 (0.81 – 1.85)  0-1: 1.00 (reference) **MI:**  32: 1.84 (0.99 – 3.42)  9-31: 2.10 (1.19 – 3.71)  5-8: 2.39 (1.32 – 4.31)  2-4: 1.41 (0.76 – 2.63)  0-1: 1.00 (reference)  **Stroke:**  32: 1.17 (0.71 – 1.92)  9-31: 1.05 (0.67 – 1.65)  5-8: 0.61 (0.35 – 1.06)  2-4: 0.95 (0.59 – 1.54)  0-1: 1.00 (reference) | 1, 2, 4, 5, 6, 7, 8, 19, 25 |
| Morrison ea. 1999 | Dentate/edentulous | **CHD:**  Edentulous: RR = 1.90 (1.17 – 3.10)  Dentate: RR = 1.00 (reference)  **ACVD:**  Edentulous: RR = 1.63 (0.77 – 3.42)  Dentate: RR = 1.00 (reference) | 1, 2, 4, 5, 6, 7, 8 |
| Mucci ea. 2009 | No teeth left or just a few; about half the teeth left; nearly all or all teeth left. | **ACVD:**  No teeth left or just a few:  1.2 (1.1 – 1.4)  About half the teeth left:   - 1. (1.0 – 1.2)   Nearly all or all teeth left:  1.0 (reference) | 1, 2, 4, 5, 6, 8, 9, 47 |
| Munoz-Torres ea. 2017 | 0, 1-10, 11-16, 17-24, 25-32 teeth  0, ≥1 and 0, 1-2, 3-4, ≥5 missing teeth | **PAD:**  **Number of teeth:**  0: 0.87 (0.54 – 1.41)  1-10: 0.86 (0.55 – 1.34)  11-16: 1.51 (1.01 – 2.26)  17-24: 0.88 (0.65 – 1.21)  25-32: 1.00 (reference)  **Missing teeth:**  ≥1: 1.31 (>1.00 – 1,71)  0: 1.00 (reference)  1-2: 1.40 (1.04 – 1.88)  3-4: 1.27 (0.70 – 2.30)  ≥5: 1.09 (0.57 – 2.09)  0: 1.00 (reference) | 1, 5, 6, 7, 8, 9, 10, 11, 19, 21, 44, 57 |
| Noguchi ea. 2014 | <5, ≥5 missing teeth | **MI:**  ≥5: OR = 1.97 (0.71 – 5.45)  <5: OR = 1.00 (reference) | 1, 5, 6, 7, 8, 9, 19 |
| Oluwagbemigun ea. 2015 | 0, 1-17, 18-23, 24-27, 28-32 teeth | **MI:** 0: 2.91 (1.74 – 4.86)  1-17: 0.77 (0.36 – 1.68)  18-23: 1.64 (1.02 – 2.64)  24-27: 1.59 (1.02 – 2.48)  28-32: 1.00 (reference)  **Stroke:**  0: 1.95 (1.13 – 3.39)  1-17: 1.06 (0.48 – 2.36)  18-23: 1.64 (1.05 – 2.63)  24-27: 1.01 (0.64 – 1.62)  28-32: 1.00 (reference) | 1, 2, 4, 5, 6, 8, 9, 10, 11, 16, 19, 32, 45, 46 |
| Park ea. 2019 | 0, 1-7, 8-14, 15-21, 22-28 missing teeth | **ACVD-mortality:**  22-28: 1.43 (1.23 – 1.66)  15-21: 1.27 (1.11– 1.45)  8-14: 1.16 (1.06 – 1.27)  1-7: 1.04 (0.99– 1.08)  0: 1.00 (reference) | 1, 2, 4, 6, 7, 8, 9, 11, 23, 26, 27, 28, 29, 31 |
| Qi ea. 2020 | Number of missing teeth continuous | **ACVD-mortality:**  Continuous: 1.01 (0.98 – 1.03) | 1, 2, 4, 5, 6, 8, 9, 10 |
| Ragnarsson ea. 2004 | Dentate/edentulous  Number of remaining teeth continuous | **CHD-mortality:**  Edentulous: 1.46 (0.88 – 2.43)  Dentate: 1.00 (reference)  Continuous: 0.98 (0.96 – 1.01) | 1, 2, 4, 5, 7, 8 |
| Reichert ea. 2015 | Number of missing teeth continuous | **ACVD:**  Continuous: 0.99 (0.96 – 1.03) | 1, 2, 5, 6, 7, 9, 14, 17, 22, 27, 42, 43 |
| Reichert ea. 2016 | 0-10, 11-24 missing teeth | **ACVD:**  11-24: 1.22 (0.84 – 1.78)  0-10: 1.00 (reference) | 1, 2, 5, 6, 9, 14, 18, 19 |
| Saito ea. 2019 | 0, 1-9, 10-19, 20-27, 28 teeth | **ACVD:**  0: 1.32 (0.36 – 4.83)  1-9: 2.39 (0.66 – 8.67)  10-19: 1.25 (0.42 – 3.74)  20-27: 1.30 (0.42 – 4.06)  28: 1.00 (reference)  **CVD:**  0: 2.82 (0.46 – 17.25)  1-9: 1.60 (0.27 – 9.49)  10-19: 5.32 (1.11 – 25.48)  20-27: 1.46 (0.33 – 6.44)  28: 1.00 (reference) | 1, 2, 5, 9 |
| Schwahn ea. 2013 | 0, 1-9, 10-19 teeth | **ACVD:**  10-19: 1.08 (0.68 – 1.71)  1-9: 0.90 (0.59 – 1.38)  0: 1.00 (reference) | 1, 2, 4, 5, 6, 8, 9, 10, 11, 12, 26 |
| Tu ea. 2007 | Number of missing teeth continuous  0-4, 5-8, ≥9 missing teeth | **ACVD:**  Continuous: 1.01 (0.99 – 1.03)  ≥9: 1.35 (1.03 – 1.77)  5-9: 1.14 (0.94 – 1.39)  0-4: 1.00 (reference)  **CHD:**  Continuous: 1.01 (0.98 – 1.03)  ≥9: 1.19 (0.84 – 1.69)  5-9: 1.18 (0.94 – 1.50)  0-4: 1.00 (reference)  **Stroke:**  Continuous: 1.02 (0.98 – 1.06)  ≥9: 1.64 (0.96 – 2.80)  5-9: 0.88 (0.55 – 1.41)  0-4: 1.00 (reference) | 1, 2, 4, 5, 8, 9 |
| Tuominen ea. 2003 | ≤10, 11-24, ≥25 teeth | **CHD:** Men:  ≤10: 0.9 (0.5 – 1.6)  11-24: 0.8 (0.5 – 1.3)  ≥25: 1.0 (reference)  Women:  ≤10: 0.3 (0.1 – 1.0)  11-24: 0.5 (0.2 – 1.8)  ≥25: 1.0 (reference) | 1, 4, 5, 6, 7, 8, 23, 26, 29 |
| Vedin ea. 2016 | 0, 1-14, 15-19, 20-25, 26-32 teeth | **ACVD:**  0: 1.85 (1.45 – 2.37)  1-14: 1.59 (1.32 – 1.91)  15-19: 1.36 (1.21 – 1.54)  20-25: 1.17 (1.10 – 1.24)  26-32: 1.00 (reference) **MI:**  0: 0.97 (0.77 – 1.23)  1-14: 0.98 (0.82 – 1.17)  15-19: 0.99 (0.88 – 1.11)  20-25: 0.99 (0.94 – 1.05)  26-32: 1.00 (reference) **Stroke:**  0: 1.67 (1.15 – 2.39)  1-14: 1.46 (1.11 – 1.92)  15-19: 1.29 (1.07 – 1.55)  20-25: 1.14 (1.04 – 1.24)  26-32: 1.00 (reference) | 1, 2, 4, 5, 6, 7, 8, 9, 10, 11, 19, 20, 22 |
| Vedin ea. 2017 | Number of remaining teeth continuous | **MACE:**  1.06 (1.02 – 1.10)  **ACVD death:**  1.17 (1.10 – 1.24)  **Stroke:**  1.14 (1.04 – 1.25) | 1, 2, 4, 5, 6, 7, 8, 9, 10, 11, 19, 22 |
| Vogtmann ea. 2017 | Number of missing teeth in quintiles | **ACVD mortality:**  Q5 (more teeth missing than expected): 1.33 (1.13 – 1.56)  Q4: 1.18 (1.02 – 1.35)  Q3 (teeth missing as expected): 1.23 (1.06 – 1.43)  Q2: 1.11 (0.94 – 1.30)  Q1 (fewer teeth missing than expected): 1.00 (reference) | 1, 2, 3, 4, 5, 53 |
| Watt et al. 2012 | Edentulous, natural teeth and dentures, natural teeth only | **ACVD:**  Edentulous: 1.49 (1.16 – 1.92)  Natural + dentures: 1.02 (0.78 – 1.34)  Natural teeth: 1.00 (reference)  **CHD:**  Edentulous: 1.22 (0.89 – 1.68)  Natural + dentures: 0.87 (0.62 – 1.22)  Natural teeth: 1.00 (reference) **Stroke:**  Edentulous: 2.97 (1.46 – 6.05)  Natural + dentures: 2.09 (1.01 – 4.34)  Natural teeth: 1.00 (reference) | 1, 2, 4, 5, 6, 8, 9, 10, 11, 12, 35 |
| Wu et al. 2000 | Dentate/edentulous | **CVA:**  Edentulous: 1.23 (0.91 – 1.66)  Dentate: 1.00 (reference) | 1, 2, 3, 4, 5, 6, 7, 8, 9, 10 |

Abbreviations: RR, Risk Ratio; HR, Hazard Ratio; OR, Odds Ratio; 95%-CI, 95%-Confidence Interval; Q, quintile; HD, Heart Disease; ACVD, Atherosclerotic Cardiovascular Disease; CHD, Coronary Heart Disease; MI, Myocardial Infarction; PAD, Peripheral Arterial Disease; PVD, Peripheral Vascular Disease; CVD, Cerebrovascular Disease; CVA, Cerebrovascular Accident

Covariables: (1) age; (2) sex; (3) race or ethnicity; (4) socioeconomic status (income and/or education, profession, place of residence, living situation, husband’s occupation, number of family members living together, household income or wealth index); (5) smoking status or smoking history; (6) diabetes (hemoglobin A1c/fasting serum glucose/plasma glucose/glycemia or medical history of diabetes); (7) hyperlipidemia (low-density lipoprotein cholesterol and/or high-density lipoprotein cholesterol and/or triglycerides or serum total cholesterol level or dyslipoproteinemia or dyslipidemia or cholesterol); (8) hypertension (systolic and/or diastolic blood pressure) or history of hypertension or resting heart rate; (9) body mass index or waist/hip ratio or obesity or height and weight or abdominal skinfold thickness; (10) alcohol consumption or drinking status; (11) physical activity/function or time spent walking daily or walking speed or activity of daily living or exercise; (12) marital status or partnership; (13) microalbuminuria or serum albumin; (14) C-reactive protein; (15) fibrinogen; (16) diet; (17) IL-6; (18) statin intake/lipid lowering drugs; (19) ACVD/history of ACVD (myocardial infarction, ischemic heart disease, cerebrovascular disease, stroke, angina pectoris, transitory ischemic attack, cardiac disease, peripheral arterial disease, baseline degree of carotid stenosis, diagnosis of heart failure, macrovascular or microvascular disease, circulation disease); (20) family history of ACVD; (21) menopausal status and hormonal use; (22) renal disease or creatinine level or estimated glomerular filtration rate; (23) papillary bleeding score or bone loss or probing depth or average Periodontal Index or average Gingival Index or periodontal disease; (24) dependent living or requiring assistance with daily activities or I-ADL (Instrumental Activities of Daily Living); (25) hypertension medication; (26) frequency of dental visits or dental checkup or regular dental prophylaxis/cleaning; (27) oral hygiene or frequency or brushing teeth and/or use of floss or interdental brushes; (28) missing teeth or tooth loss or number of teeth or difficulty of chewing; (29) DMFT index (decayed, missing, filled teeth) or dental caries; (30) family history of hypertension; (31) co-morbidities (number of chronic diseases or osteoarthritis, activity limitation/fatigue in daily activities, rheumatoid arthritis, diabetes mellitus, ischemic heart disease, cerebrovascular disease, cancer, asthma, chronic bronchitis, emphysema, lung cancer, respiratory diseases, FEV 1.0 (Tiffeneau-Pinelli index), musculo-skeletal disease or present illness, malignancy history); (32) health insurance/dental insurance; (33) age at first birth/parity; (34) self-rated health or quality of life; (35) psychological distress score or Mini Mental State Exam Score or social activity or physical-mental health status or depressive symptoms or geriatric depression scale score; (36) energy intake; (37) protein intake; (38) tiredness or physical status; (39) number of drugs; (40) white blood cell count; (41) hemoglobin; (42) number of detected different bacterial species per individual; (43) caffeine intake; (44) multivitamin supplement use and/or Vitamin E use and/or mineral supplements and/or vitamin D intake and/or calcium supplements; (45) intake of antibiotics and/or NSAID (Non-Steroidal Anti-Inflammatory Drugs) and/or HRT (Hormone Replacement Therapy); (46) number of siblings and/or early-life exposures, and familial factors; (47) aspartate aminotransferase, alanine aminotransferase, gamma-glutamyl transferase; (48) cooperativeness with nursing staff and/or dental personnel; (49) medical history of pneumonia or chronic pulmonary disease; (50) cognitive impairment; (51) denture use; (52) falling experience; (53) opium use; (54) history of cancer; (55) liver panel; (56) proteinuria; (57) aspirin use
