# Supplementary material for: Lower Number of Teeth Is Related to Higher Risks for ACVD and Death—Systematic Review and Meta-Analyses of Survival Data
Source: Front Cardiovasc Med. 2021 May 7;8:621626. doi: 10.3389/fcvm.2021.621626 (PMC8138430; doi:10.3389/fcvm.2021.621626)
Supplement: Supplementary file 9 [file Table_2.docx]

Supplementary Table 2: Descriptive Information and Summary Results of the Studies Regarding Tooth loss and All-cause mortality

| **Author/Year** | **Determinant** | **Main results (adjusted for multiple variables)**  **RR/HR (95%-CI)** | **Covariables** |
| --- | --- | --- | --- |
| Abnet ea. 2005 | Number of missing teeth continuous | 1.13 (1.09 – 1.18) | 1, 2, 5, 8, 9, 28 |
| Adolph ea. 2017 | ≤10 and >10 missing teeth | >10: 2.02 (1.73 – 2.37)  ≤10: 1.00 (reference) | 1, 2, 4, 5, 6, 7, 8, 9, 10, 47 |
| Ajwani ea. 2003 | Dentate/ edentulous | Edentulous: 1.48 (0.95 – 2.31)  Dentate: 1.00 (reference) | 1, 2, 4, 5, 7, 8, 9, 19 |
| Ajwani ea. 2003 | Dentate/ edentulous | Edentulous: 1.31 (0.80 – 2.16)  Dentate: 1.00 (reference) | 1, 2, 4, 5, 7, 8, 9, 19 |
| Ando ea. 2014 | 0, 1-9, 10-19, ≥20 teeth | 0: 1.28 (0.97 – 1.68)  1-9: 1.24 (0.94 – 1.63)  10-19: 1.04 (0.77 – 1.41)  ≥20: 1.0 (reference) | 1, 4, 5, 7, 8, 9, 10 |
| Ansai ea. 2010 | Number of remaining teeth continuous | 0.98 (0.95 – 1.01) | 2, 4, 5, 6, 7, 9, 12, 13 |
| Brown ea. 2009 | Dentate/ edentulous | Edentulous  18-64 yrs.: 1.5 (1.3 – 1.7)  ≥65 yrs.: 1.3 (1.2 – 1.4)  Dentate: 1.0 (reference) | 1, 2, 3, 4, 6, 9, 19, 32, 33 |
| Cabrera ea. 2005 | Number of missing teeth | >10: 1.27 (1.09 – 1.47)  ≤9: 1.00 (reference) | 1, 4, 9, 5, 34 |
| Caplan ea. 2017 | Dentate/edentulous | Edentulous with complete upper and lower dentures: 1.42 (1.10 – 1.85)  Dentate: 1.00 (reference)  Edentulous without complete upper and lower dentures: 1.41 (1.00-1.99)  Dentate: 1.00 (reference) | 1, 2, 22, 48 |
| Darnaud ea. 2019 | ≤10 and >10 missing teeth | >10: 4.69 (4.23 – 5.14)  ≤10: 1.00 (reference) | 1, 2, 4, 5, 6, 7, 8, 9 |
| Dewake ea. 2020 | 0, 1-9, ≥10 teeth | ≥10 teeth: 6/54  1-9 teeth: 10/49  0 teeth: 12/70 | No adjustments |
| Fukai ea. 2008 | ≥10 functional teeth with or without dentures  <10 functional teeth with or without dentures | *Males (N=494 deaths/2248):*  ≥ 10 functional teeth left (N=213/1313);  < 10 functional teeth left (N=281/935)  < 10 functional teeth and using dentures (N=206/666)  < 10 functional teeth and not using dentures (N=75/269)  *Females (N=467 deaths/3440):*  ≥ 10 functional teeth left (N=113/1378);  < 10 functional teeth left (N=354/2062)  <10 functional teeth and using dentures (N= 247/1581)  <10 functional teeth and not using dentures (N=107/481) | No adjustments |
| Furuta ea. 2018 | ≤9 and ≥10 teeth | ≤9 teeth: 1.71 (0.90 – 3.25)  ≥10 teeth: 1.00 (reference) | 1, 2, 31 |
| Garcia ea. 1998 | Number of remaining teeth continuous | Continuous: 1.01 (0.98 – 1.04) | 1, 4, 5, 8, 20, 23 |
| Goto ea. 2020 | 0-9, 10-19, ≥20 teeth  Continuous per 1 tooth lost | 0-9 teeth: 1.19 (1.03 – 1.39)  10-19 teeth: 1.18 (1.01 – 1.39)  ≥20 teeth: 1.00 (reference)  per 1 tooth lost: 1.01 (1.00 – 1.01) | 1, 2, 4, 5, 6, 8, 9, 10, 11, 12 |
| Hamalainen ea. 2003 | Number of missing teeth continuous | Continuous: 1.03 (1.00 – 1.05) | 2, 32, 35 |
| Hayasaka ea. 2013 | 0-9, 10-19, ≥20 teeth | 0-9 with brushing teeth ≥2/day: 1.19 (1.03 – 1.36)  0-9 with brushing teeth <2/day: 1.38 (1.21 – 1.58)  10-19 with brushing teeth ≥2/day: 1.03 (0.86 – 1.22)  10-19 with brushing teeth <2/day: 1.26 (1.06 – 1.50)  ≥20 teeth: 1.00 (reference)  0-9 with dental visits: 1.09 (0.95 – 1.26)  0-9 with no dental visits: 1.45 (1.27 – 1.65)  10-19 with dental visits: 1.02 (0.86 – 1.21)  10-19 with no dental visits: 1.42 (1.19 – 1.69)  ≥20 teeth: 1.00 (reference)  0-9 with use of dentures: 1.24 (1.10 – 1.40)  0-9 with no use of dentures: 1.73 (1.47 – 2.04)  10-19 with use of dentures: 1.11 (0.95 – 1.29)  10-19 with no use of dentures: 1.34 (1.09 1.64)  ≥20 teeth: 1.00 (reference) | 1, 2, 4, 5, 6, 8, 9, 10, 11, 19, 36, 37, 38 |
| Hiratsuka ea. 2020 | 0, 1-9, 10-19, ≥20 teeth | 0: 1.84 (1.30 – 2.59)  1-9: 1.75 (1.28 – 2.40)  10-19: 1.11 (0.80 – 1.54)  ≥20: 1.00 (reference) | 1, 2, 4, 5, 6, 8, 10, 11, 19, 35, 49, 50 |
| Hirotomi ea. 2015 | Number of remaining teeth continuous  0, 1-9, 10-19, 20-28 teeth | Continuous: 0.96 (P = 0.047)  20-28: 0.38 (P = 0.036)  < 20 teeth: 1.00 (reference) | 2, 4, 5, 9, 13, 19 |
| Hoke ea. 2011 | Dentate/ edentulous | Edentulous: 1.99 (1.18 – 3.02)  Dentate: 1.00 (reference) | 1, 2, 5, 6, 7, 8, 9, 18, 19 |
| Holm-Pedersen ea. 2008 | Dentate/edentulous | Edentulous: 1.26 (1.03 – 1.55)  Dentate: 1.00 (reference) | 4, 5, 8, 19, 32, 39 |
| Holmlund ea. 2010 | 0-9, 10-14, 15-19, 20-25, >25 teeth | 0-9: 2.68 (1.96 – 3.67)  10-14: 2.01 (1.52 – 2.66)  15-19: 1.77 (1.37 – 2.27)  20-25: 1.28 (1.02 – 1.60)  >25 teeth: 1.00 (reference) | 1, 2, 5 |
| Hu ea. 2015 | 0, 1-9, 10-19, ≥20 teeth | 0: 1.36 (1.15 – 1.61)  1-9: 1.24 (1.08 – 1.42)  10-19: 1.19 (1.09 – 1.31)  ≥20: 1.00 (reference) | 1, 2, 4, 5, 6, 7, 8, 9, 10, 12, 26 |
| Janket ea. 2014 | 0, 1-10, 11-20, >20 teeth | >20: 0.79 (0.47 – 1.35)  11-20: 0.70 (0.37 – 1.33)  1-10: 1.05 (0.64 – 1.72)  0: 1.00 (reference) | 1, 2, 4, 5, 6, 7, 8, 14, 15 |
| Joshy, ea. 2016 | 0, 1-9, 10-19, ≥20 teeth | 0: 1.60 (1.37 – 1.87)  1-9: 1.67 (1.45 – 1.93)  10-19: 1.27 (1.13 – 1.43)  ≥20: 1.00 (reference) | 1, 2, 4, 5, 9, 11, 33 |
| Kebede ea. 2017 | Number of missing teeth continuous | 1.03 (1.01 – 1.04) | 1, 2, 4, 5, 9, 11, 26 |
| Kim ea. 2013 | 0-15, ≥16 teeth | 0-15: OR = 1.34 (0.96 – 1.88)  ≥16: OR = 1.00 (reference) | 1, 2, 3, 4, 5, 9, 19, 32 |
| LaMonte ea. 2017 | Dentate/edentulous | Edentulous: 1.17 (1.02 – 1.33)  Dentate: 1.00 (reference) | 1, 3, 4, 5, 6, 7, 8, 9, 10, 11, 16, 26 |
| Lee ea. 2019 | 0, 1-4, 5-14, 15-27, 28 missing teeth  Number of missing teeth continuous | 28: 1.62 (1.49 – 1.77)  15-27: 1.53 (1.46 – 1.61)  5-14: 1.28 (1.25 – 1.32)  1-4: 1.11 (1.08 – 1.14)  0: 1.00 (reference)  Continuous: 1.022 (1.020 – 1.023) | 1, 2, 4, 5, 6, 7, 8, 9, 10, 11, 22, 49 |
| Li ea. 2010 | 0, 1-21, ≥22 teeth | 0: 1.48 (1.24 – 1.78)  1-21: 1.24 (1.05 – 1.46)  ≥22: 1.00 (reference) | 1, 2, 4, 5, 6, 7, 8, 9, 10, 11, 19, 22, 24, 25, 35, 36 |
| Liljestrand ea. 2015 | 0-1, 2-4, 5-8, 9-31, 32 missing teeth | 32: 1.68 (1.26 – 2.24)  9-31: 1.37 (1.05 – 1.79)  5-8: 0.90 (0.66 – 1.24)  2-4: 0.88 (0.66 – 1.19)  0-1: 1.00 (reference) | 1, 2, 4, 5, 6, 7, 8, 19, 25 |
| Matsuyama ea. 2017 | 1-9, 10-19, ≥20 teeth | 1-9: 0.80 (0.70 – 0.91)  10-19: 0.71 (0.62 – 0.82)  ≥20: 0.58 (0.50 – 0.68) | 1, 4, 5, 9, 10, 11, 31, 34, 35, 51, 52 |
| Morita ea. 2006 | <20, ≥20 teeth | <20: 2.71 (1.05 – 7.05)  ≥20: 1.00 (reference) | 5, 10 |
| Nomura ea. 2020 | Dentate/edentulous  Number of remaining teeth continuous | Edentulous: 1.09 (0.76 – 1.30)  Dentate: 1.00 (reference)  Continuous: 1.00 (0.99 – 1.02) | No adjustments |
| Osterberg ea. 2007 | Number of remaining teeth continuous | 0.86 (0.81 – 0.92) | 2, 4, 5, 9, 11, 19, 24, 32, 35 |
| Osterberg ea. 2008 | 0, 1-9, 10-19, 20-32 teeth  Number of remaining teeth continuous | *Men:*  20-32: 0.64 (0.48 – 0.84)  10-19: 0.74 (0.58 – 0.94)  1-9: 1.02 (0.82 – 1.27)  0: 1.00 (reference)  *Women:*  20-32: 0.70 (0.51 – 0.96)  10-19: 0.94 (0.74 – 1.20)  1-9: 0.81 (0.61 – 1.07)  0: 1.00 (reference)  Continuous: 0.98 (0.97 – 0.99)  *Both men and women:*  Continuous: 0.99 (0.98 – 0.99) | Men: 19, 35, 39, 41  Women: 5, 6, 25, 35  Both men and women: 2, 5, 6, 19, 35 |
| Padilha ea. 2008 | 0, 1-19, ≥20 teeth | 0: 1.76 (1.04 – 2.98)  1-19: 2.17 (1.50 – 3.13)  ≥20: 1.00 (reference) | 1, 2, 5, 6, 9, 11, 19, 23, 27, 28, 29, 32, 35, 41 |
| Paganini-Hill ea. 2011 | 0, 1-15, 16-25, 26-32 teeth | Men:  0: 1.18 (1.00 – 1.39)  1-15: 1.21 (1.05 – 1.40)  16-25: 1.03 (0.91 – 1.17)  26-32: 1.00 (reference)  Women:  0: 1.21 (1.07 – 1.37)  1-15: 1.17 (1.06 – 1.30)  16-25: 0.97 (0.89 – 1.06)  26-32: 1.0 (reference) | 1, 5, 6, 8, 9, 11, 19, 32, 44 |
| Qi ea. 2020 | Number of missing teeth continuous | Continuous: 1.01 (1.00 – 1.02) | 1, 2, 4, 5, 6, 9, 8, 10 |
| Ragnarsson ea. 2004 | Dentate/edentulous  Number of remaining teeth continuous | Edentulous: 1.15 (0.94 – 1.50)  Dentate: 1.00 (reference)  Continuous: 0.99 (0.98 – 1.00) | 1, 2, 4, 5, 7, 8 |
| Schwahn ea. 2013 | 0, 1-9, 10-19 teeth | 10-19: 0.79 (0.60 – 1.05)  1-9: 0.91 (0.71 – 1.16)  0: 1.00 (reference) | 1, 2, 4, 5, 6, 8, 9, 10, 11, 12, 26 |
| Shimazaki ea. 2001 | Edentulous not using dentures, edentulous using dentures, 1-19 teeth not using dentures, 1-19 teeth using dentures, ≥20 teeth | Edentulous not using dentures: OR = 1.8 (1.1 – 2.8)  Edentulous using dentures: OR = 1.3 (0.8 – 2.4)  1-19 not using dentures: OR = 1.5 (0.9 – 2.4)  1-19 using dentures: OR = 1.3 (0.8 – 2.0)  ≥ 20: OR = 1.00 (reference) | 1, 2, 19, 32, 36, 39 |
| Soikkonen ea. 2000 | Dentate/ edentulous | Edentulous: N=22 deaths/123  Dentate: N=32 deaths/169 | No adjustments |
| Tu ea. 2007 | Number of missing teeth continuous | 1.01 (1.00 – 1.02) | 1, 2, 4, 5, 8, 9 |
| Vedin ea. 2016 | 0, 1-14, 15-19, 20-25, 26-32 teeth | 0: 1.81 (1.50 – 2.20)  1-14: 1.56 (1.35 – 1.80)  15-19: 1.35 (1.22 – 1.48)  20-25: 1.16 (1.11 – 1.22)  26-32: 1.00 (reference) | 1, 2, 4, 5, 6, 7, 8, 9, 10, 11, 19, 20, 22 |
| Vogtmann ea. 2017 | Number of missing teeth in quintiles | Q5 (more teeth missing than expected): 1.43 (1.28 – 1.61)  Q4: 1.22 (1.11 – 1.35)  Q3 (teeth missing as expected): 1.24 (1.11 – 1.38)  Q2: 1.07 (0.95 – 1.20)  Q1 (fewer teeth missing than expected): 1.00 (reference) | 1, 2, 3, 4, 5, 53 |
| Watt ea. 2012 | Edentulous, natural teeth and dentures, natural teeth only | Edentulous: 1.30 (1.73 – 2.82)  Natural + dentures: 0.98 (0.85 – 1.14)  Natural teeth only: 1.00 (reference) | 1, 2, 4, 5, 6, 8, 9, 10, 11, 12, 35 |
| Yuan ea. 2020 | 0, 1-9, 10-19, ≥20 teeth | 0: 1.35 (1.26 – 1.44)  1-9: 1.23 (1.15 – 1.31)  10-19: 1.14 (1.06 – 1.23)  ≥20: 1.00 (reference) | 1, 2, 4, 5, 6, 8, 9, 10, 11, 16, 19, 31, 50, 51 |

Abbreviations: RR, Risk Ratio; HR, Hazard Ratio; OR, Odds Ratio; 95%-CI, 95%-Confidence Interval; Q, quintile

Covariables: (1) age; (2) sex; (3) race or ethnicity; (4) socioeconomic status (income and/or education, profession, place of residence, living situation, husband’s occupation, number of family members living together, household income or wealth index); (5) smoking status or smoking history; (6) diabetes (hemoglobin A1c/fasting serum glucose/plasma glucose/glycemia or medical history of diabetes); (7) hyperlipidemia (low-density lipoprotein cholesterol and/or high-density lipoprotein cholesterol and/or triglycerides or serum total cholesterol level or dyslipoproteinemia or dyslipidemia or cholesterol); (8) hypertension (systolic and/or diastolic blood pressure) or history of hypertension or resting heart rate; (9) body mass index or waist/hip ratio or obesity or height and weight or abdominal skinfold thickness; (10) alcohol consumption or drinking status; (11) physical activity/function or time spent walking daily or walking speed or activity of daily living or exercise; (12) marital status or partnership; (13) microalbuminuria or serum albumin; (14) C-reactive protein; (15) fibrinogen; (16) diet; (17) IL-6; (18) statin intake/lipid lowering drugs; (19) ACVD/history of ACVD (myocardial infarction, ischemic heart disease, cerebrovascular disease, stroke, angina pectoris, transitory ischemic attack, cardiac disease, peripheral arterial disease, baseline degree of carotid stenosis, diagnosis of heart failure, macrovascular or microvascular disease, circulation disease); (20) family history of ACVD; (21) menopausal status and hormonal use; (22) renal disease or creatinine level or estimated glomerular filtration rate; (23) papillary bleeding score or bone loss or probing depth or average Periodontal Index or average Gingival Index or periodontal disease; (24) dependent living or requiring assistance with daily activities or I-ADL (Instrumental Activities of Daily Living); (25) hypertension medication; (26) frequency of dental visits or dental checkup or regular dental prophylaxis/cleaning; (27) oral hygiene or frequency or brushing teeth and/or use of floss or interdental brushes; (28) missing teeth or tooth loss or number of teeth or difficulty of chewing; (29) DMFT index (decayed, missing, filled teeth) or dental caries; (30) family history of hypertension; (31) co-morbidities (number of chronic diseases or osteoarthritis, activity limitation/fatigue in daily activities, rheumatoid arthritis, diabetes mellitus, ischemic heart disease, cerebrovascular disease, cancer, asthma, chronic bronchitis, emphysema, lung cancer, respiratory diseases, FEV 1.0 (Tiffeneau-Pinelli index), musculo-skeletal disease or present illness, malignancy history); (32) health insurance/dental insurance; (33) age at first birth/parity; (34) self-rated health or quality of life; (35) psychological distress score or Mini Mental State Exam Score or social activity or physical-mental health status or depressive symptoms or geriatric depression scale score; (36) energy intake; (37) protein intake; (38) tiredness or physical status; (39) number of drugs; (40) white blood cell count; (41) hemoglobin; (42) number of detected different bacterial species per individual; (43) caffeine intake; (44) multivitamin supplement use and/or Vitamin E use and/or mineral supplements and/or vitamin D intake and/or calcium supplements; (45) intake of antibiotics and/or NSAID (Non-Steroidal Anti-Inflammatory Drugs) and/or HRT (Hormone Replacement Therapy); (46) number of siblings and/or early-life exposures, and familial factors; (47) aspartate aminotransferase, alanine aminotransferase, gamma-glutamyl transferase; (48) cooperativeness with nursing staff and/or dental personnel; (49) medical history of pneumonia or chronic pulmonary disease; (50) cognitive impairment; (51) denture use; (52) falling experience; (53) opium use; (54) history of cancer; (55) liver panel; (56) proteinuria; (57) aspirin use
