# Supplementary material for: Lower Number of Teeth Is Related to Higher Risks for ACVD and Death—Systematic Review and Meta-Analyses of Survival Data
Source: Front Cardiovasc Med. 2021 May 7;8:621626. doi: 10.3389/fcvm.2021.621626 (PMC8138430; doi:10.3389/fcvm.2021.621626)
Supplement: Supplementary file 10 [file Table_3.docx]

Supplementary Table 3: Meta-analyses and subgroup analyses for cumulative incidence of ACVD-related events (morbidity or mortality) and All-Cause Mortality

| **All patients** | |  | | | **ACVD at baseline** | | | | **Follow-up rate** | | | | **Approaches to count the number of teeth** | | | | |
| --- | --- | --- | --- | --- | --- | --- | --- | --- | --- | --- | --- | --- | --- | --- | --- | --- | --- |
| **RR (95%-CI)** | **Number of studies** | **I^2^** | **Number of patients** | **Number of events** | **ACVD adjusted or excluded vs. no adjusted or excluded, or unknown** | **Number of studies** | **I^2^** | **Number of patients** | **High vs. Low** | **Number of studies** | **I^2^** | **Number of patients** | **Clinical vs. self-reported** | **Number of studies** | **I^2^** | **Number of patients** | |
| **0 teeth vs. 1-32 teeth (ref.) (ACVD)** | | | | |  |  |  |  |  |  |  |  |  |  |  |  | |
| 2.93 (1.92 – 4.50) | 10 | 98.8% | 4,697,021 | 95,988 | 2.92 (1.77 – 4.82)  3.10 (1.96 – 4.88) | 8  2 | 99.1%  78.7% | 4,683,677  13,344 | 2.88 (1.80 – 4.61)  3.49 (2.93 – 4.17) | 9  1 | 99.0%  N.A. | 4,687,690  9,331 | 3.30 (1.71 – 6.38)  2.59 (1.94 – 3.46) | 5  5 | 98.9%  94.2% | 4,427,360  269,661 | |
| **0 teeth vs. 1-32 teeth (ref.) (ACM)** | | |  | |  |  |  |  |  |  |  |  |  |  |  |  | |
| 1.94 (1.19 – 3.15) | 14 | 99.5% | 4,711,667 | 81,809 | 2.17 (1.04 – 4.53)  1.73 (1.23 – 2.43) | 8  6 | 99.7%  93.5% | 4,641,316  70,351 | 2.12 (1.28 – 3.52)  1.13 (1.05 1.21) | 12  2 | 99.3%  32.6% | 4,708,524  3,143 | 1.73 (0.83 – 3.58)  2.53 (1.92 – 3.33) | 10  4 | 99.7%  95.2% | 4,466,319  245,348 | |
| **0-19 teeth vs. 20-32 teeth (ref.) (ACVD)** | | | | |  |  |  |  |  |  |  |  |  |  |  |  | |
| 1.98 (1.86 – 2.10) | 4 | 0% | 191,129 | 4,369 | 1.98 (1.86 – 2.10)  N.A. | 4  0 | 0%  N.A. | 191,129  0 | 1.98 (1.86 – 2.10)  N.A. | 4  0 | 0%  N.A. | 191,129  0 | N.A.  1.98 (1.86 – 2.10) | 0  4 | N.A.  0% | 0  191,129 | |
| **0-19 teeth vs. 20-32 teeth (ref.) (ACM)** | | | | |  |  |  |  |  |  |  |  |  |  |  | |  |
| 1.84 (1.52 – 2.23) | 11 | 95.0% | 267,575 | 11,543 | 2.06 (1.65 – 2.57)  1.40 (1.07 – 1.83) | 8  3 | 94.1%  85.6% | 210,915  56,660 | 1.95 (1.59 – 2.40)  1.38 (1.19 – 1.61) | 9  2 | 94.8%  31.6% | 264,432  3,143 | 1.65 (1.42 – 1.93)  2.05 (1.58 – 2.66) | 6  5 | 79.4%  94.3% | | 60,411  207,164 |
| **0 teeth vs. 1-19 teeth vs. 20-32 teeth (ref.) (ACVD)** | | | | |  |  |  |  |  |  |  |  |  |  |  | |  |
| 0 vs. 20-32: 2.65 (1.77 – 3.98)  1-19 vs. 20-32: 1.69 (1.13 – 2.53) | 2 | 59.9% | 175,476 | 4,030 | 0 vs. 20-32: 2.65 (1.77 – 3.98)  1-19 vs. 20-32: 1.69 (1.13 – 2.53)  0 vs. 20-32: N.A.  1-19 vs. 20-32: N.A. | 2  0 | 59.9%  N.A. | 175,476  0 | 0 vs. 20-32: 2.65 (1.77 – 3.98)  1-19 vs. 20-32: 1.69 (1.13 – 2.53)  0 vs. 20-32: N.A.  1-19 vs. 20-32: N.A. | 2  0 | 59.9%  N.A. | 175,476  0 | 0 vs. 20-32: N.A.  1-19 vs. 20-32: N.A.  0 vs. 20-32: 2.65 (1.77 – 3.98)  1-19 vs. 20-32: 1.69 (1.13 – 2.53) | 0  2 | N.A.  59.9% | | 0  175,476 |
| **0 teeth vs. 1-19 teeth vs. 20-32 teeth (ref.) (ACM)** | | | | |  |  |  |  |  |  |  |  |  |  |  | |  |
| 0 vs. 20-32: 2.27 (1.82 – 2.83)  1-19 vs. 20-32: 1.76 (1.41 – 2.19) | 8 | 93.9% | 235,887 | 8,244 | 0 vs. 20-32: 2.34 (1.76 – 3.10)  1-19 vs. 20-32: 1.84 (1.39 – 2.43)  0 vs. 20-32: 2.09 (1.85 – 2.36)  1-19 vs. 20-32: 1.51 (1.40 – 1.62) | 6  2 | 94.5%  0% | 179,345  56,542 | 0 vs. 20-32: 2.66 (2.04 – 3.46)  1-19 vs. 20-32: 1.92 (1.48 – 2.48)  0 vs. 20-32: 1.42 (1.24 – 1.62)  1-19 vs. 20-32: 1.32 (1.16 – 1.52) | 6  2 | 92.1%  43.0% | 232,744  3,143 | 0 vs. 20-32: 1.85 (1.59 – 2.14)  1-19 vs. 20-32: 1.59 (1.38 – 1.83)  0 vs. 20-32: 3.93 (3.02 – 5.12)  1-19 vs. 20-32: 2.29 (1.77 – 2.97) | 6  2 | 77.8%  71.3% | | 60,411  175,476 |
| **0-10 teeth vs. 11-16 teeth vs. 17-24 teeth vs. 25-32 teeth (ref.) (ACVD)** | | | | |  |  |  |  |  |  |  |  |  |  |  | |  |
| 0-10 vs. 25-32: 2.65 (1.84 – 3.81)  11-16 vs. 25-32: 2.27 (1.55 – 3.32)  17-24 vs. 25-32: 1.73 (1.22 – 2.45) | 4 | 86.7% | 230,974 | 3,646 | 0-10 vs. 25-32: 2.65 (1.84 – 3.81)  11-16 vs. 25-32: 2.27 (1.55 – 3.32)  17-24 vs. 25-32: 1.73 (1.22 – 2.45)  0-10 vs. 25-32: N.A.  11-16 vs. 25-32: N.A.  17-24 vs. 25-32: N.A. | 4  0 | 86.7%  N.A. | 230,974  0 | 0-10 vs. 25-32: 2.65 (1.84 – 3.81)  11-16 vs. 25-32: 2.27 (1.55 – 3.32)  17-24 vs. 25-32: 1.73 (1.22 – 2.45)  0-10 vs. 25-32: N.A.  11-16 vs. 25-32: N.A.  17-24 vs. 25-32: N.A. | 4  0 | 86.7%  N.A. | 230,974  0 | 0-10 vs. 25-32: N.A.  11-16 vs. 25-32: N.A.  17-24 vs. 25-32: N.A.  0-10 vs. 25-32: 2.65 (1.84 – 3.81)  11-16 vs. 25-32: 2.27 (1.55 – 3.32)  17-24 vs. 25-32: 1.73 (1.22 – 2.45) | 0  4 | N.A.  86.7% | | 0  230,974 |
| **0-10 teeth vs. 11-16 teeth vs. 17-24 teeth vs. 25-32 teeth (ref.) (ACM)** | | | | |  |  |  |  |  |  |  |  |  |  |  | |  |
| No data | 0 | N.A. | 0 | 0 | N.A. | 0 | N.A. | 0 | N.A. | 0 | N.A. | 0 | N.A. | 0 | N.A. | | 0 |

Abbreviations: RR, Risk Ratio; 95%-CI, 95%-Confidence Interval; vs., versus; ref., reference; I^2^, I-square for heterogeneity; ACVD, Atherosclerotic Cardiovascular Disease; ACM, All-Cause Mortality; N.A., Not Applicable
