# Supplementary material for: Lower Number of Teeth Is Related to Higher Risks for ACVD and Death—Systematic Review and Meta-Analyses of Survival Data
Source: Front Cardiovasc Med. 2021 May 7;8:621626. doi: 10.3389/fcvm.2021.621626 (PMC8138430; doi:10.3389/fcvm.2021.621626)
Supplement: Supplementary file 11 [file Table_4.docx]

Supplementary Table 4: Meta-analyses and subgroup analyses for incidence density of ACVD-related events (morbidity or mortality) and All-Cause Mortality

| **All patients** | |  | | | | **ACVD at baseline** | | | | **Follow-up rate** | | | | **Approaches to count the number of teeth** | | | | |
| --- | --- | --- | --- | --- | --- | --- | --- | --- | --- | --- | --- | --- | --- | --- | --- | --- | --- | --- |
| **HR (95%-CI)** | **Number of studies** | **I^2^** | **Number of patients** | | **Number of events** | **ACVD adjusted or excluded vs. no adjusted or excluded, or unknown** | **Number of studies** | **I^2^** | **Number of patients** | **High vs. Low** | **Number of studies** | **I^2^** | **Number of patients** | **Clinical vs. self-reported** | **Number of studies** | **I^2^** | **Number of patients** | |
| **0 teeth vs. 1-32 teeth (ref.) (ACVD)** | | | | | |  |  |  |  |  |  |  |  |  |  |  |  | |
| Crude: 1.89 (1.80 – 1.99) | 3 | 0% | 98,122 | >3,589* | | 1.89 (1.80 – 1.99)  N.A. | 3  0 | 0%  N.A. | 98,122  0 | 1.89 (1.80 – 1.99)  1.90 (1.06 – 3.39) | 2  1 | 0%  N.A. | 98,001  121 | 1.90 (1.06 – 3.39)  1.89 (1.80 – 1.99) | 1  2 | N.A.  0% | 121  98,001 | |
| Adjusted by age/sex: 1.49 (1.39 – 1.58) | 4 | 0% | 44,937 | >116* | | 1.49 (1.37 – 1.61)  1.88 (1.15 – 3.07) | 3  1 | 2%  N.A. | 42,324  2,613 | 1.59 (1.32 – 1.93)  1.53 (0.85 – 2.74) | 3  1 | 32%  N.A. | 44,816  121 | 1.84 (1.39 – 2.43)  1.47 (1.38 – 1.57) | 3  1 | 0%  N.A. | 3,937  41,000 | |
| Multivariate: 1.21 (1.07 – 1.36) | 5 | 35% | 101,938 | >3,705* | | 1.20 (1.05 – 1.37)  1.46 (0.88 – 2.43) | 4  1 | 46%  N.A. | 99,325  2,613 | 1.21 (1.06 – 1.39)  1.40 (0.76 – 2.59) | 4  1 | 49%  N.A. | 101,817  121 | 1.55 (1.17 – 2.07)  1.16 (1.02 – 1.31) | 3  2 | 0%  60% | 3,937  98,001 | |
| **0 teeth vs. 1-32 teeth (ref.) (ACM)** | | | | | |  |  |  |  |  |  |  |  |  |  |  |  | |
| Crude: 1.73 (1.41 – 2.13) | 5 | 91% | 99,303 | >3,816* | | 2.00 (1.75 – 2.27)  0.99 (0.76 – 1.30) | 4  1 | 74%  N.A. | 98,695  608 | 1.71 (1.37 – 2.15)  1.85 (1.21 – 2.82) | 4  1 | 93%  N.A. | 99,182  121 | 1.41 (0.99 – 2.01)  2.19 (2.09 – 2.29) | 3  2 | 80%  0% | 13,02  98,001 | |
| Adjusted by age/sex: 1.56 (1.48 – 1.65) | 3 | 0% | 43,734 | >405* | | 1.57 (1.49 – 1.67)  1.37 (1.10 – 1.72) | 2  1 | 0%  N.A. | 41,121  2,613 | 1.54 (1.39 – 1.70)  1.58 (1.03 – 2.41) | 2  1 | 25%  N.A. | 43,613  121 | 1.41 (1.16 – 1.73)  1.57 (1.49 – 1.67) | 2  1 | 0%  N.A. | 2,734  41,000 | |
| Multivariate: 1.30 (1.21 – 1.40) | 7 | 20% | 102,254 | >4,328* | | 1.30 (1.18 – 1.43)  1.31 (1.10 – 1.56) | 5  2 | 36%  21% | 99,106  3,148 | 1.28 (1.17 – 1.40)  1.43 (1.19 – 1.73) | 5  2 | 39%  0% | 101,598  656 | 1.34 (1.19 – 1.50)  1.27 (1.10 – 1.45)) | 5  2 | 0%  71% | 4,253  98,001 | |
| **0-19 teeth vs. 20-32 teeth (ref.) (ACVD)** | | | | | |  |  |  |  |  |  |  |  |  |  |  |  | |
| Crude: 2.27 (1.50 – 3.43) | 1 | N.A. | 4,380 | 104 | | 2.27 (1.50 – 3.43)  N.A. | 1  0 | N.A.  N.A. | 4,380  0 | 2.27 (1.50 – 3.43)  N.A. | 1  0 | N.A.  N.A. | 4,380  0 | N.A.  2.27 (1.50 – 3.43) | 0  1 | N.A.  N.A. | 0  4,380 | |
| Adjusted by age/sex: 1.29 (1.08 – 1.53) | 3 | 58% | 186,749 | 4,265* | | 1.29 (1.08 – 1.53)  N.A. | 3  0 | 58%  N.A. | 186,749  0 | 1.29 (1.08 – 1.53)  N.A. | 3  0 | 58%  N.A. | 186,749  0 | N.A.  1.29 (1.08 – 1.53) | 0  3 | N.A.  58% | 0  186,749 | |
| Multivariate: 1.19 (1.13 – 1.26) | 4 | 0% | 191,129 | 4,369 | | 1.19 (1.13 – 1.26)  N.A. | 4  0 | 0%  N.A. | 191,129  0 | 1.19 (1.13 – 1.26)  N.A. | 4  0 | 0%  N.A. | 191,129  0 | N.A.  1.19 (1.13 – 1.26) | 0  4 | N.A.  0% | | 0  191,129 |
| **0-19 teeth vs. 20-32 teeth (ref.) (ACM)** | | | | | |  |  |  |  |  |  |  |  |  |  |  | |  |
| Crude: 2.47 (2.40 – 2.54) | 2 | 0% | 36,379 | 26,007 | | 2.47 (2.40 – 2.54)  N.A. | 2  0 | 0%  N.A. | 36,379  0 | 2.47 (2.40 – 2.54)  N.A. | 2  0 | 0%  N.A. | 36,379  0 | 2.62 (1.45 – 4.72)  2.47 (2.40 – 2.54) | 1  1 | N.A.  N.A. | | 226  36,153 |
| Adjusted by age/sex: 1.42 (1.15 – 1.75) | 5 | 97% | 211,064 | 10,166 | | 1.42 (1.15 – 1.75)  N.A. | 5  0 | 97%  N.A. | 211,064  0 | 1.42 (1.15 – 1.75)  N.A. | 5  0 | 97%  N.A. | 211,064  0 | N.A.  1.42 (1.15 – 1.75) | 0  5 | N.A.  97% | | 0  211,064 |
| Multivariate: 1.29 (1.21 – 1.37) | 10 | 78% | 305,970 | 40,144 | | 1.27 (1.18 – 1.37)  1.40 (1.12 – 1.76) | 7  3 | 83%  70% | 249,310  56,660 | 1.29 (1.21 – 1.37)  N.A. | 10  0 | 78%  N.A. | 305,970  0 | 1.51 (1.16 – 1.96)  1.25 (1.17 – 1.34) | 3  7 | 85%  78% | | 57,042  248,928 |
| **Number of lost teeth (ACVD)** | | | | | |  |  |  |  |  |  |  |  |  |  |  | |  |
| Crude: 1.01 (0.99 – 1.03) | 1 | N.A. | 1,385 | | 110 | N.A.  1.01 (0.99 – 1.03) | 0  1 | N.A.  N.A. | 0  1,385 | 1.01 (0.99 – 1.03)  N.A. | 1  0 | N.A.  N.A. | 1,385  0 | 1.01 (0.99 – 1.03)  N.A. | 1  0 | N.A.  N.A. | | 1,385  0 |
| Adjusted by age/sex: 1.01 (1.00 – 1.03) | 7 | 79% | 45,46,574 | | 86,727 | 1.02 (0.99 – 1.04)  1.02 (1.00 – 1.04) | 5  2 | 85%  20% | 4,542,576  3,998 | 1.01 (1.00 – 1.03)  N.A. | 7  0 | 79%  N.A. | 4,546,574  0 | 1.02 (1.02 – 1.02)  1.13 (0.97 – 1.32) | 3  4 | 0%  78% | | 4,408,968  137,606 |
| Multivariate: 1.02 (1.01 – 1.03) | 13 | 75% | 4,639,644 | | >93,192* | 1.02 (1.00 – 1.03)  1.03 (1.00 – 1.06) | 7  6 | 72%  82% | 4,582,345  57,299 | 1.02 (1.01 – 1.03)  N.A. | 13  0 | 75%  N.A. | 4,639,644  0 | 1.02 (1.00 – 1.04)  1.02 (0.99 – 1.06) | 7  6 | 78%  77% | | 4,462,269  177,375 |
| **Number of lost teeth (ACM)** | | | | | |  |  |  |  |  |  |  |  |  |  |  | |  |
| Crude: 1.02 (1.00 – 1.03) | 3 | 80% | 2,219 | | >507* | 1.03 (1.01 – 1.05)  1.01 (1.00 – 1.03) | 1  2 | N.A.  63% | 226  1,993 | 1.02 (1.00 – 1.03)  N.A. | 3  0 | 0%  N.A. | 2,219  0 | 1.02 (1.00 – 1.03)  N.A. | 3  0 | 0%  N.A. | | 2,219  0 |
| Adjusted by age/sex: 1.02 (1.01 – 1.04) | 5 | 93% | 4,421,245 | | >69,871* | 1.03 (1.01 – 1.04)  1.02 (1.01 – 1.03) | 3  2 | 96%  0% | 4,417,247  3,998 | 1.02 (1.01 – 1.04)  N.A. | 5  0 | 93%  N.A. | 4,421,245  0 | 1.02 (1.01 – 1.03)  1.08 (0.95 – 1.23) | 3  2 | 79%  94% | | 4,408,968  12,277 |
| Multivariate: 1.02 (1.01 – 1.03) | 11 | 89% | 4,467,321 | | >82,450* | 1.02 (1.01 – 1.03)  1.03 (1.01 – 1.05) | 6  5 | 91%  88% | 4,419,658  47,663 | 1.02 (1.01 – 1.03)  1.02 (1.01 – 1.02) | 10  1 | 90%  N.A. | 4,465,940  1,381 | 1.02 (1.01 – 1.03)  1.05 (0.95 – 1.17) | 9  2 | 84%  87% | | 4,455,044  12,277 |

*The actual number of the events may be larger than the presented data because some studies included in the meta-analysis did not report the number of the events. Therefore, those studies were not counted in the total number of the events.

Abbreviations: RR, Risk Ratio, 95%-CI, 95%-Confidence Interval; vs., versus; ref., reference; I^2^, I-square for heterogeneity; ACVD, Atherosclerotic Cardiovascular Disease; ACM, All-Cause Mortality; N.A., Not Applicable
