# Supplementary material for: Lower Number of Teeth Is Related to Higher Risks for ACVD and Death—Systematic Review and Meta-Analyses of Survival Data
Source: Front Cardiovasc Med. 2021 May 7;8:621626. doi: 10.3389/fcvm.2021.621626 (PMC8138430; doi:10.3389/fcvm.2021.621626)
Supplement: Supplementary file 12 [file Table_5.docx]

Supplementary Table 5: Cumulative meta-analysis for cumulative incidence of ACVD-related events (morbidity or mortality) and All-Cause Mortality


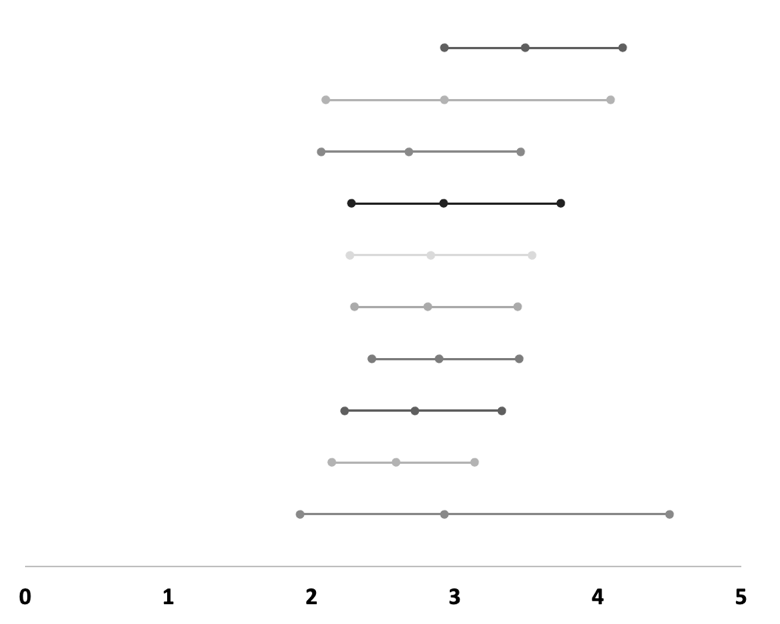


| **0 teeth vs. 1-32 teeth (ref.) (ACVD)** | | | | |
| --- | --- | --- | --- | --- |
| **Author/Year** | **Cumulative events/sample size in index group(s)** | **Cumulative events/sample size in reference group** | **RR (95%-CI)** | **I^2^** |
| Morrison ea. 1999 | 258/2,445 | 208/6,886 | 3.49 (2.93 – 4.17) | N.A. |
| +Wu ea. 2000 | 588/4,627 | 681/14,666 | 2.93 (2.10 – 4.09) | 89% |
| +Heitmann ea. 2008 | 669/4,907 | 991/17,010 | 2.68 (2.07 – 3.46) | 85% |
| +Watt ea. 2012 | 948/8,147 | 1,210/26,641 | 2.92 (2.28 – 3.74) | 88% |
| +Janket ea. 2014 | 983/8,264 | 1,255/26,997 | 2.83 (2.27 – 3.54) | 85% |
| +Ando ea. 2014 | 1,016/9,877 | 1,303/33,163 | 2.81 (2.30 – 3.44) | 81% |
| +Oluwagbemigun ea. 2015 | 1,108/11,574 | 1,669/55,779 | 2.89 (2.42 – 3.45) | 79% |
| +Joshy ea. 2016 | 1,496/20,371 | 5,230/214,679 | 2.72 (2.23 – 3.33) | 89% |
| +LaMonte ea. 2017 | 1,891/23,713 | 8,651/268,338 | 2.59 (2.14 – 3.14) | 92% |
| +Lee ea. 2019 | 2,369/26,630 | 93,619/4,670,391 | 2.93 (1.92 – 4.50) | 99% |

| **0-19 teeth vs. 20-32 teeth (ref.) (ACVD)** | | | | |
| --- | --- | --- | --- | --- |
| **Author/Year** | **Cumulative events/sample size in index group(s)** | **Cumulative events/sample size in reference group** | **RR (95%-CI)** | **I^2^** |
| Aida ea. 2011 | 87/3,085 | 17/1,295 | 2.15 (1.28 – 3.60) | N.A. |
| +Ando ea. 2014 | 150/8,069 | 35/4,090 | 2.05 (1.42 – 2.96) | 0% |
| +Joshy ea. 2016 | 1,948/58,302 | 2,186/121,554 | 1.96 (1.84 – 2.08) | 0% |
| +Goto ea. 2020 | 2,105/15,850 | 2,264/127,728 | 1.98 (1.86 – 2.10) | 0% |


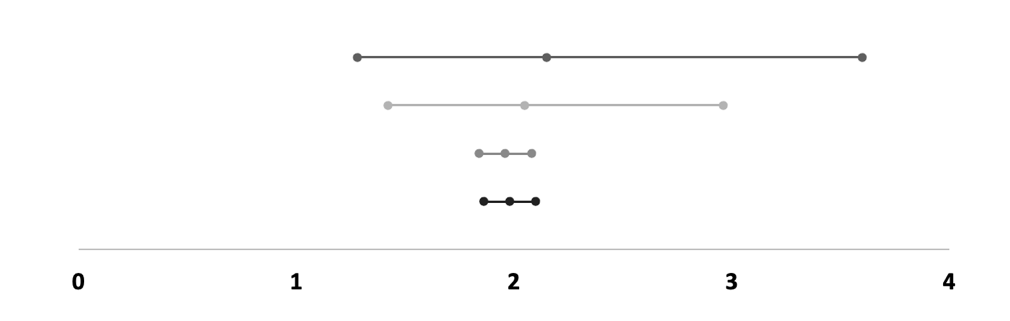


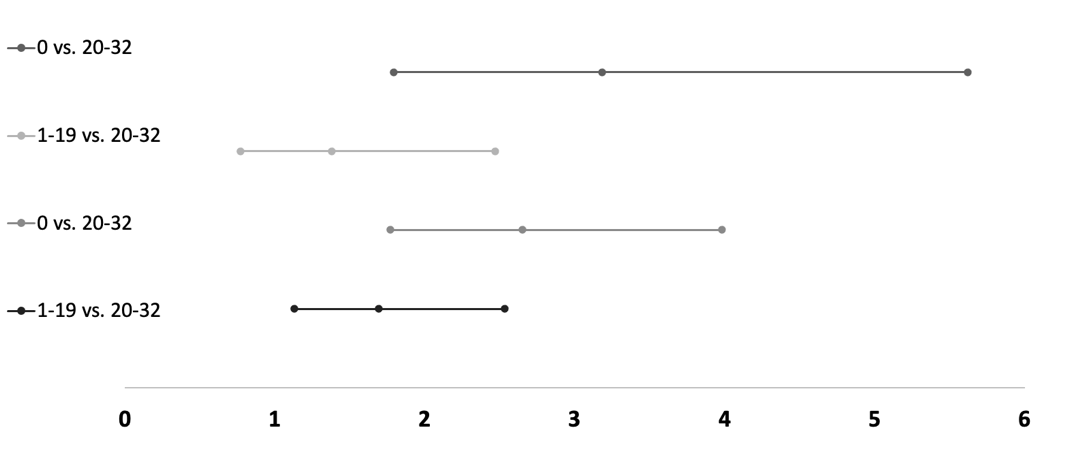


| **0 teeth vs. 1-19 teeth vs. 20-32 teeth (ref.) (ACVD)** | | | | |
| --- | --- | --- | --- | --- |
| **Author/Year** | **Cumulative events/sample size in index group(s)** | **Cumulative events/sample size in reference group** | **RR (95%-CI)** | **I^2^** |
| Ando ea. 2014 | 0: 33/1,613  1-19: 30/3,371 | 18/2,795 | 0 vs. 20-32:  3.18 (1.79 – 5.62)  1-19 vs. 20-32:  1.38 (0.77 – 2.47) | N.A. |
| +Joshy ea. 2016 | 0: 421/10,410  1-19: 1,440/44,807 | 2,169/120,259 | 0 vs. 20-32:  2.65 (1.77 – 3.98)  1-19 vs. 20-32:  1.69 (1.13 – 2.53) | 60% |

| **0-10 teeth vs. 11-16 teeth vs. 17-24 teeth vs. 25-32 teeth (ref.) (ACVD)** | | | | |
| --- | --- | --- | --- | --- |
| **Author/Year** | **Cumulative events/sample size in index group(s)** | **Cumulative events/sample size in reference group** | **RR (95%-CI)** | **I^2^** |
| Joshipura ea. 1996 | 0-10: 57/1,275  11-16: 29/950  17-24: 117/4,815 | 554/37,079 | 0-10 vs. 25-32: 2.99 (2.29 – 3.91)  11-16 vs. 25-32: 2.04 (1.41 – 2.95)  17-24 vs. 25-32: 1.63 (1.34 – 1.98) | N.A. |
| +Joshipura ea. 2003 | 0-10: 85/2,458  11-16: 48/1,853  17-24: 189/9,342 | 784/71,846 | 0-10 vs. 25-32: 3.26 (2.32 – 4.58)  11-16 vs. 25-32: 2.49 (1.69 – 3.66)  17-24 vs. 25-32: 1.95 (1.44 – 2.63) | 57% |
| +Hung ea. 2003 | 0-10: 109/3,782  11-16: 67/2,844  17-24: 254/14,340 | 1,018/109,627 | 0-10 vs. 25-32: 3.16 (2.51 – 3.98)  11-16 vs. 25-32: 2.62 (2.00 – 3.45)  17-24 vs. 25-32: 1.97 (1.64 – 2.36) | 29% |
| +Hung ea. 2004 | 0-10: 353/11,099  11-16: 181/6,673  17-24: 631/30,755 | 2,481/182,447 | 0-10 vs. 25-32: 2.65 (1.84 – 3.81)  11-16 vs. 25-32: 2.27 (1.55 – 3.32)  17-24 vs. 25-32: 1.73 (1.22 – 2.45) | 87% |


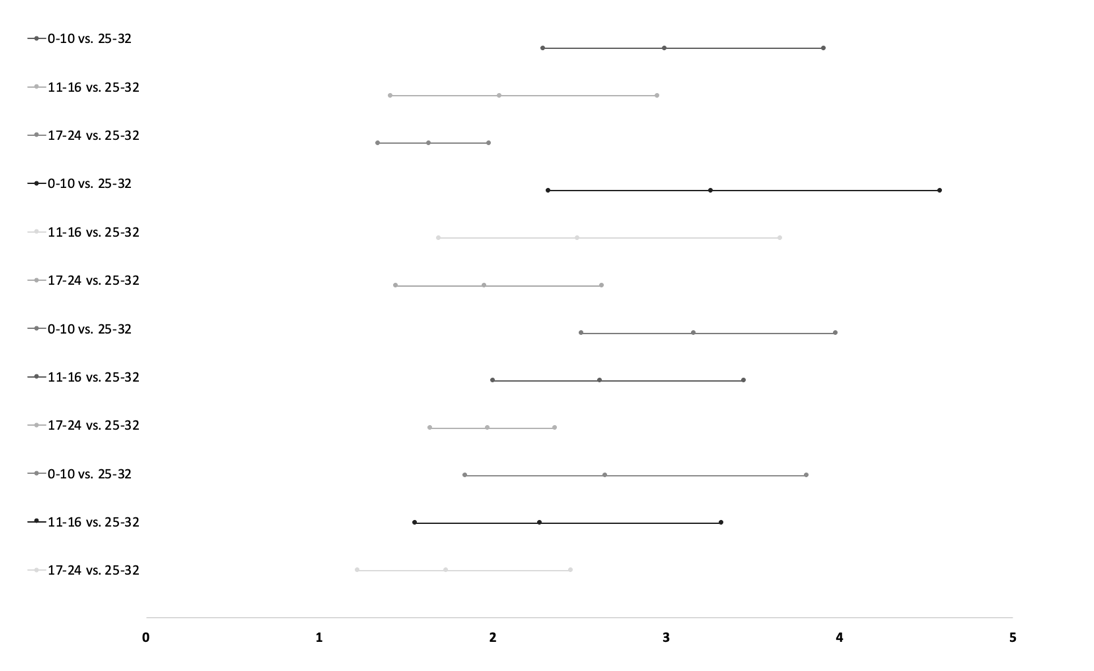


| **0 teeth vs. 1-32 teeth (ref.) (ACM)** | | | | |
| --- | --- | --- | --- | --- |
| **Author/Year** | **Cumulative events/sample size in index group(s)** | **Cumulative events/sample size in reference group** | **RR (95%-CI)** | **I^2^** |
| Soikkonen ea. 2000 | 22/123 | 32/169 | 0.94 (0.58 – 1.54) | N.A. |
| +Shimazaki ea. 2001 | 499/1,032 | 411/1,022 | 1.17 (1.06 – 1.29) | 0% |
| +Hamalainen ea. 2003 | 568/1,125 | 492/1,155 | 1.18 (1.09 – 1.29) | 0% |
| +Osterberg ea. 2008 | 991/1,676 | 1,072/1,985 | 1.13 (1.07 – 1.19) | 0% |
| +Padilha ea. 2008 | 1,009/1,700 | 1,252/2,461 | 1.25 (1.07 – 1.45) | 80% |
| +Watt ea. 2012 | 1,767/4,940 | 1,974/12,092 | 1.47 (0.96 – 2.23) | 99% |
| +Janket ea. 2014 | 1,813/5,057 | 2,052/12,448 | 1.50 (1.03 – 2.21) | 98% |
| +Ando ea. 2014 | 1,959/6,670 | 2,282/18,614 | 1.60 (1.12 – 2.29) | 98% |
| +Hu ea. 2015 | 2,107/7,851 | 5,664/73,084 | 1.65 (1.19 – 2.27) | 98% |
| +Joshy ea. 2016 | 2,358/16,648 | 7,227/231,984 | 1.74 (1.27 – 2.39) | 98% |
| +LaMonte ea. 2017 | 2,753/19,990 | 10,648/285,643 | 1.76 (1.34 – 2.31) | 98% |
| +Lee ea. 2019 | 3,294/22,907 | 78,170/468,7696 | 2.05 (1.21 – 3.50) | 100% |
| +Dewake ea. 2020 | 3,306/22,977 | 78,186/4,687,799 | 1.97 (1.18 – 3.29) | 100% |
| +Hiratsuka ea. 2020 | 3,381/23,124 | 78,428/4,688,543 | 1.94 (1.19 – 3.15) | 100% |


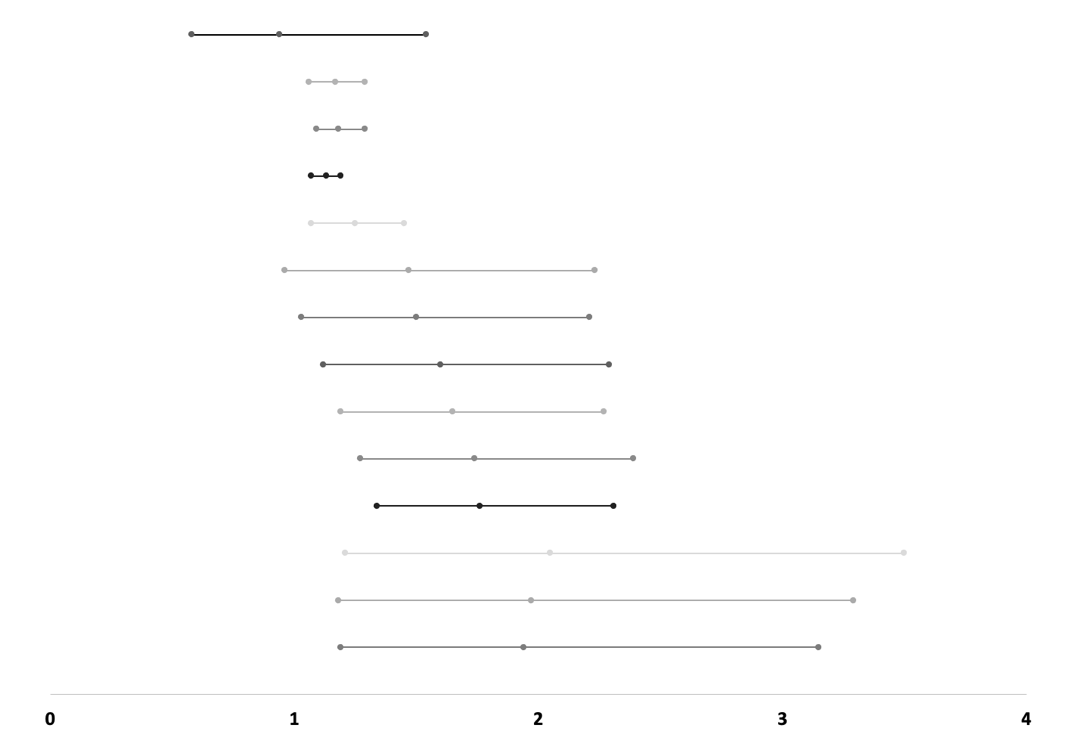


| **0-19 teeth vs. 20-32 teeth (ref.) (ACM)** | | | | |
| --- | --- | --- | --- | --- |
| **Author/Year** | **Cumulative events/sample size in index group(s)** | **Cumulative events/sample size in reference group** | **RR (95%-CI)** | **I^2^** |
| Shimazaki ea. 2001 | 815/1,633 | 41/129 | 1.57 (1.21 – 2.03) | N.A. |
| +Hamalainen ea. 2003 | 959/1,843 | 47/145 | 1.60 (1.26 – 2.04) | 0% |
| +Morita ea. 2006 | 996/1,902 | 86/204 | 1.33 (0.89 – 2.01) | 76% |
| +Osterberg ea. 2008 | 1,870/3,058 | 215/429 | 1.31 (1.05 – 1.63) | 65% |
| +Padilha ea. 2008 | 1,943/3,160 | 340/827 | 1.50 (1.11 – 2.03) | 89% |
| +Hayasaka ea. 2013 | 3,697/17,264 | 711/7,020 | 1.60 (1.23 – 2.09) | 92% |
| +Ando ea. 2014 | 4,001/22,248 | 783/9,815 | 1.69 (1.33 – 2.16) | 91% |
| +Hu ea. 2015 | 4,917/32,396 | 3,397/55,318 | 1.68 (1.40 – 2.00) | 90% |
| +Joshy ea. 2016 | 5,924/82,629 | 4,204/172,782 | 1.79 (1.43 – 2.26) | 96% |
| +Goto ea. 2020 | 6,655/87,728 | 4,571/178,956 | 1.85 (1.51 – 2.28) | 96% |
| +Hiratsuka ea. 2020 | 6,877/88,242 | 4,666/179,333 | 1.84 (1.52 – 2.23) | 95% |


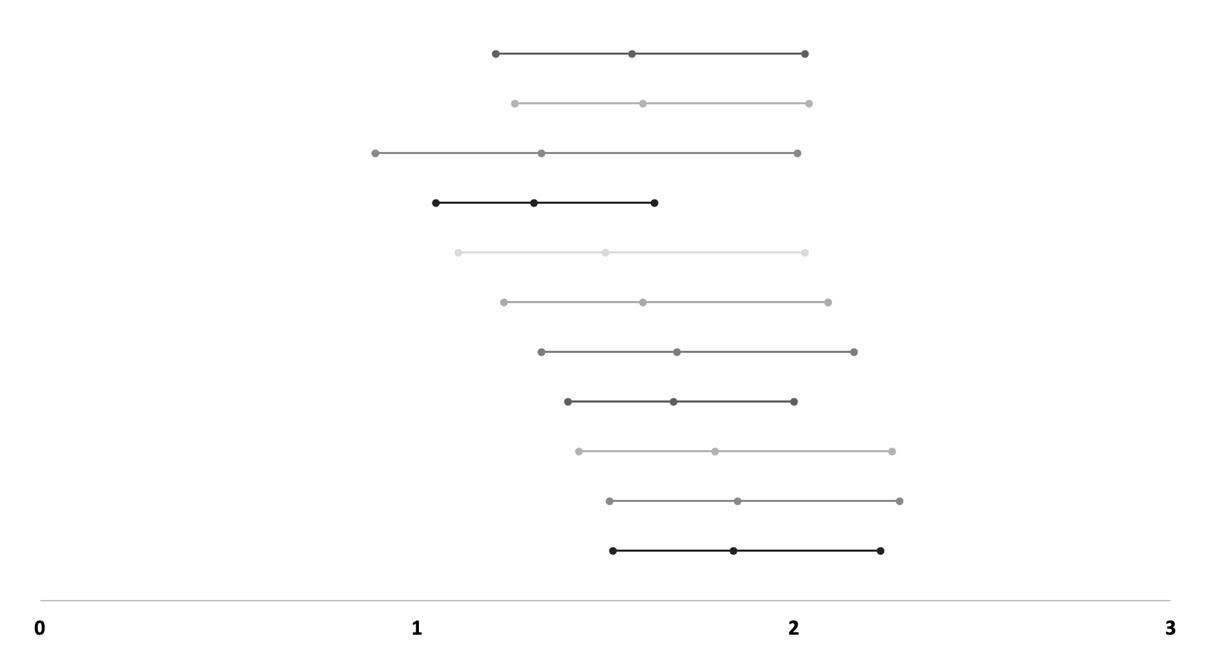


| **0 teeth vs. 1-19 teeth vs. 20-32 teeth (ref.) (ACM)** | | | | |
| --- | --- | --- | --- | --- |
| **Author/Year** | **Cumulative events/sample size in index group(s)** | **Cumulative events/sample size in reference group** | **RR (95%-CI)** | **I^2^** |
| Shimazaki ea. 2001 | 0: 477/909  1-19: 338/724 | 41/129 | 0 vs. 20-32: 1.65 (1.27 – 2.14)  1-19 vs. 20-32: 1.47 (1.13 – 1.91) | N.A. |
| +Hamalainen ea. 2003 | 0: 546/1,002  1-19: 413/841 | 47/145 | 0 vs. 20-32: 1.69 (1.33 – 2.16)  1-19 vs. 20-32: 1.50 (1.17 – 1.91) | 0% |
| +Osterberg ea. 2008 | 0: 969/1,553  1-19: 864/1,446 | 176/370 | 0 vs. 20-32: 1.43 (1.27 – 1.62)  1-19 vs. 20-32: 1.33 (1.17 – 1.50) | 24% |
| +Padilha ea. 2008 | 0: 987/1577  1-19: 919/1,524 | 301/768 | 0 vs. 20-32: 1.73 (1.42 – 2.10)  1-19 vs. 20-32: 1.60 (1.32 – 1.94) | 78% |
| +Ando ea. 2014 | 0: 1,133/3,190  1-19: 1,077/4,895 | 373/3,563 | 0 vs. 20-32: 2.02 (1.59 – 2.56)  1-19 vs. 20-32: 1.67 (1.32 – 2.11) | 88% |
| +Hu ea. 2015 | 0: 1,281/4,371  1-19: 1,845/13,862 | 2,987/49,066 | 0 vs. 20-32: 2.03 (1.68 – 2.47)  1-19 vs. 20-32: 1.63 (1.35 – 1.97) | 87% |
| +Joshy ea. 2016 | 0: 1,532/13,168  1-19: 2,601/55,298 | 3,794/166,530 | 0 vs. 20-32: 2.31 (1.80 – 2.95)  1-19 vs. 20-32: 1.78 (1.39 – 2.27) | 95% |
| +Hiratsuka ea. 2020 | 0: 1,607/13,315  1-19: 2,748/55,665 | 3,889/166,907 | 0 vs. 20-32: 2.27 (1.82 – 2.83)  1-19 vs. 20-32: 1.76 (1.41 – 2.19) | 94% |


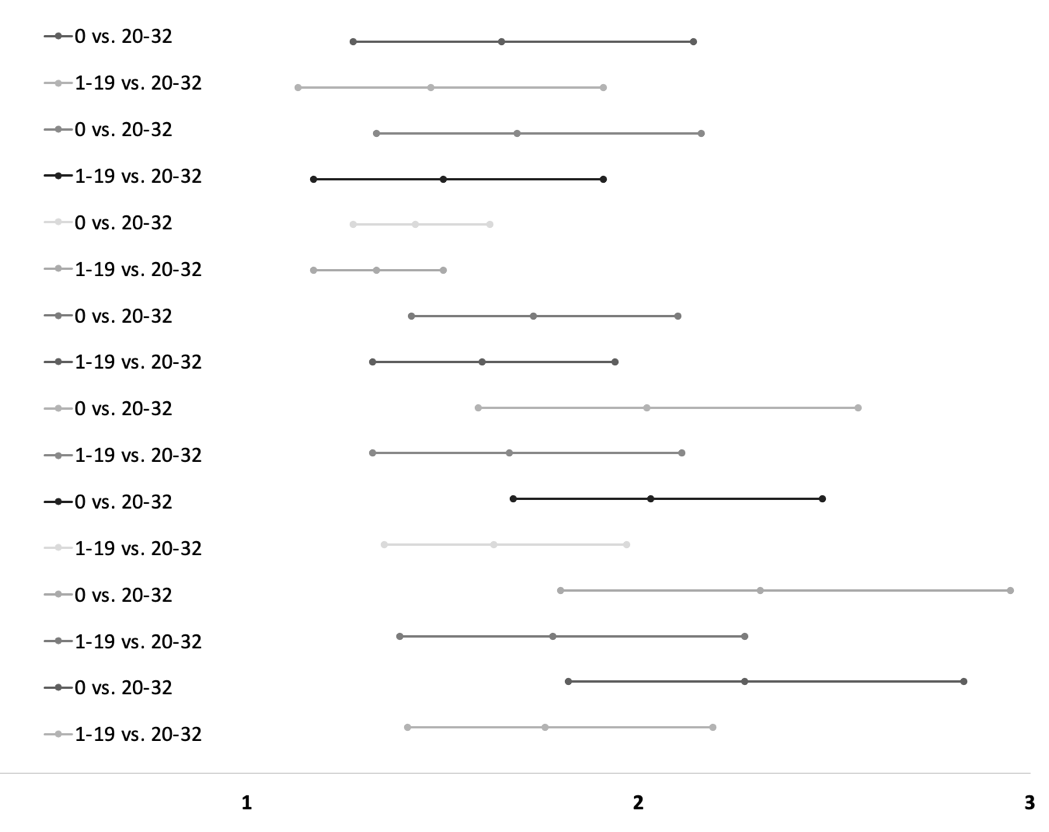


Abbreviations: RR, Risk Ratio; 95%-CI, 95%-Confidence Interval; vs., versus; ref., reference;

I^2^, I-square for heterogeneity; ACVD, Atherosclerotic Cardiovascular Disease; ACM, All-Cause Mortality; N.A., Not Applicable
