# Supplementary material for: Lower Number of Teeth Is Related to Higher Risks for ACVD and Death—Systematic Review and Meta-Analyses of Survival Data
Source: Front Cardiovasc Med. 2021 May 7;8:621626. doi: 10.3389/fcvm.2021.621626 (PMC8138430; doi:10.3389/fcvm.2021.621626)
Supplement: Supplementary file 13 [file Table_6.docx]

Supplementary Table 6: Cumulative meta-analysis for incidence density of ACVD-related events (morbidity or mortality) and All-Cause Mortality

| **Crude 0 vs. 1-32 teeth (ref.) (ACVD)** | | | |
| --- | --- | --- | --- |
| **Author/Year** | **Cumulative sample size** | **HR (95%-CI)** | **I^2^** |
| Ajwani ea. 2003 | 121 | 1.90 (1.06 – 3.39) | N.A. |
| +Brown ea. 2009 | 41,121 | 1.90 (1.80 – 2.01) | 0% |
| +LaMonte ea. 2017 | 98,122 | 1.89 (1.80 – 1.99) | 0% |
| **Adjusted by age/sex 0 teeth vs. 1-32 teeth (ref.) (ACVD)** | | | |
| Ajwani ea. 2003 | 121 | 1.53 (0.85 – 2.74) | N.A. |
| +Ragnarsson ea. 2004 | 2,734 | 1.72 (1.18 – 2.51) | 0% |
| +Dietrich ea. 2008 | 3,937 | 1.84 (1.39 – 2.43) | 0% |
| +Brown ea. 2009 | 44,937 | 1.49 (1.39 – 1.58) | 0% |
| **Multivariable 0 teeth vs. 1-32 teeth (ref.) (ACVD)** | | | |
| Ajwani ea. 2003 | 121 | 1.40 (0.76 – 2.59) | N.A. |
| +Ragnarsson ea. 2004 | 2,734 | 1.44 (0.97 – 2.13) | 0% |
| +Dietrich ea. 2008 | 3,937 | 1.55 (1.17 – 2.07) | 0% |
| +Brown ea. 2009 | 44,937 | 1.24 (1.15 – 1.33) | 0% |
| +LaMonte ea. 2017 | 101,938 | 1.21 (1.07 – 1.36) | 35% |


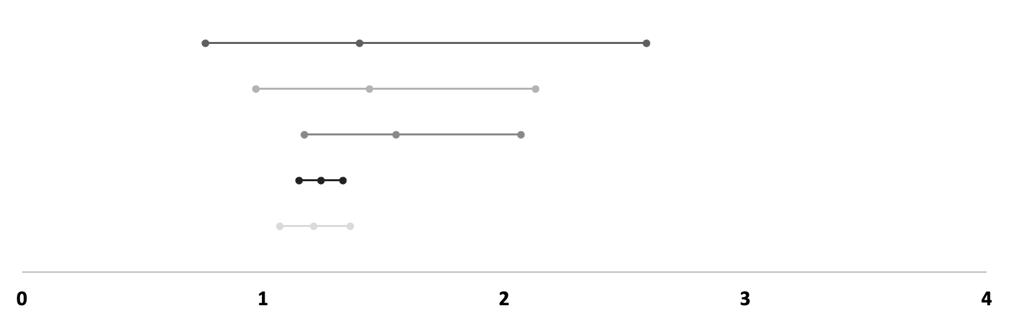

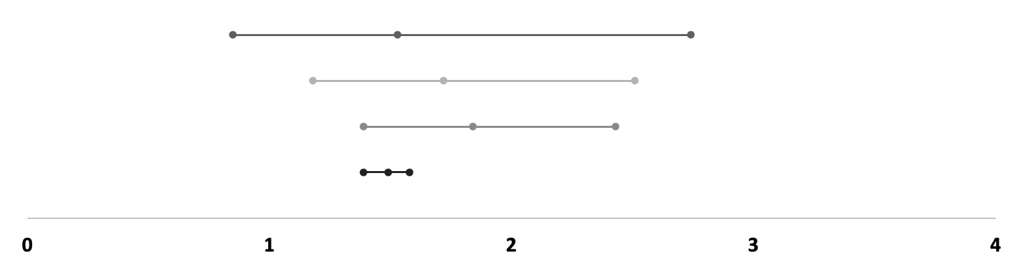

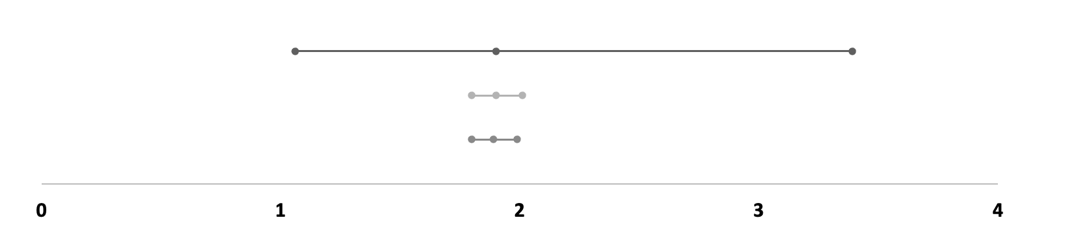


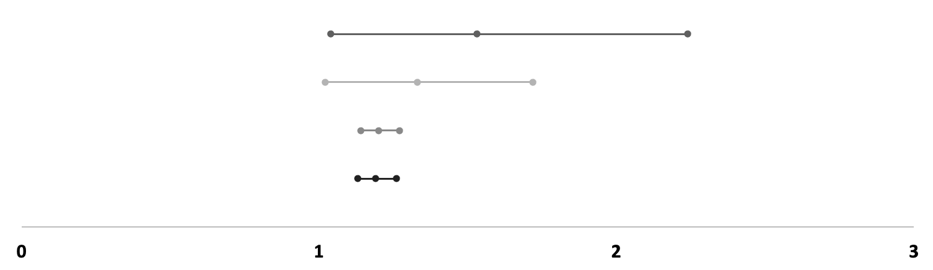

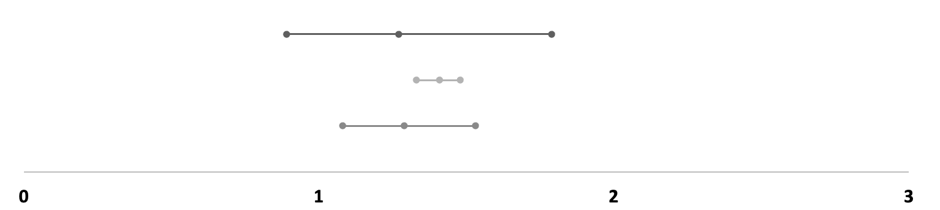


| **Crude 0-19 teeth vs. 20-32 teeth (ref.) (ACVD)** | | | |
| --- | --- | --- | --- |
| **Author/Year** | **Cumulative sample size** | **HR (95%-CI)** | **I^2^** |
| Aida ea. 2011 | 4,380 | 2.27 (1.50 – 3.43) | N.A |
| **Adjusted by age/sex 0-19 teeth vs. 20-32 teeth (ref.) (ACVD)** | | | |
| Ando ea. 2014 | 7,779 | 1.27 (0.89 – 1.79) | N.A |
| +Joshy ea. 2016 | 175,476 | 1.41 (1.33 – 1.48) | 0% |
| +Goto ea. 2020 | 186,749 | 1.29 (1.08 – 1.53) | 58% |
| **Multivariable 0-19 teeth vs. 20-32 teeth (ref.) (ACVD)** | | | |
| Aida ea. 2011 | 4,380 | 1.53 (1.04 – 2.24) | N.A |
| +Ando ea. 2014 | 12,159 | 1.33 (1.02 – 1.72) | 0% |
| +Joshy ea. 2016 | 179,856 | 1.20 (1.14 – 1.27) | 0% |
| +Goto ea. 2020 | 191,129 | 1.19 (1.13 – 1.26) | 0% |

| **Crude number of lost teeth (ACVD)** | | | |
| --- | --- | --- | --- |
| **Author/Year** | **Cumulative sample size** | **HR (95%-CI)** | **I^2^** |
| Qi ea. 2020 | 1,385 | 1.01 (0.99 – 1.03) | N.A. |
| **Adjusted by age/sex number of lost teeth (ACVD)** | | | |
| Joshipura ea. 2003 | 40,679 | 1.33 (1.02 – 1.74) | N.A. |
| +Hung ea. 2003 | 84,926 | 1.42 (1.18 – 1.71) | 0% |
| +Ragnarsson ea. 2004 | 87,539 | 1.24 (0.95 – 1.62) | 83% |
| +Hung ea. 2004 | 128,946 | 1.14 (0.98 – 1.32) | 75% |
| +Lee ea. 2019 | 4,533,916 | 1.03 (1.00 – 1.07) | 69% |
| +Goto ea. 2020 | 4,545,189 | 1.02 (1.00 – 1.04) | 81% |
| +Qi ea. 2020 | 4,546,574 | 1.01 (1.00 – 1.03) | 79% |
| **Multivariate number of lost teeth (ACVD)** | | | |
| Joshipura ea. 2003 | 40,679 | 1.27 (0.97 – 1.67) | N.A. |
| +Hung ea. 2003 | 84,926 | 1.33 (1.10 – 1.61) | 0% |
| +Ragnarsson ea. 2004 | 87,539 | 1.18 (0.94 – 1.47) | 75% |
| +Hung ea. 2004 | 128,946 | 1.08 (0.95 – 1.23) | 67% |
| +Abnet ea. 2005 | 157,736 | 1.12 (0.99 – 1.26) | 87% |
| +Tu ea. 2007 | 168,328 | 1.07 (1.01 – 1.14) | 85% |
| +Reichert ea. 2015 | 178,920 | 1.05 (1.00 – 1.10) | 83% |
| +Oluwagbemigun ea. 2015 | 203,233 | 1.04 (1.00 – 1.07) | 80% |
| +Kebede ea. 2017 | 206,560 | 1.03 (1.00 – 1.06) | 77% |
| +Vedin ea. 2017 | 222,016 | 1.03 (1.01 – 1.06) | 77% |
| +Lee ea. 2019 | 4,626,986 | 1.03 (1.01 – 1.04) | 76% |
| +Goto ea. 2020 | 4,638,259 | 1.02 (1.01 – 1.03) | 77% |
| +Qi ea. 2020 | 4,639,644 | 1.02 (1.01 – 1.03) | 75% |


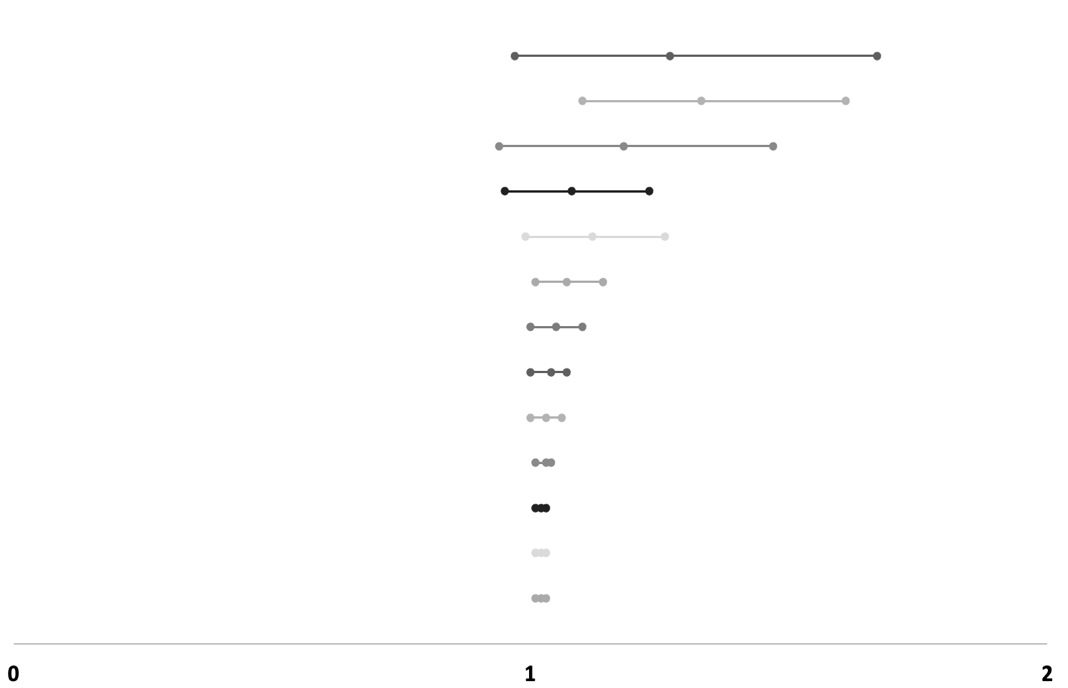

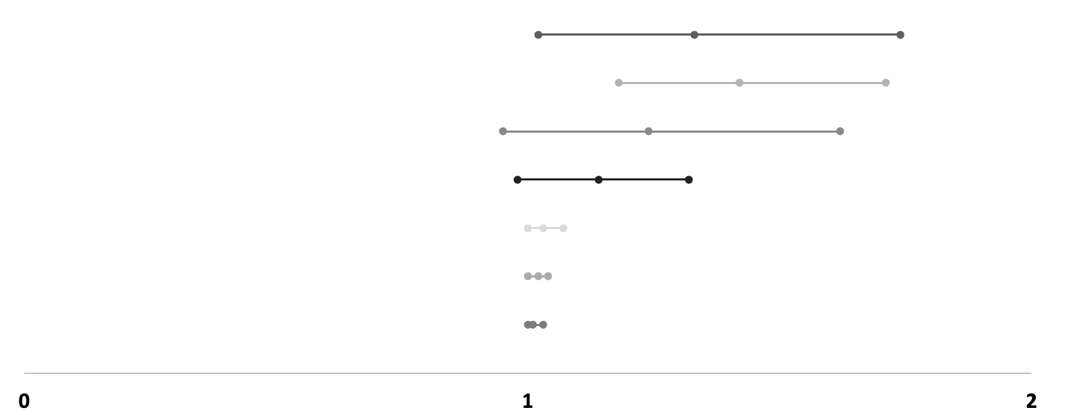


| **Crude 0 teeth vs. 1-32 teeth (ref.) (ACM)** | | | |
| --- | --- | --- | --- |
| **Author/Year** | **Cumulative sample size** | **HR (95%-CI)** | **I^2^** |
| Ajwani ea. 2003 | 121 | 1.85 (1.21 – 2.82) | N.A. |
| +Holm-Pedersen ea. 2008 | 694 | 1.64 (1.38 – 1.94) | 0% |
| +Brown ea. 2009 | 41,694 | 1.89 (1.47 – 2.44) | 83% |
| +LaMonte ea. 2017 | 98,695 | 2.00 (1.75 – 2.27) | 74% |
| +Nomura ea. 2020 | 99,303 | 1.73 (1.41 – 2.13) | 91% |
| **Adjusted by age/sex 0 teeth vs. 1-32 teeth (ref.) (ACM)** | | | |
| Ajwani ea. 2003 | 121 | 1.58 (1.03 – 2.41) | N.A. |
| +Ragnarsson ea. 2004 | 2,734 | 1.41 (1.16 – 1.73) | 0% |
| +Brown ea. 2009 | 43,734 | 1.56 (1.48 – 1.65) | 0% |
| **Multivariate 0 teeth vs. 1-32 teeth (ref.) (ACM)** | | | |
| Ajwani ea. 2003 | 121 | 1.48 (0.95 – 2.31) | N.A. |
| +Ragnarsson ea. 2004 | 2,734 | 1.25 (1.01 – 1.53) | 0% |
| +Holm-Pedersen ea. 2008 | 3,307 | 1.25 (1.08 – 1.45) | 0% |
| +Brown ea. 2009 | 44,307 | 1.33 (1.25 – 1.41) | 0% |
| +Hoke ea. 2011 | 44,718 | 1.33 (1.26 – 1.42) | 0% |
| +LaMonte ea. 2017 | 101,719 | 1.29 (1.18 – 1.40) | 27% |
| +Caplan ea. 2017 | 102,254 | 1.30 (1.21 – 1.40) | 20% |


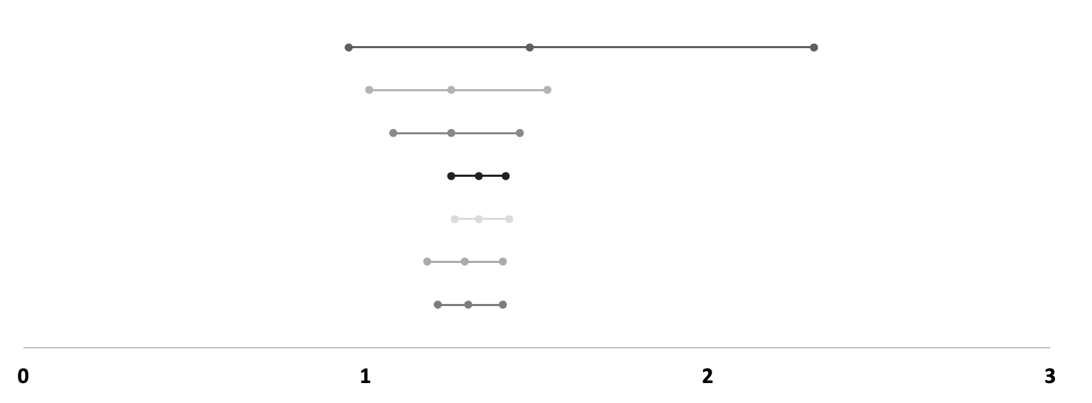

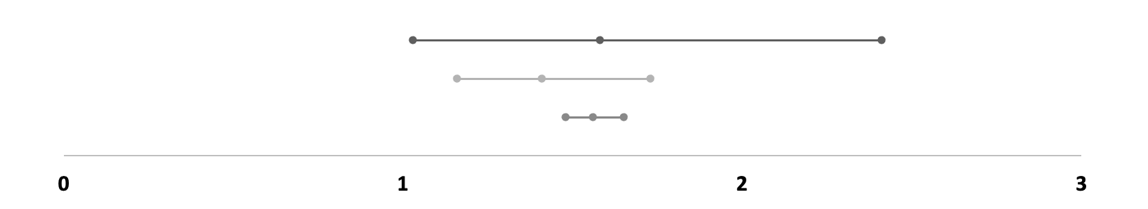

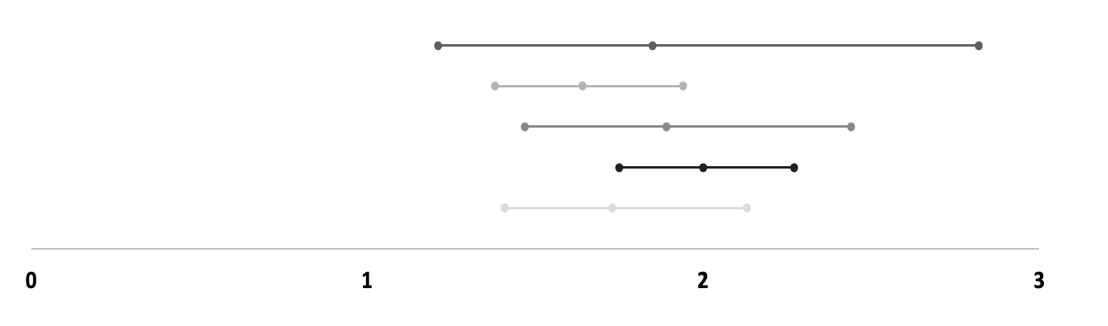


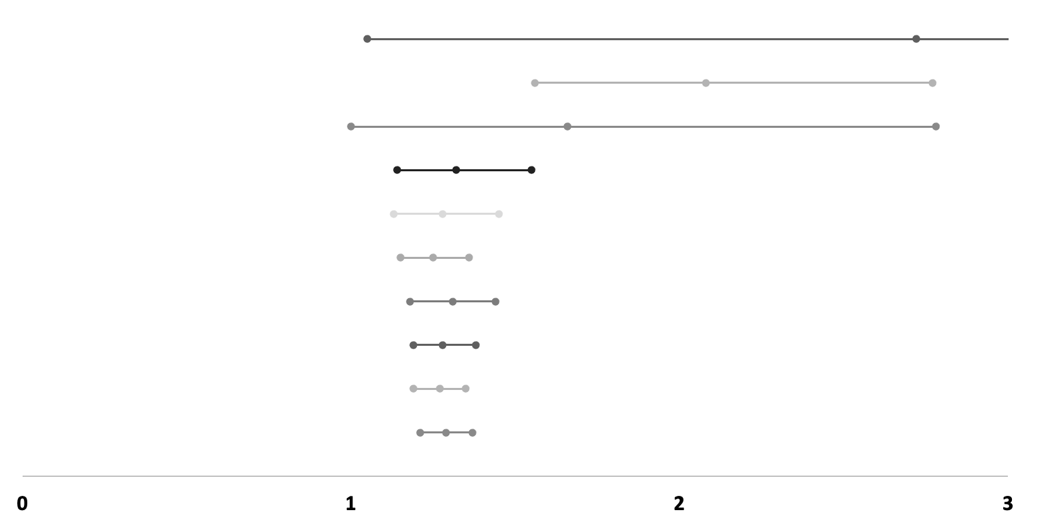

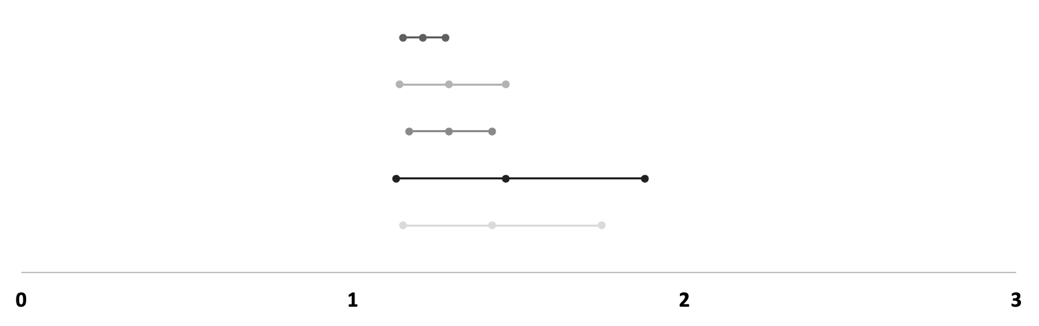

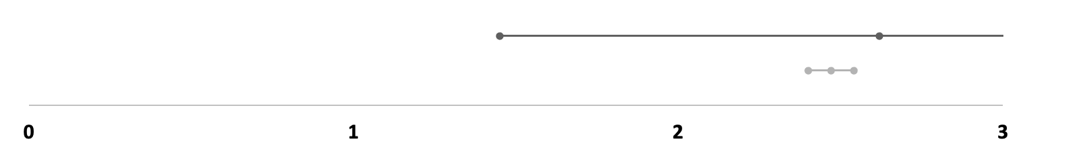


| **Crude 0-19 teeth vs. 20-32 teeth (ref.) (ACM)** | | | |
| --- | --- | --- | --- |
| **Author/Year** | **Cumulative sample size** | **HR (95%-CI)** | **I^2^** |
| Hamalainen ea. 2003 | 226 | 2.62 (1.45 – 4.72) | N.A. |
| +Yuan ea. 2020 | 36379 | 2.47 (2.40 – 2.54) | 0% |
| **Adjusted by age/sex 0-19 teeth vs. 20-32 teeth (ref.) (ACM)** | | | |
| Paganini-Hill ea. 2011 | 5,611 | 1.21 (1.15 – 1.28) | N.A. |
| +Hayasaka ea. 2013 | 25,908 | 1.29 (1.14 – 1.46) | 86% |
| +Ando ea. 2014 | 33,687 | 1.29 (1.17 – 1.42) | 73% |
| +Joshy ea. 2016 | 201,384 | 1.46 (1.13 – 1.88) | 98% |
| +Goto ea. 2020 | 212,657 | 1.42 (1.15 – 1.75) | 97% |
| **Multivariate 0-19 teeth vs. 20-32 teeth (ref.) (ACM)** | | | |
| Morita ea. 2006 | 118 | 2.72 (1.05 – 7.05) | N.A. |
| +Padilha ea. 2008 | 618 | 2.08 (1.56 – 2.77) | 0% |
| +Paganini-Hill ea. 2011 | 6,229 | 1.66 (1.00 – 2.78) | 87% |
| +Hayasaka ea. 2013 | 26,526 | 1.32 (1.14 – 1.55) | 82% |
| +Ando ea. 2014 | 34,305 | 1.28 (1.13 – 1.45) | 76% |
| +Hu ea. 2015 | 89,956 | 1.25 (1.15 – 1.36) | 71% |
| +Joshy ea. 2016 | 257,653 | 1.31 (1.18 – 1.44) | 84% |
| +Yuan ea. 2020 | 293,806 | 1.28 (1.19 – 1.38) | 81% |
| +Goto ea. 2020 | 305,079 | 1.27 (1.19 – 1.35) | 79% |
| +Hiratsuka ea. 2020 | 305,970 | 1.29 (1.21 – 1.37) | 78% |


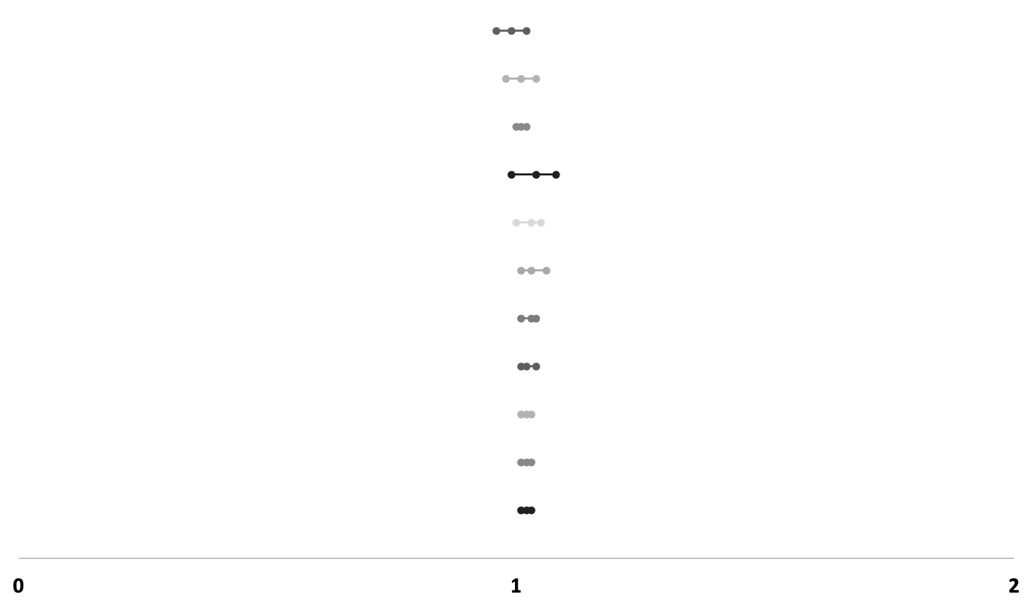

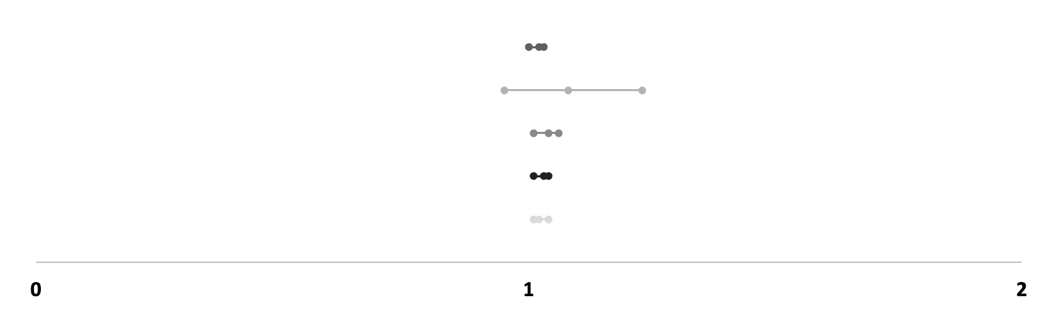

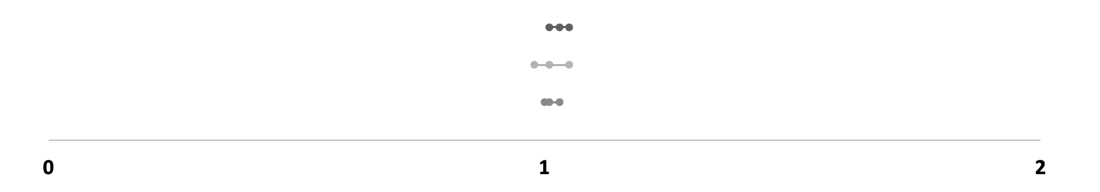


| **Crude number of lost teeth (ACM)** | | | |
| --- | --- | --- | --- |
| **Author/Year** | **Cumulative sample size** | **HR (95%-CI)** | **I^2^** |
| Hamalainen ea. 2003 | 226 | 1.03 (1.01 – 1.05) | N.A. |
| +Nomura ea. 2020 | 834 | 1.01 (0.98 – 1.05) | 87% |
| +Qi ea. 2020 | 2,219 | 1.01 (1.00 – 1.03) | 80% |
| **Adjusted by age/gender number of lost teeth (ACM)** | | | |
| Ragnarsson ea. 2004 | 2,613 | 1.02 (1.00 – 1.03) | N.A. |
| +Osterberg ea. 2007 | 3,617 | 1.08 (0.95 – 1.23) | 94% |
| +Lee ea. 2019 | 4,408,587 | 1.04 (1.01 – 1.06) | 89% |
| +Goto ea. 2020 | 4,419,860 | 1.03 (1.01 – 1.04) | 94% |
| +Qi ea. 2020 | 4,421,245 | 1.02 (1.01 – 1.04) | 93% |
| **Multivariate number of lost teeth (ACM)** | | | |
| Garcia ea. 1998 | 804 | 0.99 (0.96 – 1.02) | N.A. |
| +Hamalainen ea. 2003 | 1,030 | 1.01 (0.98 – 1.04) | 67% |
| +Ragnarsson ea. 2004 | 3,643 | 1.01 (1.00 – 1.02) | 34% |
| +Abnet ea. 2005 | 32,433 | 1.04 (0.99 – 1.08) | 91% |
| +Tu ea. 2007 | 43,981 | 1.03 (1.00 – 1.05) | 89% |
| +Osterberg ea. 2007 | 44,985 | 1.03 (1.01 – 1.06) | 88% |
| +Osterberg ea. 2008 | 46,366 | 1.03 (1.01 – 1.04) | 86% |
| +Kebede ea. 2017 | 49,693 | 1.02 (1.01 – 1.04) | 84% |
| +Lee ea. 2019 | 4,454,663 | 1.02 (1.01 – 1.03) | 84% |
| +Goto ea. 2020 | 4,465,936 | 1.02 (1.01 – 1.03) | 90% |
| +Qi ea. 2020 | 4,467,321 | 1.02 (1.01 – 1.03) | 89% |

Abbreviations: HR, Hazard Ratio; 95%-CI, 95%-Confidence Interval; vs., versus; ref., reference; I^2^, I-square for heterogeneity; ACVD, Atherosclerotic Cardiovascular Disease; ACM, All-Cause Mortality; N.A., Not Applicable
